# Functional Genomic Screening Identifies lncRNA CNPY2-AS1 as a Regulator of Redox Metabolism and Antiviral Immunity

**DOI:** 10.7150/ijbs.132728

**Published:** 2026-09-03

**Authors:** Carolina Khoury, Tahleel Ali-Nasser, Yuval Ben Eliahu, Haya Dahamshy Silawi, Liran Ben Yaakov, Simon Rousseau, Yvan Devaux, Nisrine Lahoud-Jeries, Assaf C. Bester

**Affiliations:** 1 Department of Biology, Technion-Israel Institute of Technology, 3200003, Haifa, Israel.; 2 Cardiovascular Research Unit, Department of Precision Health, Luxembourg Institute of Health, 1A-B rue Edison, L-1445 Strassen, Luxembourg.; 3 The Meakins-Christie Laboratories at the Research Institute of the McGill University Health Center, & Department of Medicine, Faculty of Medicine, McGill University, Montréal, QC, Canada.

**Keywords:** long non-coding RNA (lncRNA), SARS-CoV-2, innate immunity, interferon-stimulated genes (ISGs), redox homeostasis

## Abstract

The human genome encodes thousands of long non-coding RNAs (lncRNAs) that regulate innate immunity and viral life cycles, yet their roles in viral entry remain understudied. Here, we conducted a genome-wide CRISPR interference screen to identify lncRNAs that regulate SARS-CoV-2 entry. We identified two lncRNAs that modulate angiotensin-converting enzyme 2 (ACE2) dependent viral entry through distinct mechanisms. Our findings suggest that the RP11-314A20.5 locus acts as a *cis*-regulatory element that modulates the expression of neighboring genes, including MED11 and CXCL16, linking this genomic region to host pathways associated with SARS-CoV-2 entry and COVID-19 severity. In contrast, CNPY2-AS1 functions in trans as a central regulator coupling cellular redox homeostasis to innate immune signaling. Mechanistically, CNPY2-AS1 associates with thioredoxin reductase 1 (TXNRD1), a key enzyme that limits reactive oxygen species. Loss of CNPY2-AS1 disrupts redox balance, triggering ligand-independent STAT1 activation and IRF7-mediated interferon and inflammatory responses. This dysregulated program has dual effects: induction of the interferon-stimulated gene PLSCR1 restricts viral entry by reducing cell-surface ACE2, while concurrent cytokine activation recapitulates features of pathological inflammation in severe COVID-19. Consistently, clinical datasets show reduced CNPY2-AS1 expression in patients with fatal disease. Together, these findings reveal distinct lncRNA-mediated mechanisms regulating SARS-CoV-2 entry and identify CNPY2-AS1 as a critical integrator of redox metabolism and innate antiviral immunity.

## Introduction

Long non-coding RNAs (lncRNAs) constitute the largest class of non-coding RNAs in the human genome and are increasingly recognized as critical regulators of cellular homeostasis 1,2. Beyond their established roles in transcriptional and epigenetic regulation, lncRNAs have emerged as key modulators of signaling pathways governing immune responses and host pathogen interactions 3-7. Despite their abundance and functional diversity, the mechanisms by which lncRNAs integrate metabolic cues with innate immune signaling remain incompletely understood. Accumulating evidence indicates that lncRNAs participate in viral infection by modulating receptor expression, interferon responses, inflammatory signaling, and direct virus host interactions 8-13.

Several lncRNAs regulate STAT and IRF dependent transcriptional programs by acting as molecular scaffolds or decoys for immune signaling proteins 14-17. However, most insights into lncRNA function during viral infection derive from transcriptomic association studies, and systematic functional interrogation of lncRNAs in antiviral defense remains limited.

The Coronavirus disease 2019 (COVID-19) pandemic, caused by Severe Acute Respiratory Syndrome Coronavirus 2 (SARS-CoV-2), highlighted the importance of host determinants governing viral entry and innate immune activation. SARS-CoV-2 entry is initiated by binding of the viral spike protein to the ACE2 receptor 18,19, with additional contributions from host co-factors such as transmembrane protease serine 2 (TMPRSS2) and neuropilin-1^20^,^21^. While protein-coding host factors required for SARS-CoV-2 entry and replication have been extensively characterized 22, the contribution of non-coding regulatory RNAs to viral entry and early antiviral responses remains poorly defined.

Consistent with this knowledge gap, large-scale transcriptomic and single-cell studies have revealed widespread dysregulation of lncRNAs in COVID-19 patients, with expression patterns correlating with disease severity and inflammatory signatures (23-25. Notably, lncRNAs such as NEAT1^26^, MALAT1^27^, and HOTAIRM1^28^ have been implicated in inflammasome activation and cytokine production, highlighting the capacity of non-coding RNAs to shape antiviral and inflammatory responses. However, these studies are largely correlative, and whether and how lncRNAs functionally regulate SARS-CoV-2 entry and interferon signaling through defined molecular mechanisms remains unclear.

CRISPR-based functional genomics has emerged as a powerful approach for systematically identifying host factors involved in viral infection 29-36. Although genome-wide CRISPR screens have successfully uncovered protein-coding genes required for SARS-CoV-2 entry and replication 37,38, functional interrogation of lncRNAs using CRISPR interference (CRISPRi) remains underutilized. Extending CRISPRi screening to the non-coding genome therefore provides a unique opportunity to uncover previously unrecognized regulatory nodes linking metabolism, innate immunity, and viral susceptibility.

In this study, we performed a genome-wide CRISPRi screen to identify lncRNAs that regulate SARS-CoV-2 spike mediated entry. We identify the poorly characterized lncRNA RP11-977G19.11 (CNPY2-AS1) as a central regulator integrating cellular redox homeostasis with innate antiviral signaling. Mechanistically, CNPY2-AS1 associates with TXNRD1, thereby contributing to the maintenance of intracellular reactive oxygen species (ROS) levels and limiting ligand-independent activation of STAT1 and IRF7. Loss of CNPY2-AS1 amplifies interferon-stimulated genes (ISGs) expression, including PLSCR1, which restricts viral entry by reducing ACE2 availability at the plasma membrane while simultaneously promoting inflammatory cytokine production. Consistent with this dual antiviral and pro-inflammatory role, CNPY2-AS1 expression is reduced in patients with severe and fatal COVID-19. Collectively, these findings establish CNPY2-AS1 as a critical lncRNA-mediated node linking metabolic redox state to innate immune signaling and viral susceptibility.

## Materials and Methods

### Cell culture

In this study, the SNU-449, A549, and HEK293T cell lines were used. HEK293T cells, derived from human embryonic kidney cells, were maintained in Dulbecco's Modified Eagle Medium (DMEM; Biological Industries) supplemented with 10% fetal bovine serum (FBS), 1% L-glutamine, and 1% penicillin-streptomycin (pen-strep). SNU-449 (human hepatocellular carcinoma) and A549 (human lung carcinoma) cells were cultured in Roswell Park Memorial Institute (RPMI) 1640 medium (Gibco), supplemented with 10% FBS, 1% L-glutamine, and 1% pen-strep. All cell lines were passaged every 2-3 days and maintained at 37°C in a humidified incubator with 5% carbon dioxide (CO₂). Cell viability and counts were assessed using trypan blue exclusion with a LUNA Automated Cell Counter (Logos Biosystems). Routine mycoplasma testing was conducted to ensure cell culture quality.

### Generation of a stable cell line expressing ACE2, CRISPRi

Lentiviral vectors encoding ACE2-TMPRSS2-Blast (Supplementary Table 2), dCas9-mCherry-ZIM3-KRAB (Supplementary Table 2), were produced in HEK293T cells using the PolyJet in vitro transfection reagent (SigmaGen, Frederick, MD, USA), following the manufacturer's protocol. Transfection was performed using the psPAX2 (Supplementary Table 2) and VSV-G (Supplementary Table 2) lentiviral packaging plasmids. HEK293T cells were seeded 24 hours prior to transfection to achieve ~90% confluency at the time of transfection. Lentiviral particles were harvested 48 hours of post-transfection, and the culture medium was filtered through a 0.45 μm cell strainer.

SNU-449, HEK293T, and A549 cells were seeded at a density of 3 × 10⁵ cells/well in 6-well plates 24hr prior to transduction. Cells were transduced with lentiviral vectors in the presence of 10 µg/ml Polybrene (Sigma, TR-1003-G) to enhance infection efficiency. Transduced cell lines were enriched using antibiotic selection or fluorescence-activated cell sorting (FACS) performed on the FACS Aria III (BD Biosciences) or Bigfoot spectral cell sorter.

The 33 significant sgRNAs identified from the PinAPL-Py analysis were selected for validation. sgRNA oligonucleotides were ordered from IDT, annealed, and then ligated into the digested PSB700-puro plasmid. The ligated plasmids were transformed into E. coli DH5α competent cells via heat shock and selected on ampicillin-containing media. Colony PCR was performed using backbone-specific primers to identify positive colonies. DNA from the positive colonies was extracted using the Quick-DNA Miniprep Plus Kit (ZYMO Research, D4068), following the manufacturer's instructions.

### CRISPR-Cas9 gene knock out (KO)

To generate gene KO for three target genes (MED11, CNPY2-AS1, and PLSCR1), we employed the CRISPR-Cas9 system. SgRNAs were designed using CHOPCHOP or Benchling tools to ensure precise targeting (Supplementary Table 5).

For PLSCR1, sgRNAs were designed using the CHOPCHOP tool. For MED11, two sgRNAs were designed using CHOPCHOP to induce deletion within the first exon. For CNPY2-AS1, two sgRNAs flanking the 3′ acceptor site were designed using Benchling to achieve targeted deletion. The designed sgRNAs were cloned into the lenti-CRISPRv2 vector (Supplementary Table 2) for delivery and expression.

Each pair of gRNAs was transduced into SNU449-ACE2 cells as described above. Following transduction, antibiotic selection was performed using puromycin, and genomic DNA (gDNA) was extracted. Sanger sequencing was conducted to confirm successful KO. The PCR primers can be found in Table S4.

### RNA extraction and cDNA synthesis

Total RNA was extracted from transduced cells using the TRIzol™ Reagent (Invitrogen, 15596018) following the manufacturer's protocol. The extracted RNA was subsequently reversed transcribed into complementary DNA (cDNA) using the Quantabio qScript cDNA Synthesis Kit (QIAGEN, Beverley, MA, USA) according to the manufacturer's instructions. The cDNAs were then used for qPCR using qPCRBIO SyGreen Blue Mix Lo-ROX (PCR Bio-systems, London, UK), and expression was measured relative to that of cells transduced with a non-targeting sgRNA. Changes in gene expression were calculated using the standard 2-ΔΔCt method and normalized to PPIB and PGK1. The primers used for qPCR are listed in Supplementary Table 3.

### Pseudovirus production and titering

VSV-ΔG-G stock was generated by transfecting BHK-21/WI-2 cells with the VSV-G plasmid using PolyJet, following the manufacturer's instructions. At 24 hours of post-transfection, the media was removed and replaced with media supplemented with VSV-ΔG-G encoding GFP. The cells were incubated for 1 hour, then free viruses were washed off by rinsing twice with PBS. At 24 hours of post-infection, the supernatant was harvested, and cell debris was cleared by centrifugation at 2500 rpm for 10 minutes at 4°C. The supernatant was then aliquoted and stored at -80°C. To titer the VSV-ΔG-G virions, BHK-21 cells were seeded at 7.5 × 10³ cells/well in a 96-well plate. The following day, the media was replaced with 10-fold serial dilutions of the virus, ranging from 10⁻² to 10⁻⁹. At 24 hours of post-infection, cells were manually counted using a fluorescence microscope to calculate the viral titer.

### SARS-CoV-2 spike pseudotype infection

VSV-ΔG-Spike virions were produced as described in the previous method (Pseudovirus production and titering), except HEK293T cells were transfected with the Spike-Δ18-D614G plasmid (Supplementary Table 2). SNU-449-ACE2 cells were seeded at 3 × 10⁵ cells/well in a 6-well plate. The following day, the media was removed and replaced with the virus. Infection efficiency was measured 24 hours post-infection using flow cytometry on the BD LSR-II (BD Biosciences) instrument.

### Pooled CRISPRi screen

Jonathan Weissman's CRISPRi non-coding library (CRiNCL) contains 10 sgRNAs per transcription start site (TSS), targeting lncRNA genes common to seven cell lines (Supplementary Table 2). The library was delivered to SNU-449-KRAB-ACE2 cells by lentiviral transduction at ~0.3 Multiplicity of Infection (MOI). The library representation was kept at a 1000 cells/sgRNA ratio throughout the screening. Four days post-transduction, 2 ug/ml puromycin was added to the media, and the cells were selected for ten days. The cells were infected with the VSV-ΔG-S pseudotype at 24 h post-infection; the cells were sorted on the FACS Aria III Cell Sorter (BD Biosciences). Three fractions of cells were collected: the high and low 10% of GFP expressing cells, and a control fraction of cells with medium GFP expression. For all fractions, the number of cells collected was sufficient to maintain the library's representation. The screen was performed in four biological replicates.

### Illumina sequencing

Genomic DNA was extracted from the cells using the Quick DNA Miniprep Plus kit (Zymo Research), following the manufacturer's instructions. To construct Illumina libraries, PCR was performed using Q5 High-Fidelity 2X Master Mix with Illumina-compatible primers to amplify the sgRNA inserts while appending Illumina adaptors and barcodes to the amplicons. The amplicons were purified using the NucleoSpin Gel and PCR Clean-up Kit, and the quality and concentration of the purified libraries were assessed using Qubit and TapeStation. The libraries were then pooled and concentrated. The pooled library was sequenced as single-read 50 bp reads on the NextSeq2000 (Illumina). According to Jonathan Weissman's library preparation protocol, 250-500 reads per sgRNA per sample are suggested. Our library contained 13,000 sgRNAs across 12 samples, necessitating > 40 million reads dedicated to the sgRNA sequences, with 30% of the reads allocated to PhiX sequencing control. We performed the sequencing with 400 million reads, which exceeded the required number.

### PinAPL-Py screen analysis

Sequencing read alignment, read counting, quality control, and sgRNA enrichment analysis of the GFP high, low, and control fractions were performed using the PinAPL-Py web application 39. All analysis parameters were left in their default settings. Read counts were normalized using 258 non-targeting control sgRNAs provided in the CRiNCL library, employing the counts per million (CPM) method. P-value adjustment was performed using the Sidak correction method, with a significance threshold for sgRNA ranking set at 0.01. Additionally, R was used to assess sequencing quality by conducting Principal Component Analysis (PCA) on the 500 sgRNAs with the highest variance and by reviewing the read count distribution using box plots.

### MAGeCKFlute screen analysis

Sequencing read alignment, read counting, quality control, batch effect removal, and candidate gene identification were performed using the MAGeCKFlute tool 40. The Mageck count function was used to generate a read-count table, and read counts were normalized using 258 non-targeting sgRNAs. The ComBat function in R was employed to correct batch effects in the dataset. The mageck test function was then used to generate gene and sgRNA rankings using the MAGeCK RRA method, which ranks sgRNAs and genes based on p-values and false discovery rates (FDR) and utilizes a modified RRA algorithm to identify positively and negatively selected genes.

### Screen validation

Lentiviral vectors packaging the top sgRNAs were produced in HEK-293T cells as described previously. SNU-449-KRAB-ACE2 cells were then transduced and selected with 2 µg/ml puromycin for 10 days. The selected cells were subsequently infected with VSV-ΔG-S, and infection efficiency was measured 24 hours post-infection using flow cytometry on the BD LSR-II. Cells transduced with a non-targeting sgRNA served as a control. The infection was performed in two biological replicates.

### Cloning sgRNA sequence into plasmids

The sgRNA protospacer sequence was cloned into the pLentiCRISPR v2 plasmid (Supplementary Table 2). Approximately 100 ng of the plasmid was digested with the restriction enzyme Esp3I nuclease (NEB) according to the manufacturer's instructions. The sgRNA oligonucleotides were annealed and ligated into the digested plasmid using T4 DNA ligase (NEB). Following ligation, the plasmids were transformed into E. coli DH5α competent cells via heat shock. Transformed bacterial colonies were selected, and successful cloning was validated by PCR using specific primers designed to amplify the edited regions. The resulting PCR products were subjected to Sanger sequencing to confirm the presence of desired genetic modifications.

### ACE2 antibody binding assay

SNU-449-KRAB-ACE2, SNU-449-KRAB-ACE2- CNPY2-AS1 KO, and naïve SNU-449 cells were harvested and incubated with an anti-ACE2 primary antibody (1:500 dilution) at 4 °C for 30 minutes. Following incubation, cells were washed with ice-cold PBS supplemented with 10% FBS and 0.02% sodium azide. Subsequently, cells were incubated with an anti-rabbit Alexa Fluor® 594 conjugate secondary antibody (1:2000 dilution) (Supplementary Table 1) at 4 °C for 20 minutes.

After incubation, cells were washed and analyzed using a Novocyte flow cytometer (Agilent) to assess ACE2 expression. The specific antibodies used are provided in Table S1.

### Western blot (WB) analysis

To investigate the impact of CNPY2-AS1 KO on ACE2 receptor levels and TXNRD1 protein expression following genetic and pharmacological perturbation**,** total protein was extracted using a RIPA buffer supplemented with a protease inhibitor cocktail (PI**).** Protein concentration was quantified using the BCA protein assay kit (Sigma-Aldrich**,** St**.** Louis**,** MO**,** USA**),** following the manufacturer's instructions. Equal amounts of protein were resolved via 12% SDS-PAGE**,** and the separated proteins were transferred onto low-fluorescence polyvinylidene fluoride (LV-PVDF) membranes**.** The membranes were blocked with 5% skim milk in TBST (Tris-buffered saline with 0.1% Tween-20) and incubated overnight at 4°C with primary antibodies against ACE2 **(**Supplementary Table 1) and GAPDH (Supplementary Table 1). After washing, membranes were incubated with an HRP-conjugated goat anti-rabbit IgG secondary antibody (Supplementary Table 1) for 1 hour at room temperature. Protein levels were visualized using enhanced chemiluminescence (ECL), and quantification of ACE2 expression was performed using Evolution software, with normalization to GAPDH as a loading control.

### RNA sequencing

RNA sequencing was conducted for SNU449-ACE and SNU449-ACE- CNPY2-AS1 KO. Total RNA was extracted from three biological replicates per sample using the PureLink RNA Mini Kit (Invitrogen, Waltham, MA, USA). The quality of the extracted RNA was assessed using the TapeStation 4200 (Agilent) with the RNA kit (Agilent, cat no. 5067-5576). All samples demonstrated high integrity (RIN 10).

RNA-seq library preparation was performed using the NEBNext UltraExpress RNA Library Prep Kit for Illumina (NEB, cat no. E3330), with 100 ng of total RNA as the starting material. Poly(A) mRNA enrichment was achieved using the NEBNext® Poly(A) mRNA Magnetic Isolation Module (NEB, cat no. E7490). Library quality control (QC) was conducted by measuring library concentration with Qubit (Invitrogen) and the Equalbit dsDNA HS Assay Kit (Vazyme, cat no. EQ121), as well as determining fragment size using the TapeStation 4200 (Agilent) with the High Sensitivity D1000 kit (Agilent, cat no. 5067-5584). All libraries were pooled at equal molarity into a single tube for sequencing. RNA sequencing was performed on the Illumina NextSeq2000 platform using the P4 XLEAP-SBS Reagent Kit (50 cycles) (Read1-72; Index1-8; Index2-8) (Illumina, cat no. 20100995).

The raw sequencing data was processed on the Galaxy web platform (usegalaxy.org). FASTQ Groomer was used to convert raw files to FASTQ format; alignment was carried out using HISAT2, and read counts were generated using featureCounts. Differential gene expression analysis was conducted using the DESeq2 package in R studio (version 4.4.1).

### Immunofluorescence (IF)

IF was performed to assess the subcellular localization and activation of STAT1 and TXNRD1 in SNU449 cells. Briefly, cells were seeded on coverslips at a density of 2-2.5 × 10⁵ cells/mL in 1 mL of complete medium per well, and for cytokine stimulation experiments, medium was replaced with serum-free medium 24 h prior to treatment. Cells were stimulated with IFN-γ (Abcam, ab259377) at a final concentration of 0.1mg/mL for 15,30, and 60 min, followed by washing with PBS containing 0.9 mM CaCl₂ and 0.5 mM MgCl₂. Fixation was performed with 4% paraformaldehyde for 20 min at room temperature, followed by three washes with PBS. Cells were then permeabilized and blocked in PBS containing 0.1% Triton X-100, 5% normal serum (Abcam, ab7475), and 2.5% BSA for 1 h at room temperature. Primary antibodies were diluted in blocking buffer and applied overnight at 4 °C, and coverslips were inverted onto drops of antibody solution on parafilm to minimize reagent use. Following washes, cells were incubated with Alexa Fluor 647-conjugated secondary antibodies (Supplementary Table 1) (1:400 in blocking buffer) for 1 h at room temperature in the dark, washed, and nuclei were stained with DAPI 1mg (1:500 dilution) for 5 min. Coverslips were mounted on pre-cleaned slides using Fluoromount-G and imaged using confocal microscopy after sealing with nail polish. Washing and blocking buffers were prepared using PBS and Triton X-100 (0.1%), with BSA and host-matched normal serum added for blocking. Confocal images were acquired using ZEN software and quantified with Imaris. Antibodies used were STAT1and TXNRD1 (IF 1:300 dilution).

### Flow cytometric measurement of intracellular ROS using DCFDA/H2DCFDA

Intracellular ROS levels were measured using the DCFDA/H2DCFDA Cellular ROS Assay Kit (Abcam, ab113851) according to the manufacturer's instructions. Briefly, SNU449 cells were harvested and resuspended in serum-free medium, then incubated with 20 µM DCFDA for 30-45 min at 37 °C in the dark. After incubation, cells were washed with PBS and immediately analyzed by flow cytometry. Data were collected for at least 10,000 events per sample, and ROS levels were quantified as mean fluorescence intensity relative to untreated controls.

### Hydrogen peroxide (H₂O₂) treatment

SNU449 cells were seeded at a density of 3 × 10⁵ cells/mL and allowed to adhere overnight. Cells were then treated with Hydrogen peroxide 30% (Biosolve Chimie, Netherlands; cat. no. 2697) at a final concentration of 150 µM for 24 h to induce oxidative stress.

### RNA pull-down and mass spectrometry analysis

Cells were lysed on ice for 30 min in NP-40 lysis buffer (150 mM NaCl, 1% NP-40, 20 mM Tris-HCl, pH 8.0) supplemented with fresh protease and phosphatase inhibitors. Lysates were clarified by centrifugation at 10,000 × g for 10 min at 4 °C, and the supernatant (NP-40 soluble fraction) was collected. The pellet was washed once with NP-40 lysis buffer and then subjected to a hot lysis protocol to extract chromatin bound proteins. For hot lysis, the pellet was resuspended in hot lysis buffer, incubated at 100 °C for 15 min, cooled on ice, and briefly sonicated (2 × 15 s pulses at 35% amplitude) before centrifugation at 14,000 × g for 20 min. The resulting supernatant (chromatin-bound protein fraction) was collected and stored at 80 °C. The NP-40 soluble protein fraction (supernatant) was used as input for the RNA pull-down assays. Biotinylated RNA was generated by in vitro transcription using T7 RNA polymerase (New England Biolabs, M0251S) in the presence of Biotin RNA Labeling Mix (Roche, 11685597910). Following transcription, DNA templates were removed by RNase-free DNase I treatment, and biotin-labeled RNA was purified using the NucleoSpin RNA Clean-up kit (Macherey-Nagel, Cat# 740609.250) according to the manufacturer's instructions.

Hydrophilic streptavidin magnetic beads (New England Biolabs, S1421S) were washed twice with Buffer A (0.1 M NaOH, 0.05 M NaCl), once with Buffer B (0.05 M NaCl), and equilibrated in Binding/Washing Buffer (5 mM Tris-HCl pH 7.5, 0.5 mM EDTA, 1 mM DTT) supplemented with protease inhibitor cocktail and RNase inhibitor. For each pull-down reaction, 1 µg of biotinylated RNA was incubated with 50 µL of streptavidin beads for 30 min on ice with gentle agitation to allow RNA immobilization. NP-40 soluble protein lysates (500 ng total protein) were incubated with RNA-coated beads for 30 min on ice with rotation. Beads were subsequently washed, and bound proteins were eluted twice using ChIP Elution Buffer (10 mM HEPES pH 7.5, 3 mM MgCl₂, 250 mM NaCl, 1 mM DTT, 10% glycerol). All pull-down experiments were performed in four independent biological replicates.

Eluted proteins were digested with sequencing-grade trypsin and analyzed by liquid chromatography tandem mass spectrometry (LC-MS/MS) using an Orbitrap Exploris 480 mass spectrometer (Thermo Fisher Scientific) at the Smoler Proteomics Center. Raw data were processed using MaxQuant (v2.6) with a 1% false discovery rate at the peptide and protein levels. Protein intensities were log2-transformed, missing values were imputed using the minimal signal intensity across the dataset, and Gene Ontology annotations were used to identify RNA-binding proteins.

### TRI-1 treatment

Cells were seeded one day prior to treatment at a density of 3 × 10⁵ cells/mL in complete culture medium and allowed to adhere overnight. TRI-1 treatment was performed by incubating cells with 2 µM TRI-1 for 2 h, followed by replacement with fresh complete medium and a 24 h recovery period prior to downstream analyses. TRI-1 (MedChemExpress, HY-125006) was used to pharmacologically inhibit TXNRD1 activity.

### Pharmacological modulation of antioxidant pathways using DMF and sodium selenite

Cells were seeded one day prior to treatment at a density of 3 × 10⁵ cells/mL in complete culture medium and allowed to adhere overnight. For pharmacological and metabolic modulation experiments, cells were treated by directly supplementing the culture medium with dimethyl fumarate (DMF) (50 µM final concentration; Sigma, Cat. No. 227056) or sodium selenite (100 nM final concentration; Sigma, Cat. No. S5261) for 24 hours under standard culture conditions. Following treatment, cells were harvested for downstream analyses, including ROS measurement, viral infection assays, and signaling analysis, as indicated.

### siRNA-mediated knockdown of TXNRD1

TXNRD1 knockdown (KD) in SNU449 cells was performed using MISSION® esiRNA targeting human TXNRD1 (Cat. No. EHU018281, Sigma-Aldrich). The lyophilized esiRNA powder, supplied at 20 µg, was resuspended in 69 µL TE buffer to generate a 20 µM stock solution, based on an average molecular weight of 14,490 g/mol for 21-mer siRNA duplexes. The siRNA stock was aliquoted and stored at -20°C until use. SNU449 cells were seeded prior to transfection to reach 70%-80% confluency at the time of transfection. For each well, 3.75 µL of the 20 µM TXNRD1 esiRNA stock was used, corresponding to 75 pmol siRNA per well. Transfection was performed using Lipofectamine 3000 according to the manufacturer's instructions.

### Rescue of lncRNA expression by stable plasmid overexpression

The expression of selected lncRNAs was rescued by stable transduction of the mature lncRNA sequence RP11-314A20.5 gene and the coding sequence of MED11 gene into cells with confirmed MED11 KO of the gene. The lncRNA sequences can be found in Supplementary Table 6, were synthesized by Twist Bioscience (San Francisco, CA, USA) in their Twist Cloning Vector. The sequences were then ligated into N174-MCS (kindly provided by Adam Karpf; (Supplementary Table 2). Lentiviral transduction was performed as described above into SNU449-ACE cells with confirmed KO of the same MED11, followed by antibiotic selection with 600 μg/ ml Geneticin (G418; Invivogen, Toulouse, France) for 7-10 days.

### Cell fractionation into cytoplasmic and nuclear fractions

Cytoplasmic and nuclear fractions were separated using a detergent-based cell fractionation procedure. Approximately (2 \times 10^6) cells were collected and resuspended in 1 mL of ice-cold PBS. Cells were centrifuged at 600 × g for 5 min at room temperature, and the supernatant was removed. The cell pellet was gently resuspended in 150 µL ice-cold Buffer A, followed by the addition of 150 µL ice-cold 2× lysis buffer. The suspension was mixed gently by inversion and incubated on ice for 8 min. Following lysis, the samples were centrifuged at 600 × g for 5 min at room temperature.

The supernatant (200 µL) was carefully collected without disturbing the nuclear pellet and designated as the cytoplasmic fraction. The remaining supernatant was removed, and the nuclear pellet was gently resuspended in 1 mL ice-cold RLN buffer and incubated on ice for 5 min. During this incubation, the cytoplasmic fraction was centrifuged at 500 × g for 1 min to remove residual nuclei and cellular debris. The resulting supernatant was transferred to a new tube. For RNA extraction, 1 mL TRI reagent was added to the clarified cytoplasmic fraction. The nuclear fraction was subsequently centrifuged at 1000 × g for 5 min; the supernatant was discarded, and the nuclear pellet was lysed in 1 mL TRI reagent. RNA was then extracted from both cytoplasmic and nuclear fractions according to the manufacturer's standard TRI reagent protocol. Buffer A consists of 15 mM Tris-Cl (pH 8.0), 15 mM NaCl, 60 mM KCl, 1 mM EDTA (pH 8.0), 0.5 mM EGTA (pH 8.0), and 0.5 mM spermidine. The 2× lysis buffer was prepared by supplementing Buffer A with 0.5% NP-40. RLN buffer contained 50 mM Tris-Cl (pH 8.0), 140 mM NaCl, 1.5 mM MgCl₂, 0.5% NP-40, and 10 mM EDTA. RNase inhibitor was added fresh to the buffers immediately before use.

### Statistical Analysis

Statistical analyses were performed using the rstatix package in R (version 4.4.1). Values are given as the mean±SD unless otherwise stated. Significance was evaluated using a two-tailed Student's t-test with Benjamini- Hochberg for correction unless otherwise stated. The value of p<0.05 was considered statistically significant.

## Results

### Establishing a Functional Screening Model for SARS-CoV-2 Cell Entry

To investigate how lncRNAs regulate SARS-CoV-2 entry, we established a CRISPRi-based forward genetic screening platform. We first optimized both cellular and viral models suitable for large-scale functional screening.

To model viral entry, we employed a single-cycle VSV-ΔG pseudovirus system expressing the SARS-CoV-2 spike protein. In this recombinant vesicular stomatitis virus (VSV), the native glycoprotein (G) is replaced (ΔG) with the SARS-CoV-2 spike protein 41, enabling the study of spike-mediated entry under biosafety level 2 (BSL-2) conditions. The VSV genome additionally encodes green fluorescent protein (GFP), allowing quantitative detection and assessment of infection efficiency (Fig. 1a).

We initially evaluated infection efficiency in HEK293T cells expressing human ACE2 and the TMPRSS2 cofactor 42, either stably or via transient transfection. Using the wild-type (WT) SARS-CoV-2 spike protein, we observed relatively low infection rates (~2-9%) in transiently transfected HEK293T cells (Fig. S1a), limiting the feasibility of large-scale CRISPRi screening. To enhance viral entry, we tested two spike variants: an 18 amino acid deletion in the cytoplasmic tail (Δ18), which increases spike surface expression and reduces lysosomal degradation (43, and the Δ18 variant combined with an aspartic acid to glycine substitution at position 614 (D614G). This variant first emerged in Europe and increases spike density and binding affinity to the ACE2 receptor. Consistent with prior observations, both variants exhibited an approximately five-fold increase in infection efficiency compared to the WT spike (Fig. S1a,b).

Although SARS-CoV-2 primarily targets the respiratory system, infection of extrapulmonary tissues has been increasingly recognized and is associated with severe complications 44. SARS-CoV-2 associated liver injury, termed SARS-CoV-2 induced hepatopathy (SIH), can be pronounced in patients with underlying liver disease 45. Despite its clinical relevance, genetic factors influencing viral infection in hepatic cells remain poorly defined, particularly with respect to non-coding RNAs.

Following the testing of several cell lines, we observed the highest infection efficiency in SNU449 hepatocellular carcinoma cells stably expressing ACE2 and TMPRSS2 (Fig. 1b, c; Fig. S1). Notably, parental SNU449 cells lack endogenous ACE2 expression and were resistant to infection by all VSV-Spike variants, indicating strict ACE2-dependent viral entry in this system.

Therefore, to shed light on the role of lncRNAs in SARS-CoV-2 infection, we established an efficient CRISPRi screening platform. We next compared two dCas9-based transcriptional repression constructs: dCas9 fused to the KRAB domain (dCas9-BFP-KRAB) 46 and dCas9 fused to both KRAB and ZIM3 (dCas9-mCherry-KRAB-ZIM3) 47. As recently published, incorporation of the ZIM3 domain significantly enhanced KD efficiency of selected target genes, achieving an approximately two-fold improvement relative to KRAB alone (Fig. S1).

Based on physiological relevance and system optimization, SNU449-ACE2-dCas9-KRAB-ZIM3 cells in combination with the VSV-GFP-Δ18-D614G spike pseudovirus were selected as the platform for subsequent CRISPRi screening.

### CRISPRi screening for lncRNAs affecting SARS-CoV-2 cell entry

To identify new non-coding genes affecting SARS-CoV-2 infection, we first established a stable SNU449-ACE2-dCas9-KRAB-ZIM3 cell line. These cells were transduced with a library of single guide RNA (sgRNA) targeting 1329 commonly expressed lncRNAs with high coverage (1000 cells/sgRNA) using a low MOI<0.3 of lentiviral vectors. After successful puromycin selection, the cells were infected with a high MOI of VSV-GFP-Δ18-D614G spike.

Twenty-four hours after pseudovirus infection, cells were sorted based on GFP fluorescence intensity into three populations: the top 10% GFP-high, the bottom 10% GFP-low, and the intermediate 80%. To achieve robust results, we performed four independent biological repeats in parallel. Next, DNA was extracted from cells and the unique sgRNA was amplified using PCR. The amplicons were then used to generate barcoded libraries for custom next-generation sequencing (NGS) of the amplicons 48), (Fig. S2a).

Our analysis showed that there was no significant batch effect between repeats, and the read count was distributed homogeneously. Importantly, our analysis showed no significant loss of sgRNAs across all samples, indicating that we were able to maintain library complexity, despite the rigorous sorting which may lead to drift in sgRNA population (Fig.S2).

Due to technical limitations and poor lncRNA annotations leading to a high rate of false negatives in CRISPRi screening, we analyzed the enrichment of each sgRNA independently, and the cumulative effect of multiple sgRNAs targeting the same gene.

Out of the 13,548 sgRNAs in the library, a total of 33 were significantly enriched with a threshold of p < 0.01, with 20 from the GFP low and 13 from the high GFP fractions (Fig.1d and S3 and S4). Gene level analysis based on the cumulative score of multiple sgRNAs targeting the same TSS identified five lncRNAs whose KD led to decreased infection (Fig. S2b). One lncRNA, CNPY2-AS1, was ranked high both in the sgRNA analysis and the gene level. For the other lncRNAs, only the cumulative score of sgRNAs generated a significant effect at the gene level.

Next, we used the most differentially represented sgRNAs to validate screening findings. While in most cases we failed to validate screening findings when only one sgRNA was significantly enriched/depleted, two sgRNAs picked from the gene level analysis showed a strong effect on infection (Fig. S4). CRISPR KD of two lncRNAs, CNPY2-AS1 and RP11-314A20.5, led to a reduction in GFP positive cells, as well as in GFP intensity of the infected cells (Fig.1e). This indicates that these lncRNAs affect SARS-CoV-2 cell entry.

Next, we tested whether the observed effect is specific to infection by the SARS-CoV-2 spike protein. To this end, we infected RP11-314A20.5 and CNPY2-AS1 KD cells with the VSV based pseudovirus expressing the vesicular stomatitis virus glycoprotein (VSV-ΔG-G). VSV-∆G-G can infect a wide spectrum of cells by using LDL receptor (LDLR), which is expressed on many cell types, including SNU449^49^. Surprisingly, neither lncRNA affected VSV-∆G-G infection, indicating that RP11-314A20.5 and CNPY2-AS1 specifically affect SARS-CoV-2 infection (Fig.S5). Altogether, our CRISPRi screening identified two novel lncRNAs that showed that pecificity and reproducibility affect cell entry by the SARS-CoV-2 spike protein in an ACE2-dependent manner.

### Cis-regulatory characterization of RP11-314A20.5

Since lncRNAs frequently regulate the transcription of nearby genes through cis-acting mechanisms 50, we first assessed the KD efficiency of CNPY2-AS1 and RP11-314A20.5, as well as the expression of their neighboring protein-coding genes (PCGs). For both lncRNAs, CRISPRi-mediated KD resulted in an approximately 50% reduction in transcript levels compared to control cells. However, their effects on neighboring genes differed markedly (Fig. 2b, and 3b). KD of RP11-314A20.5 led to a two-fold reduction in the expression of its closest neighboring genes, including Chemokine (C-X-C motif) ligand 16 (CXCL16), Mediator Complex Subunit 11 (MED11), and ZMYND15 (Fig.2a and b).

Interestingly, CXCL16, a chemokine with a significant role in the immunopathogenesis of severe COVID-19, displayed elevated levels in patients with severe COVID-19 compared to those with mild symptoms or healthy individuals, and it has been associated with life-threatening immune responses 51,52. The effect of CXCL16 on SARS-CoV-2 outcomes was linked to an elevated immune response, suggesting an independent function of RP11-314A20.5 in viral infection.

RP11-314A20.5 is a primate-specific antisense lncRNA composed of two exons and partially overlapping the second exon of MED11 (Fig. 2a). Perturbation of the RP11-314A20.5 locus altered the expression of neighboring genes, consistent with cis-regulatory activity at this genomic locus, a mechanism described for several lncRNA loci that can operate independently of RNA sequence-specific activity 53-56.

To determine whether MED11 itself contributes to SARS-CoV-2 entry, we performed CRISPR-Cas9 deletion using dual sgRNAs targeting the first exon of MED11. MED11 is an essential component of the Mediator complex, which serves as a master regulator of RNA polymerase II (Pol II) transcriptional activity. Interestingly, several Mediator subunits have been found to play crucial roles in the infection cycles of various viruses, including SARS-CoV-2^57^,^58^, likely through the transcriptional regulation of multiple host genes.

Although the MED11 mutation did not directly target RP11-314A20.5, MED11 KO significantly reduced RP11-314A20.5 expression (Fig. 2c). Conversely, RP11-314A20.5 KD reduced the expression of the neighboring gene CXCL16, whereas MED11 KO had no effect on CXCL16 expression (Fig. 2b, c). These findings indicate that perturbations of RP11-314A20.5 and MED11 have distinct effects on local gene expression. Furthermore, MED11 KO significantly reduced VSVΔG-S infection (Fig. 2d). To confirm MED11's role, rescue experiments demonstrated that ectopic expression of MED11, but not RP11-314A20.5, restored VSVΔG-S infection in MED11 KO cells (Fig. 2d). This suggests that MED11 is a primary limiting factor for viral entry at this locus, whereas RP11-314A20.5 is not sufficient to promote infection when supplied in trans. These observations are consistent with a cis-regulatory role for the RP11-314A20.5 genomic locus (Fig.3d). Together, these results support the presence of local cis-regulatory interactions within the RP11-314A20.5/MED11 locus that affect the expression of neighboring genes, including immune-related factors. Ultimately, this implicates this genomic region.

### CNPY2-AS1 regulates SARS-CoV-2 spike-mediated entry independently of neighboring genes

The second validated lncRNA, CNPY2-AS1, is broadly expressed across tissues and cell types (Fig. S6). It is located on chromosome 12q13.3 and is flanked by three PCGs: CS, CNPY2, and PAN2 (Fig. 3a). CRISPRi-mediated KD of CNPY2-AS1 selectively reduced expression of citrate synthase (CS), which shares a divergent promoter with CNPY2-AS1 (Fig. 3a, and b), whereas CNPY2 and PAN2 expression remained unchanged (Fig. 3b).

Because CRISPRi targeting divergent promoters can inadvertently repress neighboring genes, we addressed the possibility that the observed phenotype resulted from bystander effects. To this end, we developed an orthogonal KO strategy targeting a ~150 bp region encompassing the 3′ splice acceptor site of the final exon 59 (Figure. S7a). This approach disrupts proper splicing of the last exon, leading to transcript destabilization and efficient RNA degradation. Importantly, this strategy substantially reduced CNPY2-AS1 expression without affecting CS, CNPY2, or PAN2 (Fig. 3c). Consistent with CRISPRi results; CNPY2-AS1 KO cells exhibited a pronounced reduction in SARS-CoV-2 spike-mediated infection (Fig. 3d). This phenotype was recapitulated in a lung-derived cell line, indicating that the role of CNPY2-AS1 in viral entry is conserved across cell types (Fig.S8).

Importantly, VSV-ΔG-G mediated infection remained unaffected, confirming that CNPY2-AS1 specifically regulates SARS-CoV-2 spike-dependent entry independently of neighboring genes (Fig. S7b).

### CNPY2-AS1 loss induces an interferon stimulated transcriptional program with PLSCR1 upregulation and reduced ACE2 surface availability

Despite this clear and specific phenotype, the biological function of CNPY2-AS1 remains poorly understood. To gain insight into the molecular pathways underlying this entry restriction, we performed transcriptome profiling of CNPY2-AS1 KO and control cells. Loss of CNPY2-AS1 induced extensive transcriptional remodeling, with 1,370 differentially expressed genes (DEGs) identified (|log₂FC| > 2, adjusted *p* < 0.05) (Fig. S9). Comparison with curated gene sets from Rummagene^60^ revealed a strong similarity to transcriptional changes observed in SARS-CoV-2-infected nasal epithelial cells (61, underscoring the relevance of CNPY2-AS1-dependent pathways to coronavirus infection.

Among the upregulated genes, the ISG PLSCR1 was prominently induced in CNPY2-AS1 KO cells (Fig. 4a; Fig. S10a), a finding that was validated by RT-qPCR (Fig. 4b). PLSCR1 is known to regulate phospholipid redistribution, macromolecule trafficking, and transcription (61. Notably, multiple studies have demonstrated that PLSCR1 inhibits SARS-CoV-2 entry by targeting viral vesicles, thereby preventing membrane fusion and cytosolic RNA release (38,62,63. Furthermore, PLSCR1 was recently shown to specifically downregulate membrane-bound ACE2^38^ with this reported mechanism, our experimental analysis revealed that CNPY2-AS1 KO did not affect total cellular ACE2 levels (Fig. 4c) but led to a significant reduction in surface ACE2, as determined by flow cytometry (Fig. 4d-e).

To functionally validate the contribution of PLSCR1 to the observed antiviral phenotype, we generated PLSCR1 CRISPR KO cells (Fig. S10b). Loss of PLSCR1 significantly increased susceptibility to VSVΔG-S infection (Fig. 4f), confirming that PLSCR1 acts as a key effector of the antiviral response induced by CNPY2-AS1 loss. Collectively, these findings position PLSCR1 within a broader CNPY2-AS1 dependent transcriptional program characterized by robust ISG upregulation.

### Loss of CNPY2-AS1 leads to IRF7 STAT1 activation and inflammatory gene induction

While PLSCR1 emerged as a prominent ISG upregulated upon CNPY2-AS1 loss (Fig. 4a; Fig. S10a), RNA sequencing revealed that this induction occurred as part of a broader interferon stimulated transcriptional program. To define the upstream regulatory mechanisms driving this antiviral response, we performed gene set enrichment analysis of upregulated DEGs. This analysis revealed significant enrichment of innate immune and cytokine signaling pathways (FDR < 0.05) (Fig. 4g), consistent with activation of a type I interferon like program.

Transcription factor enrichment analysis using ChEA3 identified IRF7 as a top-ranked candidate upstream regulator (Fig. S11), consistent with its increased expression in the RNA-seq dataset (Fig. 4a). IRF7 is a central mediator of type I interferon signaling that drives transcription of IFNα/β and a broad spectrum of ISGs. IRF7 has also been implicated inflammatory responses and severe COVID-19 pathology (64. These observations suggest that *CNPY2-AS1* may restrain IRF7-centered innate immune activation. We therefore examined IRF7 abundance and activation by subcellular fractionation using the rapid cell fractionation protocol (REAP) (65, followed by immunoblotting for IRF7 and phosphorylated IRF7 (p-IRF7). Consistent with the RNA-seq results, *CNPY2-AS1* KO cells exhibited increased IRF7 protein abundance compared with control cells (Fig. S12a). Notably, nuclear p-IRF7 was also increased in *CNPY2-AS1* KO cells, consistent with enhanced nuclear accumulation of activated IRF7 (Fig. S12b). In parallel, *CNPY2-AS1* KO cells displayed marked induction of inflammatory transcripts, including *CCL2*, *CXCL8*, *IL1A*, and *SAA1* (Fig. 4a). Together, these findings indicate that loss of *CNPY2-AS1* is associated with enhanced IRF7 activation and broader innate immune and inflammatory signaling.

Because interferon signaling converges STAT-mediated transcriptional responses (64,66, we next examined STAT1 dynamics. Under unstimulated conditions, STAT1 was significantly enriched in the nucleus of *CNPY2-AS1* KO cells compared with control cells (Fig. 5a, e), consistent with enhanced basal or tonic activation. Upon IFN-γ stimulation, KO cells exhibited accelerated and markedly enhanced STAT1 nuclear translocation over the 15-60 min time course relative to controls (Fig. 5b-e). Thus, loss of *CNPY2-AS1* enhances STAT1 activation both basally and following interferon stimulation, consistent with the widespread ISG induction observed in *CNPY2-AS1*-deficient cells. Collectively, the increased abundance and nuclear accumulation of activated IRF7, together with enhanced basal and IFN-γ-induced STAT1 activation, supports a model in which *CNPY2-AS1* negatively regulates interferon-associated innate immune signaling.

### CNPY2-AS1 modulates innate immune signaling via TXNRD1-dependent redox regulation

To investigate the molecular mechanisms through which *CNPY2-AS1* regulates gene expression and cellular phenotypes, we performed RNA pulldown followed by mass spectrometry. Among the proteins identified, TXNRD1 was one of the most enriched candidate interactors (Fig. 6a and Fig. S13a; Supplementary Table 7). TXNRD1 is an essential selenoprotein that maintains thioredoxin in its reduced, active state, thereby contributing to cellular defense against oxidative stress 67. Given the identification of TXNRD1 in the unbiased RNA pulldown-mass spectrometry screen, we next examined whether loss of *CNPY2-AS1* was accompanied by alterations in the thioredoxin redox system. RNA-seq analysis of *CNPY2-AS1* KO cells revealed marked induction of ISGs, together with coordinated dysregulation of pathways involved in cellular redox homeostasis. Among the redox-related transcripts identified by RNA-seq, *TXNIP*, a negative regulator of thioredoxin activity, was upregulated, whereas *TXNRD1* was downregulated. These expression changes were validated by RT-qPCR, and immunoblotting further confirmed reduced TXNRD1 protein abundance (Fig. 6b, S16a). Consistent with altered redox homeostasis, intracellular reactive oxygen species (ROS), measured using the fluorogenic dye DCFDA, were significantly elevated in *CNPY2-AS1* KO cells (Fig. 6c).

Thus, the identification of TXNRD1 by RNA pulldown was supported by independent transcriptomic and phenotypic evidence linking CNPY2-AS1 loss to disruption of the thioredoxin-dependent redox system. Together, these findings suggest that CNPY2-AS1 is associated with the TXNRD1-TXNIP redox axis and that its loss may contribute to impaired thioredoxin-dependent redox homeostasis and increased oxidative stress. To determine whether CNPY2-AS1 and TXNRD1 occupy a common subcellular compartment, we performed cytoplasmic and nuclear fractionation followed by RT-qPCR. CNPY2-AS1 was consistently detected in both nuclear and cytoplasmic fractions across SNU449, HL-60, and U2OS cells, indicating dual subcellular localization (Fig. S13b). The purity of the fractions was confirmed using appropriate nuclear and cytoplasmic compartment specific controls. Immunofluorescence analysis demonstrated that TXNRD1 was predominantly localized in cytoplasm. (Fig. S13c).

These findings are consistent with previous reports describing TXNRD1 as primarily a cytoplasmic or cytosolic protein. Importantly, the presence of CNPY2-AS1 within the cytoplasmic compartment establishes the spatial compatibility required for interaction with TXNRD1, supporting the physiological relevance of the interaction identified by RNA pull-down and mass spectrometry.

In wild-type cells, CNPY2-AS1 may support TXNRD1 function and preserve redox homeostasis, whereas its absence leads to elevated ROS. Consistent with this role, ROS levels measured using the fluorogenic dye DCFDA were significantly elevated in CNPY2-AS1 KO cells (Fig. 6c). Because ROS can activate JAK/STAT signaling, including STAT1 phosphorylation and nuclear translocation 68,69. We next asked whether oxidative stress is sufficient to recapitulate key phenotypes observed upon CNPY2-AS1 loss.

Treatment of WT cells with 150 µM H₂O₂ increased intracellular ROS, promoted STAT1 nuclear localization, and reduced SARS-CoV-2 spike-mediated pseudovirus infection (Fig. 6d, S14).

In parallel, H₂O₂ exposure also increased expression of STAT1, partially recapitulating the transcriptional response observed upon CNPY2-AS1 loss (Fig. S14a).

These results indicate that oxidative stress is sufficient to induce STAT1-associated antiviral signaling and reduce spike-mediated viral entry.

To determine whether TXNRD1 functionally contributes to these phenotypes, we perturbed *TXNRD1* using two independent approaches: esiRNA-mediated silencing and pharmacological inhibition with TRI-1. Both TXNRD1 KD and TXNRD1 inhibition increased intracellular ROS, enhanced STAT1 activation and nuclear localization, and reduced SARS-CoV-2 spike-mediated infection (Fig. 7a-c and Figs. S15, S16). Thus, TXNRD1 loss of function phenocopied key redox, signaling, and viral-entry phenotypes observed following *CNPY2-AS1* loss, further linking TXNRD1-associated redox control to the cellular effects of *CNPY2-AS1* deficiency.

As a complementary approach, we sought to restore redox capacity in *CNPY2-AS1* KO cells. TXNRD1 is a selenoprotein whose functional expression requires SECIS-dependent recoding of an in-frame UGA codon, an intact selenocysteine incorporation machinery, and sufficient selenium availability, making conventional plasmid-based overexpression technically challenging and potentially unreliable 70. We therefore used two complementary redox-rescue strategies. First, we activated the NRF2 pathway using dimethyl fumarate (DMF), which induces antioxidant gene-expression programs, including components of the thioredoxin system 71-73. Second, we supplemented cells with sodium selenite to increase selenium availability for selenoprotein synthesis, including TXNRD1 74,75.

Both DMF treatment and sodium selenite supplementation partially restored redox homeostasis in *CNPY2-AS1* KO cells, reducing intracellular ROS levels, attenuating STAT1 activation and nuclear localization, and partially restoring spike-mediated viral infection (Fig. 7d-f and Fig. S17). These effects were opposite to those observed following TXNRD1 loss of function and indicate that restoration of cellular redox capacity can partially reverse the signaling and viral-entry phenotypes associated with *CNPY2-AS1* deficiency.

Together, the TXNRD1 perturbation and redox-rescue experiments support a functional relationship between *CNPY2-AS1*, TXNRD1-associated redox control, and STAT1 activation.

We next considered whether the observed downregulation of *TXNRD1* could reflect a local *cis*-regulatory effect of the *CNPY2-AS1* genomic locus. However, *CNPY2-AS1* and *TXNRD1* are separated by approximately 48 Mb on chromosome 12, making conventional local *cis*-regulation unlikely. Instead, the identification of TXNRD1 as a candidate *CNPY2-AS1*-interacting protein, together with the convergent effects of *CNPY2-AS1* deficiency and TXNRD1 loss of function and the partial reversal of these phenotypes by redox rescue, supports a model in which *CNPY2-AS1* influences cellular redox homeostasis through a *trans*-acting mechanism linked to TXNRD1-associated redox control.

Collectively, these findings support a model in which *CNPY2-AS1* contributes to cellular redox homeostasis through a TXNRD1-linked pathway, thereby restraining ROS-associated STAT1 activation. Loss of *CNPY2-AS1* disrupts this balance, promoting ROS accumulation and enhancing STAT1 signaling, accompanied by increased ISG expression, including *PLSCR1*, and reduced SARS-CoV-2 spike-mediated viral infection (Fig. 8). Consistent with the potential clinical relevance of this model, low *CNPY2-AS1* expression was associated with severe COVID-19 and poorer clinical outcomes (Fig. S18).

## Discussion

LncRNAs have emerged as important regulators of gene expression and cellular homeostasis, yet their contributions to host virus interactions remain incompletely defined. Although several lncRNAs have been implicated in immune regulation, systematic functional interrogation of the non-coding genome in the context of viral infection remains limited. A more comprehensive understanding of lncRNA-mediated regulatory mechanisms is therefore important for defining host determinants of viral susceptibility and immune dysregulation.

Here, we employed a genome-scale CRISPRi-based screening approach to identify lncRNAs that modulate SARS-CoV-2 cell entry. This unbiased strategy enabled the identification of functional non-coding regulators that are not readily predicted from genomic annotation or expression profiling alone, underscoring the utility of pooled loss-of-function screens for defining lncRNA function in complex biological processes.

Our findings reveal distinct regulatory paradigms through which lncRNA loci can influence host responses to viral infection. The *RP11-314A20.5/MED11* locus exemplifies a local cis-regulatory mechanism in which perturbation of the lncRNA-associated genomic region modulates neighboring gene expression, linking this locus to pathways involved in transcriptional regulation and immune signaling.

These observations are consistent with previous studies demonstrating that lncRNA loci can regulate neighboring genes through local cis-acting mechanisms. Our findings extend this paradigm to host regulatory pathways associated with viral entry and inflammatory responses. In contrast, our findings support *CNPY2-AS1* as a trans-acting regulator linking cellular redox homeostasis to innate immune signaling. Rather than acting primarily through adjacent coding genes, *CNPY2-AS1* appears to influence a regulatory network involving TXNRD1-associated redox control and STAT1 activation. The identification of TXNRD1 as a candidate *CNPY2-AS1*-interacting protein, together with the convergent effects of *CNPY2-AS1* deficiency and TXNRD1 loss of function and the partial reversal of these phenotypes by redox rescue, supports a model in which *CNPY2-AS1* contributes to cellular redox regulation through a trans-acting mechanism. This mode of regulation highlights a role for lncRNAs in coordinating cellular metabolic state with antiviral signaling pathways.

Redox-dependent modulation of STAT1 signaling has been documented in the context of interferon responses and inflammatory amplification. Our identification of *CNPY2-AS1* as a non-coding regulator associated with this axis provides insight into how redox homeostasis and innate immune signaling may be coordinated. Loss of *CNPY2-AS1* increased intracellular ROS, enhanced STAT1 activation and nuclear localization, and induced broad expression of interferon-stimulated genes. Importantly, experimentally induced oxidative stress reproduced key signaling and viral-entry phenotypes associated with *CNPY2-AS1* loss, whereas TXNRD1 knockdown and pharmacological inhibition produced similar effects. Conversely, restoration of cellular redox capacity partially reduced ROS and STAT1 activation while restoring spike-mediated viral infection. Together, these observations support a model in which *CNPY2-AS1*-associated redox control restrains excessive ROS-associated STAT1 activation and thereby modulates interferon-associated immune signaling.

These findings may also have clinical relevance. Excessive interferon-associated signaling and inflammatory cytokine production have been linked to severe COVID-19. In our study, low *CNPY2-AS1* expression was associated with severe disease and poorer clinical outcomes, consistent with the possibility that loss of this regulatory mechanism contributes to dysregulated immune activation rather than an appropriately controlled antiviral response. This relationship is particularly notable because *CNPY2-AS1* deficiency reduced SARS-CoV-2 spike-mediated infection at the cellular level while enhancing ROS accumulation, STAT1 signaling, and ISG expression. Thus, increased antiviral restriction at the level of viral entry may coexist with an inflammatory state that is potentially detrimental to the host, illustrating the importance of balancing antiviral defense with control of immune activation.

Beyond their mechanistic implications, these findings suggest potential avenues for therapeutic investigation. LncRNAs such as *CNPY2-AS1* may provide opportunities to modulate regulatory networks without directly targeting essential protein-coding genes. However, our results also highlight the need to consider the balance between antiviral restriction and inflammatory activation. Perturbations that increase oxidative stress may restrict viral entry while simultaneously enhancing innate immune signaling, whereas interventions that restore redox homeostasis may attenuate inflammatory signaling but partially restore susceptibility to viral entry. Components of the *CNPY2-AS1-*redox axis should therefore be considered potential targets for modulating this balance rather than for independently suppressing both viral infection and inflammation.

In summary, this study identifies lncRNA loci as modulators of host responses to viral infection and reveals mechanistically distinct modes of regulation. The *RP11-314A20.5/MED11* locus illustrates local cis-regulatory control of neighboring gene expression, whereas *CNPY2-AS1* supports a trans-acting mechanism linking TXNRD1-associated redox homeostasis to innate immune signaling. Together, these findings expand current models of host-virus interactions by incorporating non-coding RNA-mediated regulation of viral entry, cellular redox balance, and inflammatory signaling.

## Supplementary Material

Supplementary figures.

Supplementary tables.

## Figures and Tables

**Figure 1:**
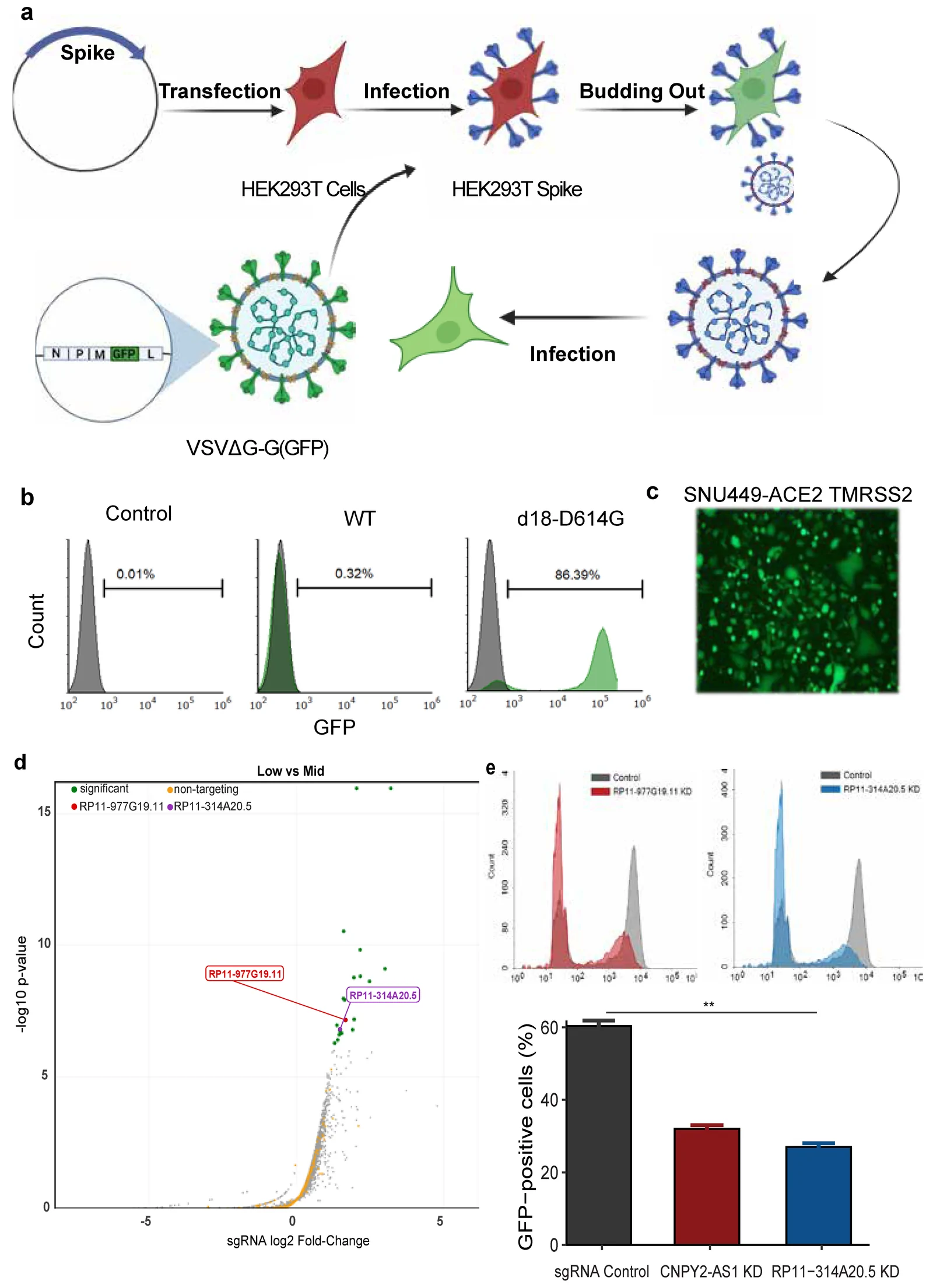
** Generation and characterization of VSV-S pseudotyped viruses for assessing SARS-CoV-2 spike-mediated infection. a.** Schematic representation of VSVΔG pseudotyped particle production, illustrating the incorporation of the SARS-CoV-2 spike protein on the viral surface. The pseudoviruses encode a GFP reporter gene, allowing visualization of infection efficiency. **b.** Flow cytometry analysis of GFP signal intensity in HEK293T cells expressing ACE2-TMPRSS2 infected with VSV-S pseudotyped viruses carrying different SARS-CoV-2 spike protein variants, including WT, Δ18, and Δ18-D614G. Wild-type cells without ACE2 expression serve as a control. **c.** Representative fluorescence microscopy images showing GFP intensity in SNU449-ACE2-TMPRSS2 cells. GFP expression serves as a readout for infection efficiency. **d**. Volcano plot of sgRNA enrichment in the Low population relative to the Mid population following CRISPR screening. Each point represents an individual sgRNA, plotted according to its log2 fold-change (x-axis) and -log10 *P* value (y-axis). Significant sgRNAs are shown in green, non-targeting sgRNAs in orange, and non-significant sgRNAs in gray. Significant sgRNAs targeting RP11-977G19.11 and RP11-314A20.5 are highlighted in red and purple, respectively. PinAPL-Py analysis identified 20 significantly enriched genes in the Low versus Mid comparison. **e.** Validation of top enriched sgRNAs from the CRISPRi screen. SNU449-ACE2 cells expressing sgRNAs targeting selected candidate lncRNAs were infected with VSV-∆G pseudotyped with the Δ18-D614G spike variant. Top: Flow cytometry analysis based on GFP expression. Bottom: Quantification from n > 3 independent experiments. Statistical analysis compares lncRNA-targeting sgRNAs to a non-targeting control sgRNA using a two-tailed Student's t-test (*p < 0.05, ** p< 0.01, *** p< 0.001). Error bars represent mean ± SD.

**Figure 2:**
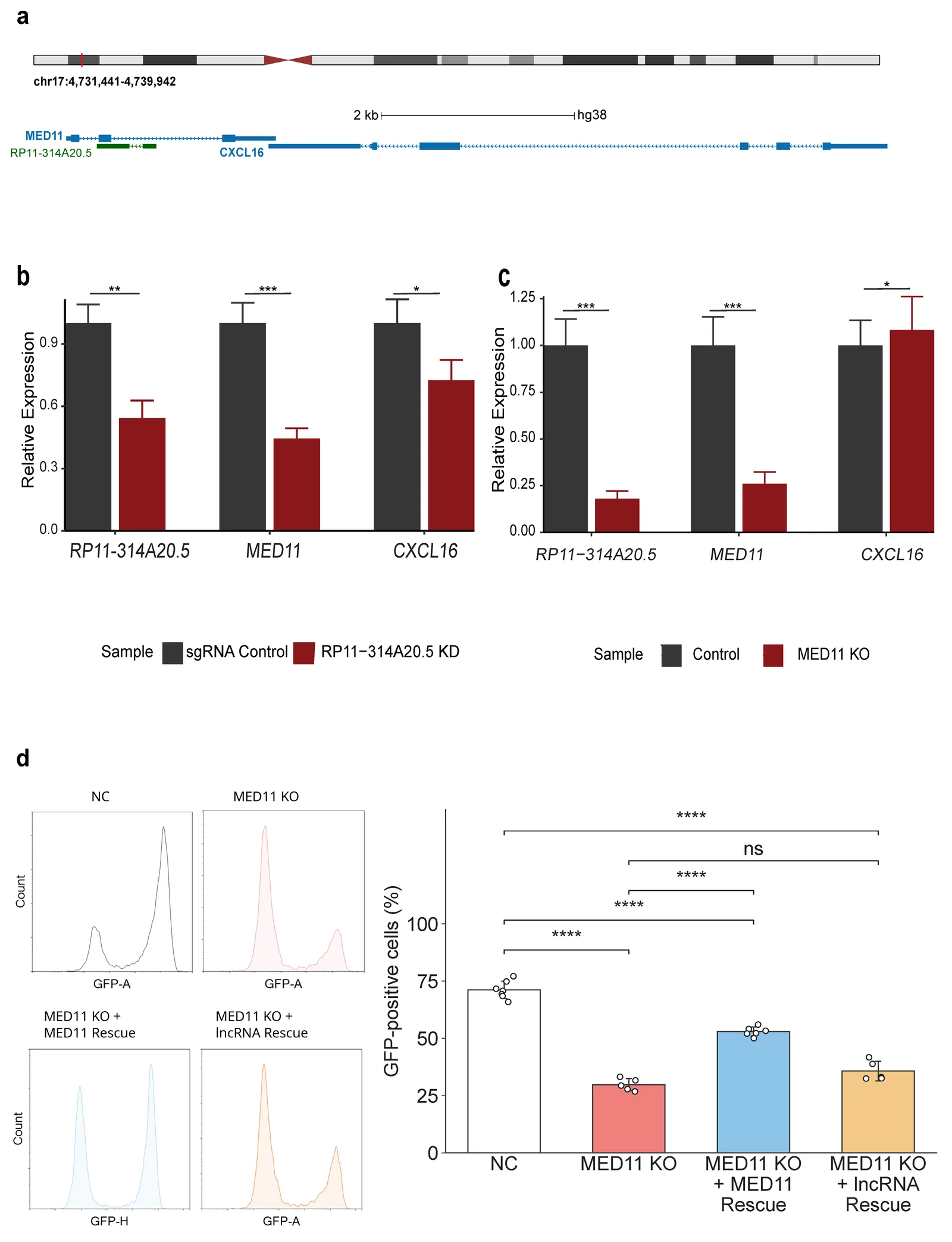
** Role of RP11-314A20.5 and MED11 in gene regulation and VSV-S infection. a**. Schematic representation of lncRNA RP11-314A20.5 and its neighboring genes, MED11 and CXCL16, adapted from the UCSC Genome Browser (GENCODE V47). **b** Relative gene expression levels following RP11-314A20.5 CRISPRi KD and its effect on neighboring genes, measured by RT-qPCR. Expression in sgRNA-targeted RP11-314A20.5 cells was compared to a non-targeting sgRNA control (n > 3). **c.** Expression of RP11-314A20.5 and neighboring genes in MED11 KO cells. Relative expression levels of RP11-314A20.5, MED11, and CXCL16 were measured in MED11 KO cells and compared to a non-targeting sgRNA control (n > 3). **d**. Effect of MED11 CRISPR KO on VSVΔG-S infection. Left: Representative flow cytometry histograms showing GFP intensity, used as a proxy for viral infection, in SNU449-ACE2 cells following MED11 knockout and rescue experiments. Conditions include control cells (NC), MED11 KO cells, MED11 KO cells rescued by ectopic expression of MED11 ORF (MED11 KO + MED11 Rescue), and MED11 KO cells transfected with the lncRNA RP11-314A20.5 transcript (MED11 KO + RP11-314A20.5 Rescue). Right: Quantification of GFP-positive cells (%) across conditions from n ≥ 3 independent biological replicates. Data are presented as mean ± SD. Statistical significance was determined using a two-tailed t-test, where *p < 0.05, **p < 0.01, ***p < 0.001, ****p < 0.0001, and ns = not significant.

**Figure 3:**
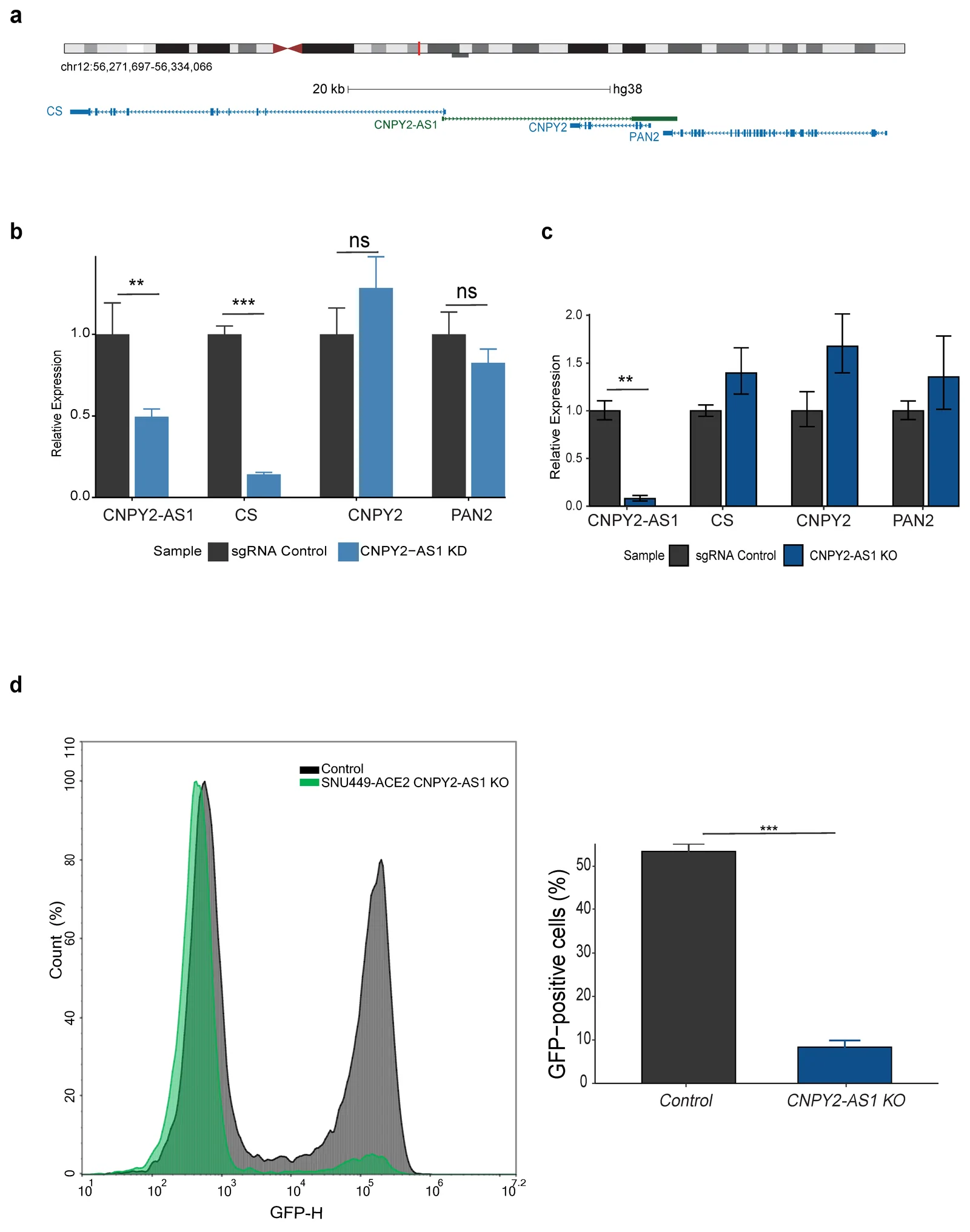
** Functional characterization of CNPY2-AS1 in gene regulation and VSV-S infection. a.** Genomic map of CNPY2-AS1 and its neighboring genes (CS, CNPY2-AS1, CNPY2, and PAN2) on chromosome 12 (q11.1), adapted from the UCSC Genome Browser (GENCODE V47). **b.** Expression levels of neighboring genes (CS, CNPY2, PAN2) in CNPY2-AS1 KD cells compared to a non-targeting control sgRNA (n = 3). Statistical significance was determined using a two-tailed t-test (*p* < 0.05, p < 0.01, *p < 0.001). **c** Expression analysis of neighboring genes (CS, CNPY2, PAN2) following CNPY2-AS1 KO (n = 3). Statistical significance was determined using a two-tailed t-test (* < 0.05, ** < 0.01, *** < 0.001, ns = not significant). Error bars represent mean ± SD d. Quantification of GFP expression following VSVΔG-S infection in CNPY2-AS1 KO and non-targeting control cells. Left: Representative flow cytometry analysis. Right: Quantification from n = 3 independent experiments. Statistical significance was determined using a two-tailed t-test (* < 0.05, ** < 0.01, *** < 0.001, ns = not significant). Error bars represent the mean ± SD.

**Figure 4:**
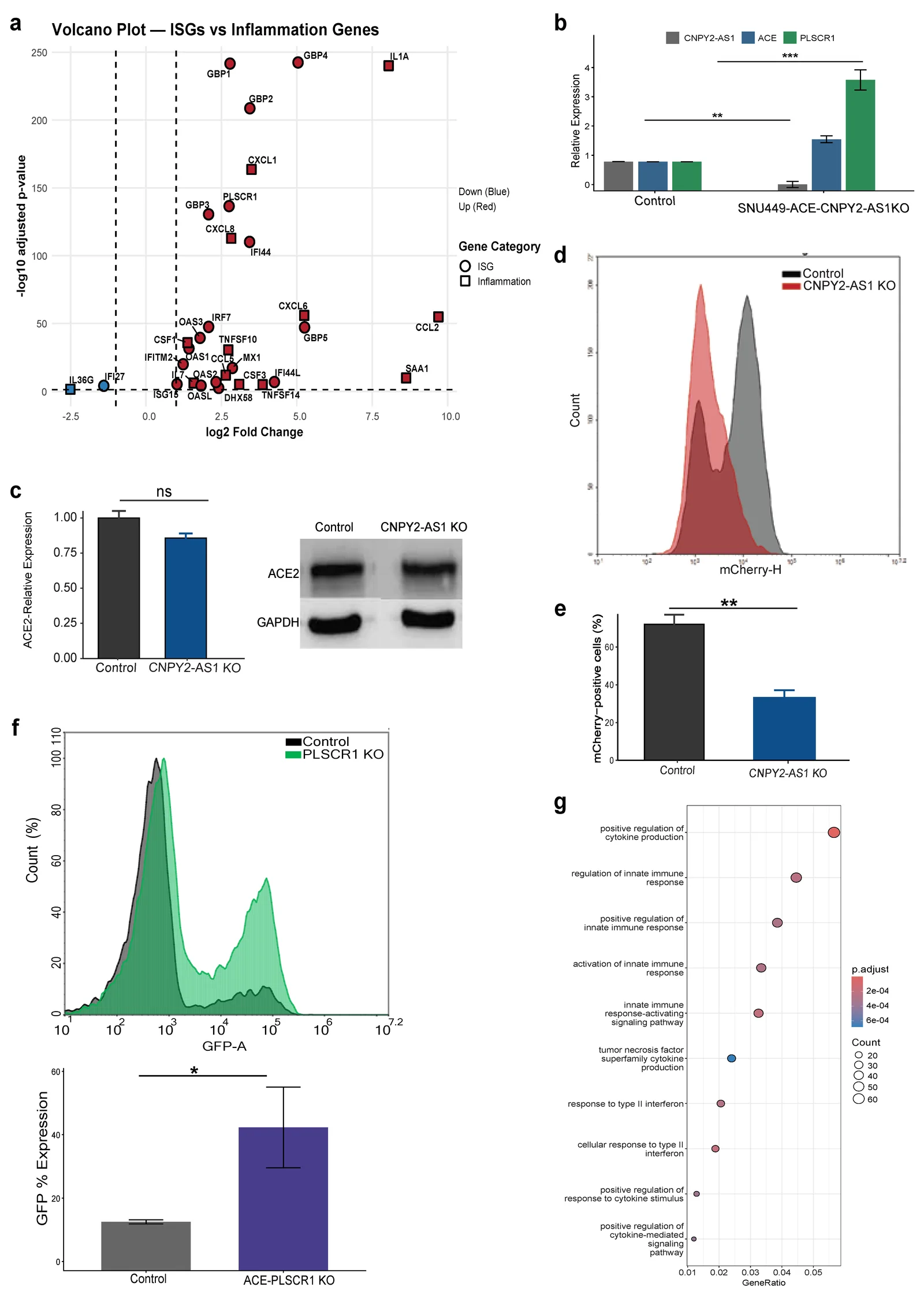
** Loss of CNPY2-AS1 induces an interferon-stimulated transcriptional program associated with PLSCR1 upregulation and reduced ACE2 surface availability. a.** Transcriptomic profiling of CNPY2-AS1 KO and control cells. Volcano plot showing differentially expressed immune genes identified by RNA sequencing. ISGs are highlighted with circles, and inflammatory genes with squares. Upregulated genes are shown in red and downregulated genes in blue. Differentially expressed genes were defined as |log₂ fold change| > 2 with an adjusted p value < 0.05. **b.** Quantitative PCR validation of RNA-seq results. Relative mRNA expression of PLSCR1, ACE2, and CNPY2-AS1 in control and CNPY2-AS1 KO cells. PLSCR1 expression was significantly increased in CNPY2-AS1 KO cells. Expression levels were normalized to housekeeping genes and are shown relative to control cells. Data represent mean ± SD from n > 3 independent experiments. Statistical significance was determined using a two-tailed Student's t-test (P < 0.05, *P < 0.01, **P < 0.001; ns, not significant). **c.** Total cellular ACE2 protein levels in control and CNPY2-AS1 KO cells. Right, representative immunoblot; left, quantification from n = 3 independent experiments. Statistical significance was determined using a two-tailed Student's t-test (P < 0.05, *P < 0.01, **P < 0.001; ns, not significant). Error bars represent mean ± SD. d**.** Representative flow cytometry plots showing surface ACE2 expression in control and CNPY2-AS1 KO cells. **e.** Quantification of ACE2 surface expression from n = 3 independent experiments. Statistical significance was determined using a two-tailed Student's t-test (P < 0.05, *P < 0.01, **P < 0.001; ns, not significant). Error bars represent mean ± SD. **f**. Loss of PLSCR1 increases susceptibility to viral infection. Representative flow cytometry plots (top) showing infection levels in control and PLSCR1 KO cells following VSVΔG-S infection. The percentage of infected cells is shown below, summarizing data from n = 3 independent experiments. Data are presented as mean ± SD; statistical significance was determined using a two-tailed Student's t-test (P < 0.05, *P < 0.01, **P < 0.001; ns, not significant). **g.** Bubble plot showing Gene Ontology (GO) pathway enrichment analysis of upregulated differentially expressed genes (DEGs) in CNPY2-AS1 KO cells.

**Figure 5:**
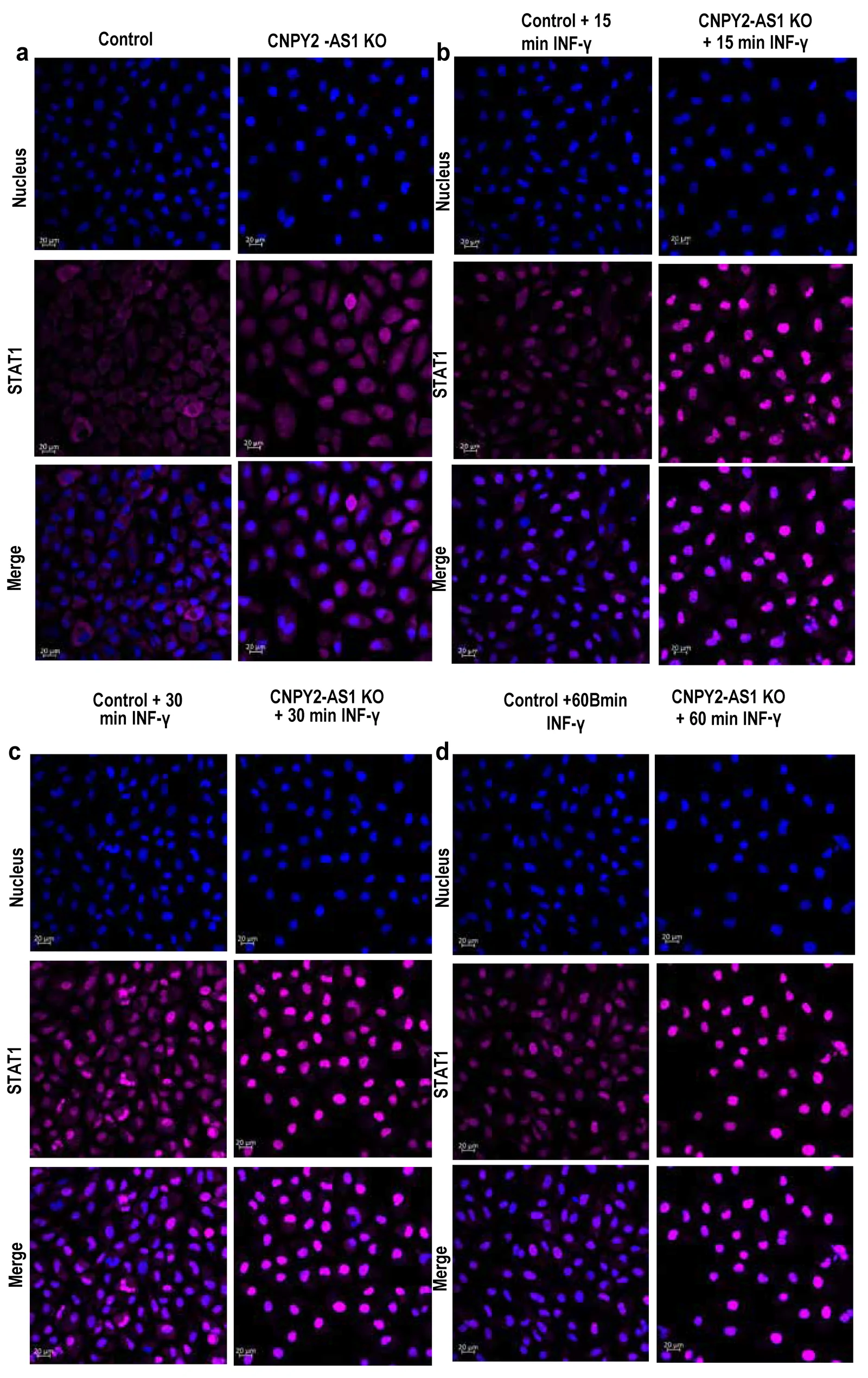

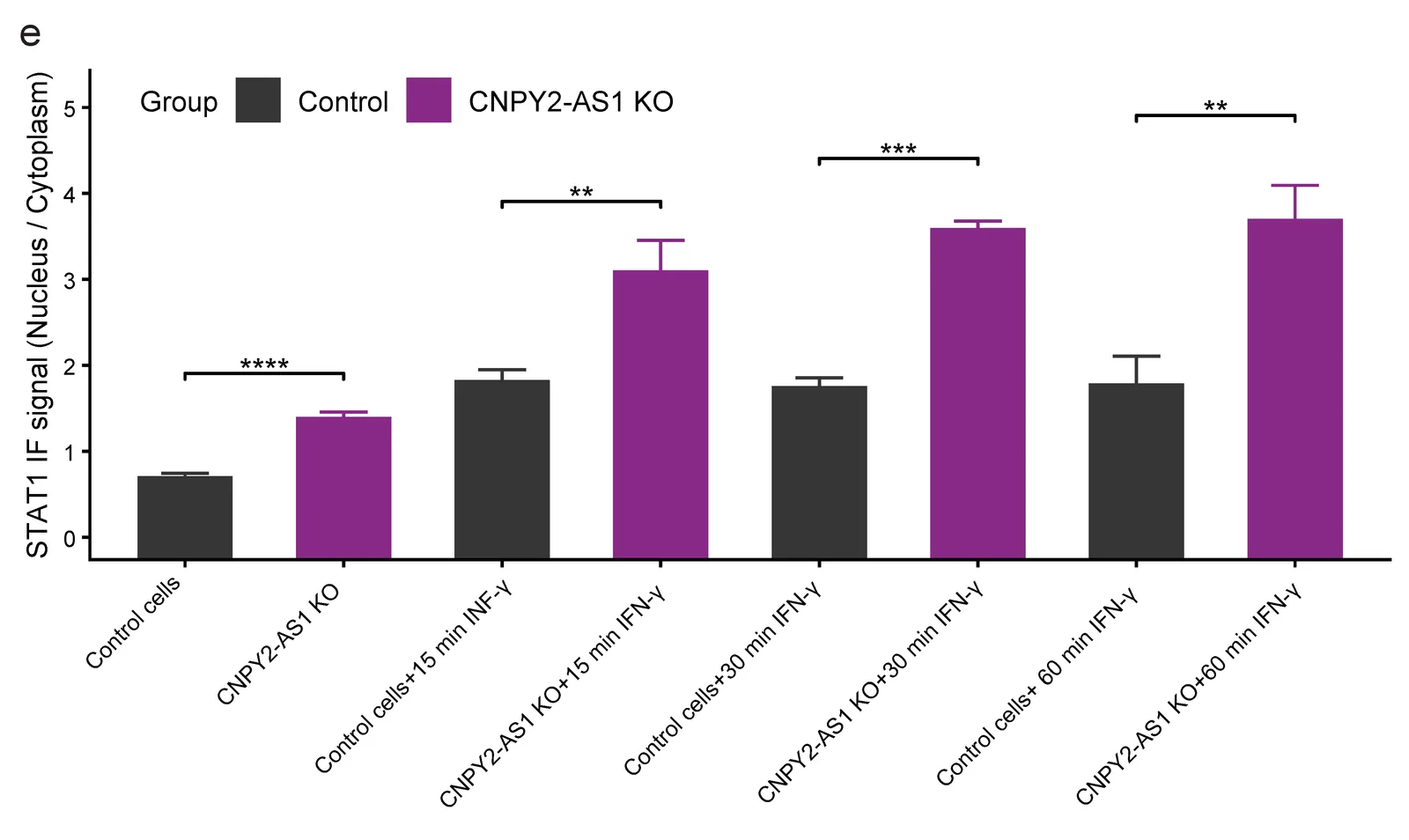
** Loss of CNPY2-AS1 enhances basal and interferon-induced STAT1 nuclear localization. a.** IF staining of STAT1 in control and CNPY2-AS1 KO cells under unstimulated conditions. Nuclei are stained with DAPI (blue), and STAT1 is shown in pink. **b-d.** IF analysis of STAT1 localization in control and CNPY2-AS1 KO cells following IFNγ stimulation for 15 min (b), 30 min (c), and 60 min (d). Representative images are shown. **e.** Quantification of STAT1 nuclear-to-cytoplasmic fluorescence intensity ratios in control and CNPY2-AS1 KO cells under basal conditions and following IFNγ stimulation. Data represent mean ± SD from n >3 independent experiments. Statistical significance was determined using a two-tailed Student's t-test (P < 0.05, *P < 0.01, **P < 0.001; ns, not significant).

**Figure 6:**
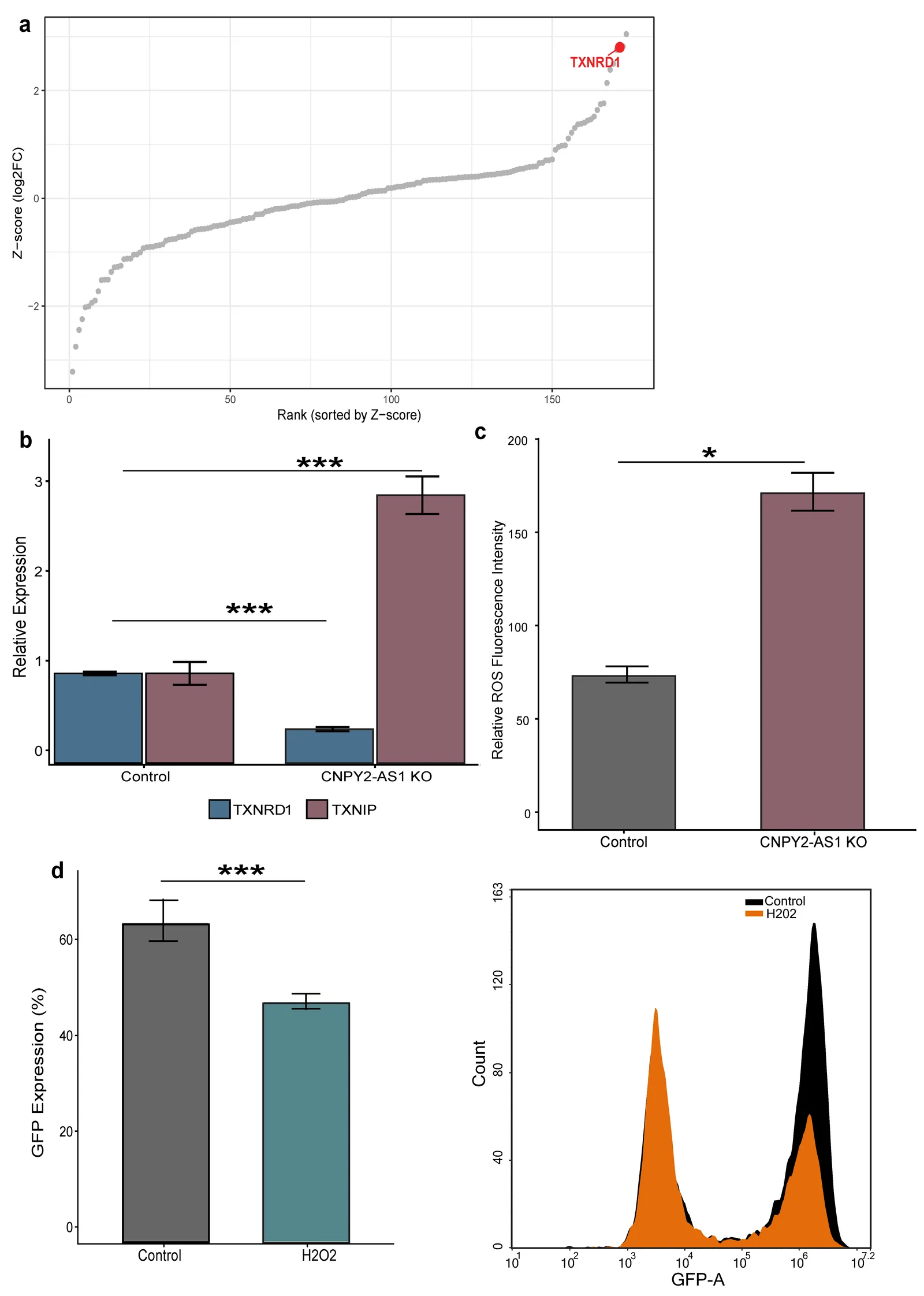
** CNPY2-AS1 interacts with TXNRD1 to regulate redox homeostasis and downstream interferon signaling. a.** RNA pulldown followed by mass spectrometry identifies TXNRD1 as a prominent CNPY2-AS1 interacting protein. **b.** Relative expression levels of TXNRD1 and TXNIP were measured in control and CNPY2-AS1 KO cells as validation of RNA-seq results. Bars represent mean ± SD from three technical replicates. Statistical significance is indicated by asterisks: *P < 0.05, **P < 0.01, ***P < 0.001, ****P < 0.0001. **c.** Measurement of ROS levels using the fluorogenic dye DCFDA in control and CNPY2-AS1 KO cells. Bar plot quantification of DCFDA fluorescence intensity. Data represent mean ± SD from n = 3 independent experiments. Statistical significance was determined using a two-tailed Student's t-test (*p < 0.05, **p < 0.01, ***p < 0.001; ns, not significant). **d**.H2O2 treatment reduces viral infection similar to CNPY2-AS1 KO. Left: Quantification of GFP-positive infected cells in control and H2O2-treated cells. Right: Representative flow cytometry histogram showing GFP expression. Viral infection was measured by flow cytometry based on GFP expression. Data are shown from n ≥ 3 biological replicates and presented as mean ± SD. *P < 0.05, **P < 0.01, ***P < 0.001, ****P < 0.0001.

**Figure 7:**
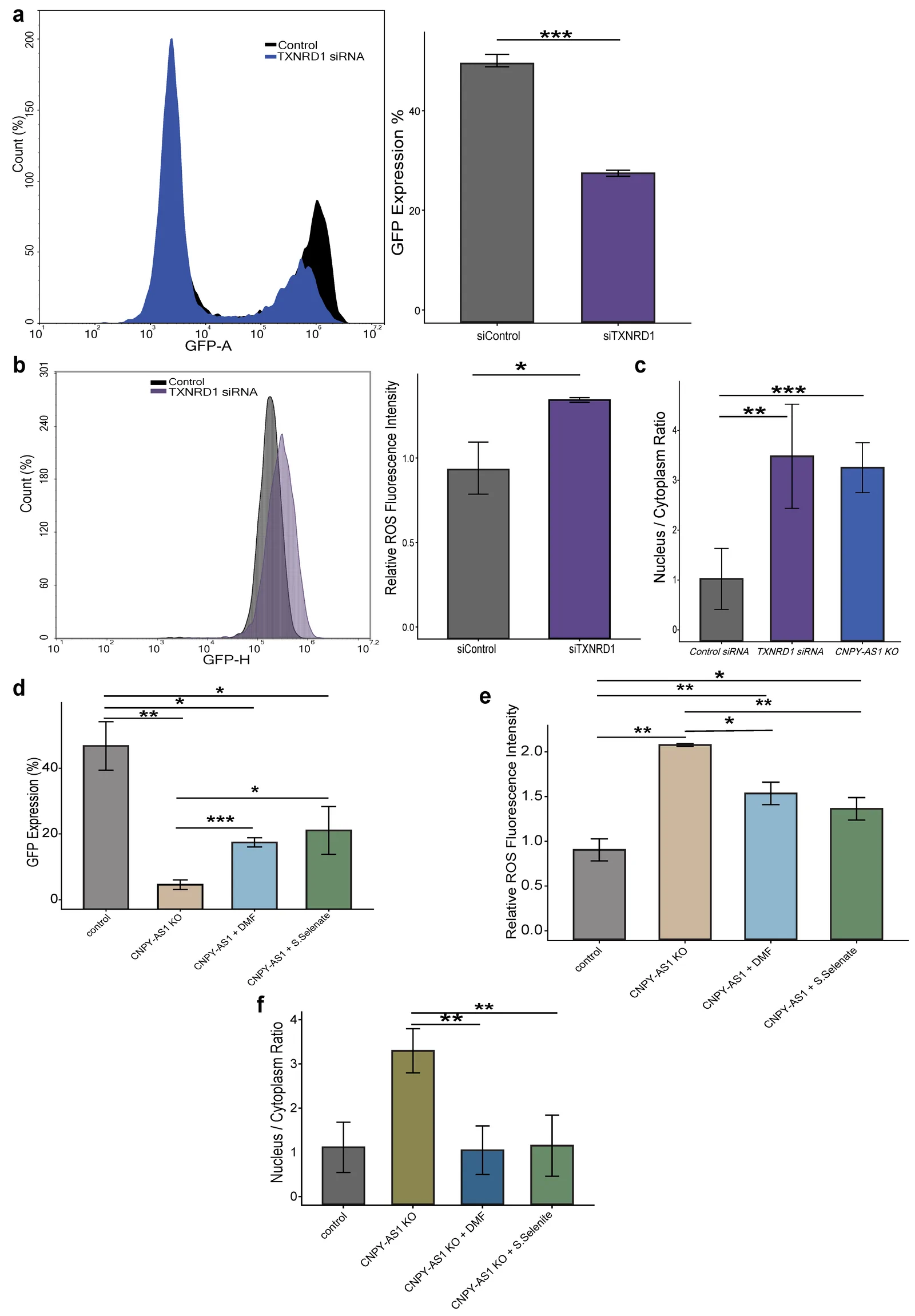
** TXNRD1 mediates CNPY2-AS1-dependent regulation of viral infection, ROS levels, and STAT1 localization. a**. Left: Representative flow cytometry histogram right: quantification of GFP-positive infected cells in siControl and siTXNRD1-transfected cells. Data are shown from n ≥ 3 independent experiments and presented as mean ± SD. *P < 0.05, **P < 0.01, ***P < 0.001, ****P < 0.0001. **b**. Left: Representative flow cytometry histogram. Right: quantification of intracellular ROS levels in siControl and siTXNRD1-transfected cells, measured by DCFDA fluorescence. Data are shown from n ≥ 3 independent experiments and presented as mean ± SD. *P < 0.05, **P < 0.01, ***P < 0.001, ****P < 0.0001. **c**. Quantification of the STAT1 nuclear/cytoplasmic ratio in control siRNA, siTXNRD1-transfected, and CNPY2-AS1 KO cells. Quantification was performed from n ≥ 3 independent experiments, and data are presented as mean ± SD. *P < 0.05, **P < 0.01, ***P < 0.001, ****P < 0.0001. **d**. Quantification of GFP-positive infected cells in control, CNPY2-AS1 KO, CNPY2-AS1 KO + DMF, and CNPY2-AS1 KO + sodium selenite cells, measured by flow cytometry. Data are shown from n ≥ 3 independent experiments and presented as mean ± SD. *P < 0.05, **P < 0.01, ***P < 0.001, ****P < 0.0001. **e**. Quantification of intracellular ROS levels in control, CNPY2-AS1 KO, CNPY2-AS1 KO + DMF, and CNPY2-AS1 KO + sodium selenite cells, measured by DCFDA fluorescence. Data are shown from n ≥ 3 independent experiments and presented as mean ± SD. *P < 0.05, **P < 0.01, ***P < 0.001, ****P < 0.0001. **f**. Quantification of the STAT1 nuclear/cytoplasmic ratio in control, CNPY2-AS1 KO, CNPY2-AS1 KO + DMF, and CNPY2-AS1 KO + sodium selenite cells. Quantification was performed from n ≥ 3 independent experiments, and data are presented as mean ± SD. *P < 0.05, **P < 0.01, ***P < 0.001, ****P < 0.0001.

**Figure 8:**
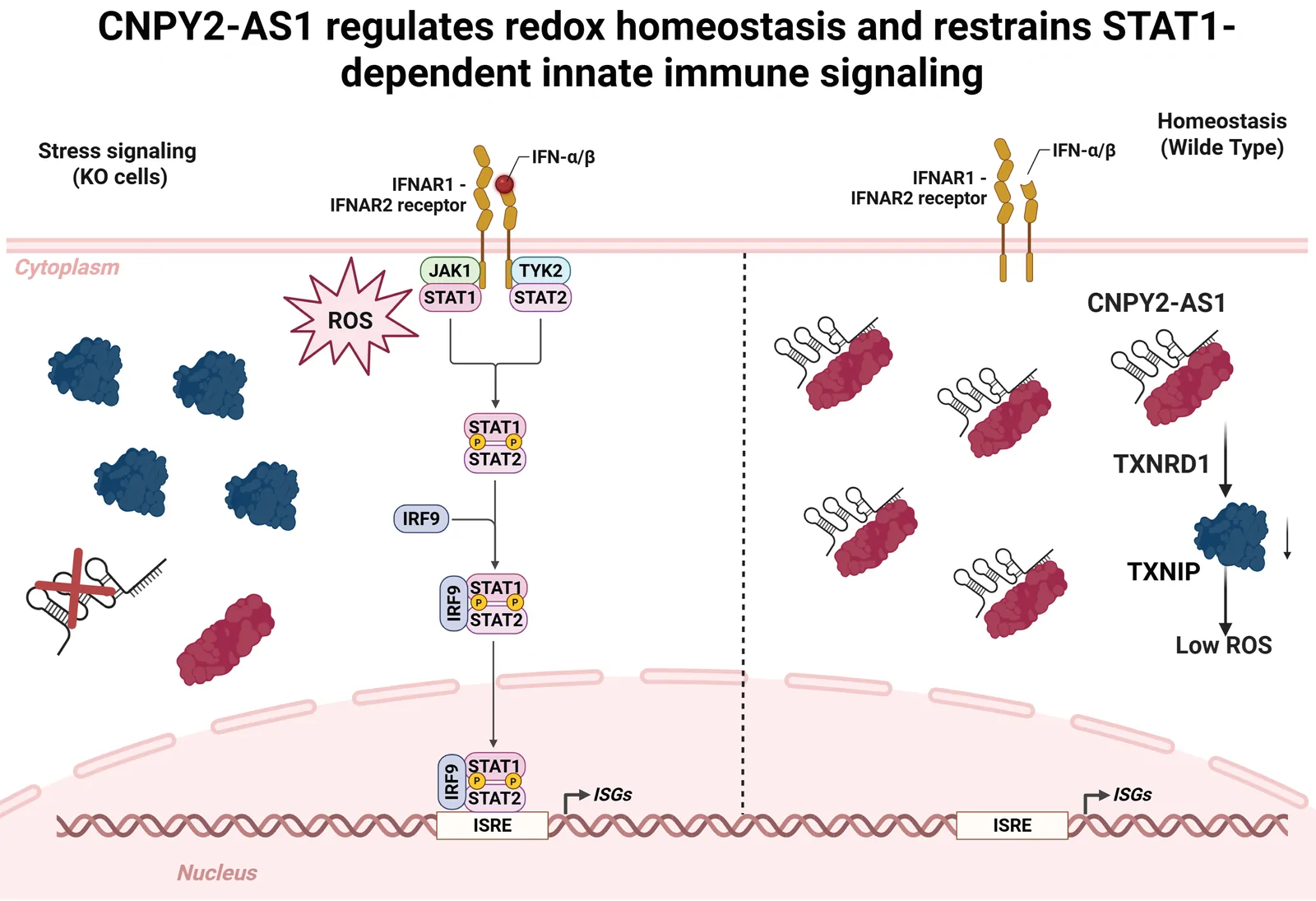
** Proposed model for CNPY2-AS1 dependent regulation of redox balance and STAT1 driven innate immune signaling.** Schematic illustration of the proposed mechanism by which CNPY2-AS1 regulates innate immune signaling and ISG expression. Right: In cells expressing high levels of CNPY2-AS1, the lncRNA associates with TXNRD1, supporting thioredoxin-dependent redox homeostasis and limiting oxidative stress. Under these conditions, STAT1 activation and nuclear accumulation are restrained, resulting in controlled induction of ISGs and inflammatory genes. Left: In CNPY2-AS1 depleted cells, disruption of the TXNRD1-TXNIP axis leads to elevated ROS levels, which favor enhanced JAK STAT signaling and increased STAT1 nuclear localization. This results in amplified ISG transcription and heightened inflammatory cytokine production, collectively contributing to an antiviral but pro-inflammatory cellular state.

## Abbreviations

lncRNA: long non-coding RNA
COVID-19: coronavirus disease 2019
SARS-CoV-2: severe acute respiratory syndrome coronavirus 2
ACE2: angiotensin-converting enzyme 2
TMPRSS2: transmembrane protease serine 2
CRISPRi: clustered regularly interspaced short palindromic repeats interference
TXNRD1: thioredoxin reductase 1
ROS: reactive oxygen species
ISG: interferon-stimulated gene
FBS: fetal bovine serum
Pen-Strep: penicillin-streptomycin
RPMI: Roswell Park Memorial Institute medium
DMEM: Dulbecco's modified Eagle's medium
CO2: carbon dioxide
FACS: fluorescence-activated cell sorting
gDNA: genomic DNA
cDNA: complementary DNA
GFP: green fluorescent protein
PCA: principal component analysis
PI: propidium iodide
LV-PVDF: low-voltage polyvinylidene difluoride
ECL: enhanced chemiluminescence
VSV: vesicular stomatitis virus
VSV-ΔG-G: vesicular stomatitis virus lacking glycoprotein G and pseudotyped with G protein
G: glycoprotein
KD: knockdown
KO: knockout
MOI: multiplicity of infection
sgRNA: single-guide RNA
NGS: next-generation sequencing
TSS: transcription start site
LDLR: low-density lipoprotein receptor
PCG: protein-coding gene
CXCL16: C-X-C motif chemokine ligand 16
MED11: mediator complex subunit 11
DEG: differentially expressed gene
IRF7: interferon regulatory factor 7
TXNIP: thioredoxin-interacting protein
H2O2: hydrogen peroxide
CPM: counts per million
FDR: false discovery rate
LC-MS/MS: liquid chromatography-tandem mass spectrometry
BSL-2: biosafety level 2
WT: wild type
Pol II: RNA polymerase II
DMF: dimethyl fumarate
IF: Immunofluorescence
QC: quality control
WB: Western Blot
CS: citrate synthase
