# Supplementary material for: Functional Genomic Screening Identifies lncRNA CNPY2-AS1 as a Regulator of Redox Metabolism and Antiviral Immunity
Source: Int J Biol Sci. 2026 Sep 3;22(14):7760–81. doi: 10.7150/ijbs.132728 (PMC13618004; doi:10.7150/ijbs.132728)

Supplementary Fig.1. Gene expression, pseudovirus infection, and CRISPRi characterization in HEK293T and SNU449 cells. **a.** Flow cytometry analysis of GFP signal intensity in HEK293T cells expressing ACE2-TMPRSS2 infected with VSV-S pseudotyped viruses carrying different SARS-CoV-2 spike protein variants, including WT, Δ18, and Δ18-D614G. Wild-type cells without ACE2 expression serve as a control. **b.** Representative fluorescence microscopy images showing GFP intensity in HEK293T-ACE2-TMPRSS2. GFP expression serves as a readout for infection efficiency. **c**. efficiency in HEK293T and SNU449 cells. Relative mRNA expression levels of SEL1L, ST3GAL4, and SYVN1 in HEK293T (left) and SNU449 (right) cells, as determined by quantitative PCR. Expression values were normalized to housekeeping genes. Data are shown as mean ± SD from independent replicates. **d**. Flow cytometry analysis of GFP-positive cells following infection of HEK293T and SNU449 cells with VSV-GFP pseudovirus. Histograms show GFP fluorescence intensity, and the percentage of GFP-positive cells is indicated. **e.** Generation of SNU449 cells expressing CRISPRi. SNU449 cells were transduced with dCas9-BFP-KRAB or dCas9-mCherry-KRAB-ZIM3 constructs. Successfully transduced cells were enriched using FACS based on BFP or mCherry fluorescence expression**. f.** Relative RNA expression of targeted genes in SNU449-KRAB and SNU449-KRAB-ZIM3 cells. Gene expression levels were quantified using RT-qPCR and normalized to a non-targeting sgRNA control. Error bars represent mean ± standard deviation (SD).

Supplementary Fig 2. CRISPRi screen workflow and sequencing quality control.

a. Schematic representation of the CRISPRi pooled screen. A CRISPRi non-coding library (CRiNCL) (46) targeting 1,329 genes with 10 sgRNAs per TSS, along with 258 non-targeting sgRNAs, was introduced into SNU449-KRAB-ZIM3 cells expressing hACE2. Cells were then infected with VSV-ΔG-Spike encoding GFP and sorted into high and low GFP-intensity populations. The schematic illustrates the experimental workflow, including screening, sorting, and gene identification associated with altered infection dynamics. **b. I**dentification of candidate genes from the CRISPRi screen. The $\log_2$ fold change of the 10 sgRNAs targeting the top five-ranked TSS is shown. Each line represents an individual sgRNA, with enriched sgRNAs indicated in red and depleted sgRNAs in blue, based on MAGeCKFlute screen analysis. **c.** Per-base sequence quality plot generated by FastQC. The x-axis indicates base position along the read, and the y-axis represents Phred quality scores. **d**. Mapping quality distribution of sequencing reads aligned to the sgRNA reference library using Bowtie2. High scores indicate uniquely aligned reads, whereas low scores indicate multi mapped or unaligned reads. Data shown are from a representative control sample (replicate 1). **e**. Box plot showing the distribution of $\log_2$ normalized sgRNA read counts across experimental conditions. Conditions include GFP-high (H), GFP-low (L), and control (C) populations. Four biological replicates are shown for each condition.

Supplementary Fig 3. sgRNA enrichment analysis from CRISPRi screening. a-c. Distribution of significantly enriched sgRNAs (green), non-targeting control sgRNAs (orange), and sgRNAs targeting LH00133 (red). Data shown correspond to the GFP-low fraction but are

representative of both GFP-low and GFP-high analyses. **a.** Scatter plot of $\log_{10}$-normalized sgRNA read counts in experimental samples versus control. **b**. Volcano plot showing sgRNA enrichment, plotted as $-\log_{10}$(p-value) versus $\log_2$ fold change. **c**.z-score plot ranking sgRNAs based on fold-change z-scores. **d.** Bar plot illustrating sgRNA targeting efficiency, with genes categorized by the number of significantly enriched sgRNAs. **e.**Volcano plot of sgRNA enrichment in the High population relative to the Mid population following CRISPR screening. Each point represents an individual sgRNA, plotted according to its log2 fold-change (x-axis) and −log10 *P* value (y-axis). Significant sgRNAs are shown in green, non-targeting sgRNAs in orange, and non-significant sgRNAs in gray. PinAPL-Py analysis identified 13 significantly enriched genes in the High versus Mid comparison.

Supplementary Fig 4. Validation of top CRISPRi screening hits in SNU449 cells. SNU449 cells stably expressing ACE2 were transduced with sgRNAs targeting top candidate genes identified from the CRISPRi screen and subsequently infected with VSV-ΔG pseudotyped with the Δ18-D614G spike variant. Cells transduced with the empty PSB700 vector served as controls. GFP intensity was measured by flow cytometry, with unstained cells used for gating. Data represent three independent experimental batches.

Supplementary Fig 5. Specificity of lncRNA KD effects viral entry. SNU449-ACE2-KRAB cells expressing sgRNAs targeting CNPY2-AS1 or RP11-314A20.5 were infected with VSV-ΔG-G pseudovirus. GFP fluorescence intensity was quantified by flow cytometry, with unstained cells used for gating. Cells transduced with the empty PSB700 vector served as controls.

Supplementary Fig 6. Tissue-wide expression of CNPY2-AS1 across human organs. Violin plot showing expression levels of CNPY2-AS1 across multiple human tissues. Data were obtained from the Genotype-Tissue Expression (GTEx) project. The y-axis represents transcript abundance measured as transcripts per million (TPM), and the x-axis indicates tissue types. The width of each violin reflects the distribution of expression values within each tissue. Black box plots denote the median and interquartile range.

Supplementary Fig 7. Orthogonal KO of CNPY2-AS1 confirms its role in SARS-CoV-2 spike–mediated entry. a. Schematic illustration of the CRISPR Cas9 KO strategy targeting the lncRNA CNPY2-AS1. An ~150 bp genomic region encompassing the 3′ splice acceptor site of the final exon was deleted to disrupt proper splicing and induce transcript destabilization. **b.** VSVΔG-G–mediated infection following CNPY2-AS1 KO in SNU449-ACE2 cells. Infection was quantified by flow cytometric measurement of GFP-positive cells following VSVΔG-G pseudovirus infection. Data represent mean ± SD from independent experiments.

Supplementary Fig 8. CNPY2-AS1 KO does not alter ACE2 expression or SARS-CoV-2 spike protein levels in A549 cells. Top. ACE2 receptor expression in control and CNPY2-AS1 KO A549 cells, assessed by flow cytometry. No significant change was observed. Bottom. SARS-CoV-2 spike mediated entry following VSVΔG-S pseudovirus infection of control and

CNPY2-AS1 KO A549 cells. Infection levels were quantified. Data represent mean ± SD from n = 3 independent experiments.

Supplementary Fig 9. Global transcriptomic changes in control and CNPY2-AS1KO cells. **a.** PCA of RNA-sequencing data from control and CNPY2-AS1 KO samples. The scatter plot shows the distribution of samples along the first two principal components (PC1 and PC2), with the percentage of variance explained by each component indicated on the axes. Each point represents an individual sample; control samples are shown in blue and KO samples in red. **b**. Heatmap of DEGs between control and CNPY2-AS1 KO cells. Hierarchical clustering was performed on both genes and samples based on expression similarity. The color scale represents relative gene expression levels, with blue indicating low expression and red indicating high expression. **c.** Volcano plot showing differential gene expression between control and CNPY2-AS1 KO cells. Significantly upregulated genes are shown in red, significantly downregulated genes in blue, and non-significant genes in gray.

Supplementary Fig 10. PLSCR1 is upregulated upon CNPY2-AS1 loss and efficiently depleted by CRISPR KO. **a**. Volcano plot showing differential gene expression in CNPY2-AS1 KO cells. PLSCR1 is highlighted as a significantly upregulated gene in CNPY2-AS1 KO cells. **b**. Quantitative PCR analysis validating PLSCR1 depletion in PLSCR1 KO cells. Relative mRNA expression of PLSCR1 and CNPY2-AS1 is shown for control and PLSCR1 KO cells. PLSCR1 expression is efficiently reduced, while CNPY2-AS1 shows a modest increase. Expression levels were normalized to housekeeping genes and are presented relative to control cells. Data represent mean ± SD from n = 3 independent experiments. Statistical significance was determined using a two-tailed Student's t-test ($P < 0.05$, $*P < 0.01$, $**P < 0.001$; ns, not significant).

Supplementary Fig11. Transcription factor enrichment analysis of upregulated genes identifies IRF7 centered regulatory programs. ChEA3 transcription factor enrichment analysis identifies IRF7-centered regulatory programs. ChEA3 analysis was performed on genes upregulated in the RNA-seq dataset. The bubble plot shows the top 10 enriched transcription factors ranked by mean rank (lower is better). Bubble size represents the number of upregulated target genes associated with each transcription factor. IRF7 is among the highest-ranked regulators, consistent with its central role in type I interferon signaling.

Supplementary Fig. 12. Increased IRF7 abundance and activation in CNPY2-AS1 knockout cells. a. Representative immunoblot of IRF7 protein levels in control and CNPY2-AS1 KO cells following rapid cell fractionation (REAP). **b.** Representative immunoblot (left) and densitometric quantification (right) of phosphorylated IRF7 (p-IRF7) in control and CNPY2-AS1

KO cells following REAP fractionation. Data are presented as mean ± SD from independent biological replicates.

Supplementary Fig. 13 Validation of the CNPY2-AS1–TXNRD1 interaction and their subcellular localization. Western blot validation of TXNRD1 enrichment in the CNPY2-AS1 RNA pull-down. a. Biotinylated CNPY2-AS1 RNA or scrambled control RNA was immobilized on streptavidin magnetic beads and incubated with SNU449-ACE protein lysates. Following washing, bead-bound proteins were eluted and analyzed by western blotting using an anti-TXNRD1 antibody. (Left) Representative western blot showing TXNRD1 enrichment in three independent CNPY2-AS1 pull-downs compared with three scrambled RNA controls. The band corresponding to TXNRD1 was detected at approximately 70 kDa. (Right) Densitometric quantification of the TXNRD1 band from two independent pull-down experiments. The TXNRD1 signal was normalized to the mean signal of the scrambled RNA control, which was set to 1. CNPY2-AS1 pull-down showed a mean relative TXNRD1 enrichment of 5.5-fold compared with scrambled RNA. Data are presented as mean ± SD, *n* = 2 independent experiments.
b. Representative analysis of CNPY2-AS1 subcellular localization in SNU449, HL-60, and U2OS cells by cytoplasmic and nuclear fractionation followed by RT-qPCR. CNPY2-AS1 was detected in both nuclear and cytoplasmic fractions across the three cell lines. Fraction purity was assessed using appropriate nuclear and cytoplasmic compartment-specific markers. **c** Representative immunofluorescence images showing the subcellular localization of TXNRD1 in SNU449 cells. TXNRD1 was predominantly localized to the cytoplasm.

Supplementary Fig. 14. H2O2 treatment phenocopies CNPY2-AS1 KO effects on ROS levels and STAT1 localization. a. Induction of interferon signaling by oxidative stress. Wild-type cells were treated with hydrogen peroxide (H_2O_2; 150 μM), and expression of STAT1 and the interferon-stimulated gene PLSCR1 was assessed. Data represent mean ± SD from n = 3 independent experiments; statistical significance was determined using a two-tailed Student's t-test (P < 0.05, *P < 0.01, **P < 0.001; ns, not significant). **b.** Quantification of intracellular ROS levels following H2O2 treatment, measured by DCFDA fluorescence. Data are shown from n ≥ 3 biological replicates and presented as mean ± SD. *P < 0.05, **P < 0.01, ***P < 0.001, ****P < 0.0001.**c.** Representative immunofluorescence images and quantification of the STAT1 nuclear/cytoplasmic ratio following H2O2 treatment. Nuclei were stained with DAPI, and STAT1 is shown in magenta. Quantification was performed from n ≥ 3 biological replicates, and data are presented as mean ± SD. *P < 0.05, **P < 0.01, ***P < 0.001, ****P < 0.0001.

Supplementary Fig.15. Validation of TXNRD1 knockdown by siRNA.
a. Representative western blot showing TXNRD1 protein levels in siControl and siTXNRD1-transfected cells. GAPDH was used as a loading control.
b. RT-qPCR analysis of TXNRD1 relative expression in siControl and siTXNRD1-transfected cells. Data are presented as mean ± SD from n = [insert number] replicates. *P < 0.05, **P < 0.01, ***P < 0.001, ****P < 0.0001**. c.** Representative immunofluorescence images showing

STAT1 localization in control, CNPY2-AS1 KO, CNPY2-AS1 KO + siRNA TXNRD1, and control. Nuclei were stained with DAPI, and STAT1 is shown in pink

Supplementary Fig.16. Effects of TRi-1 treatment on TXNRD1 expression, viral infection, ROS levels, and STAT1 localization. a. Left: Representative western blot and quantification of TXNRD1 protein levels in ACE, ACE treated with TRi-1, and CNPY2-AS1 KO cells. GAPDH was used as a loading control. Right: Western blot quantification was performed from three biological replicates, and data are presented as mean ± SD. *P < 0.05, **P < 0.01, ***P < 0.001, ****P < 0.0001. **b.** Quantification of GFP-positive infected cells following TRi-1 treatment, measured by flow cytometry. Data are shown from n ≥ 3 independent experiments and presented as mean ± SD. *P < 0.05, **P < 0.01, ***P < 0.001, ****P < 0.0001. **c.** Quantification of intracellular ROS levels following TRi-1 treatment, measured by DCFDA fluorescence. Data are shown from n ≥ 3 independent experiments and presented as mean ± SD. *P < 0.05, **P < 0.01, ***P < 0.001, ****P < 0.0001. **d.** Left: Representative immunofluorescence images and quantification of the STAT1 nuclear/cytoplasmic ratio in control, TRi-1-treated, and CNPY2-AS1 KO cells. Nuclei were stained with DAPI, and STAT1 is shown in magenta. Right: Quantification was performed from n ≥ 3 independent experiments, and data are presented as mean ± SD. *P < 0.05, **P < 0.01, ***P < 0.001, ****P < 0.0001.

Supplementary Fig.17. Representative STAT1 immunofluorescence images following treatments. Representative immunofluorescence images showing STAT1 localization in control, CNPY2-AS1 KO, CNPY2-AS1 KO + DMF, and CNPY2-AS1 KO + sodium selenite cells. Nuclei were stained with DAPI, and STAT1 is shown in pink

Supplementary Fig.18. Reduced CNPY2-AS1 expression is associated with severe COVID-19 and poor clinical outcomes. Violin plots showing expression levels of CNPY2-AS1 in COVID-19 patient cohorts. Expression values are shown as voom-transformed counts. (Top) Comparison of CNPY2-AS1 expression between surviving (n = 423) and non-surviving (n = 59) COVID-19 patients. (Bottom) Comparison of CNPY2-AS1 expression between non-critical (n = 301) and critical (n = 181) COVID-19 cases. Individual data points represent patient samples. Box plots indicate the median and interquartile range. FDR adjusted p-values and $\log_2$ fold changes are shown for each comparison

Supplementary Fig. 1

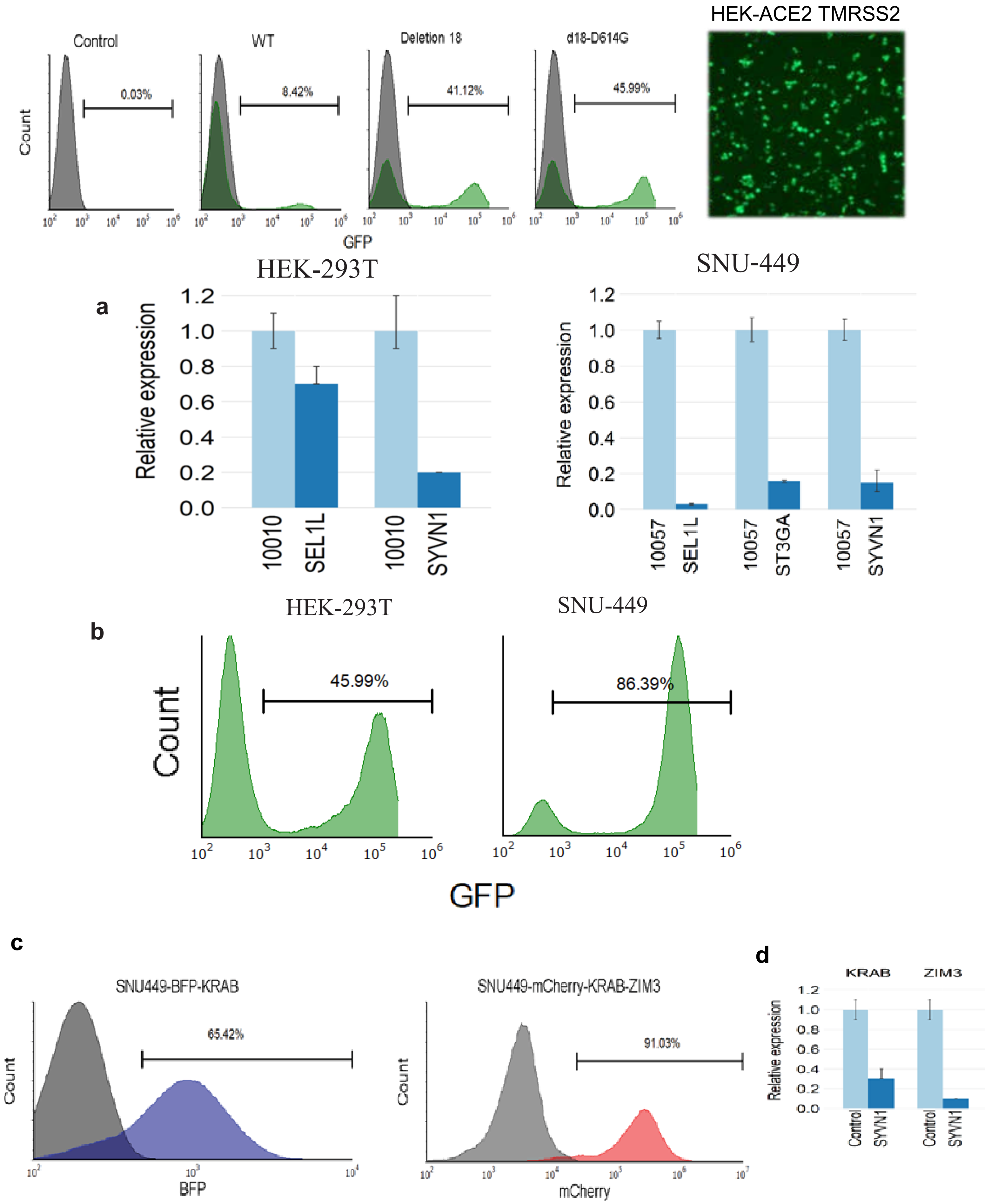

Supplementary Fig. 2

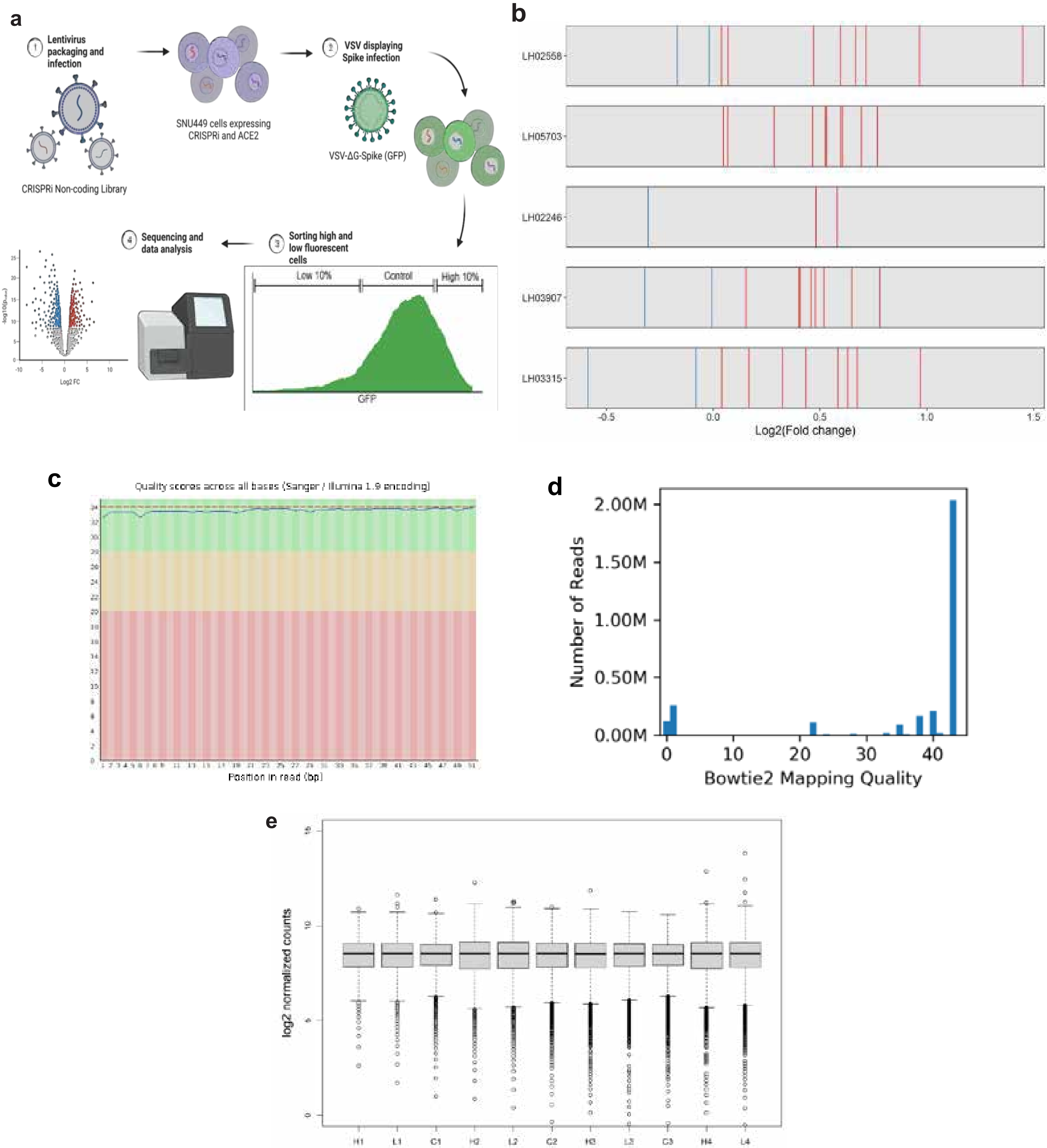

Supplementary Fig.3

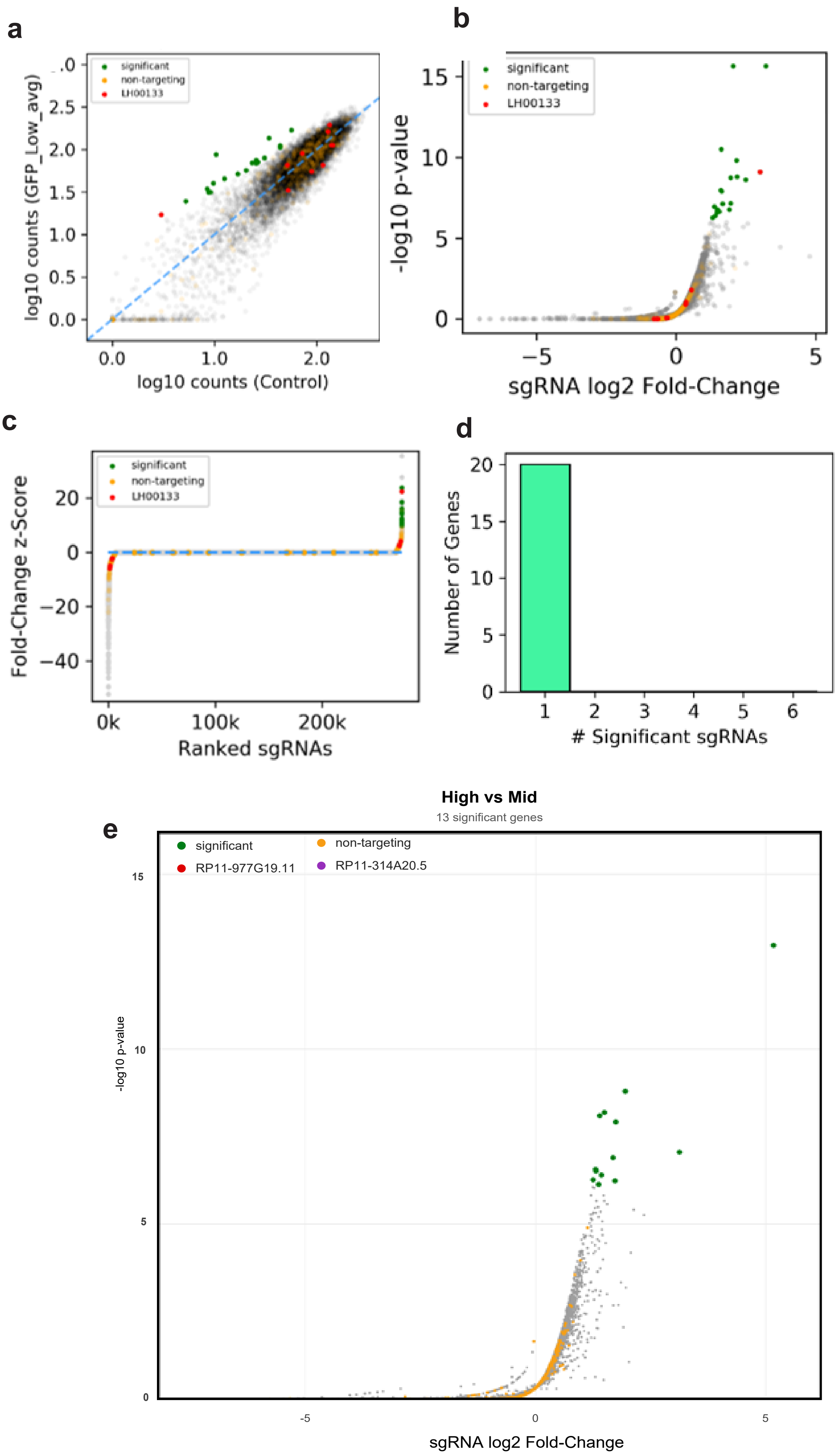

Supplementary Fig. 4

a

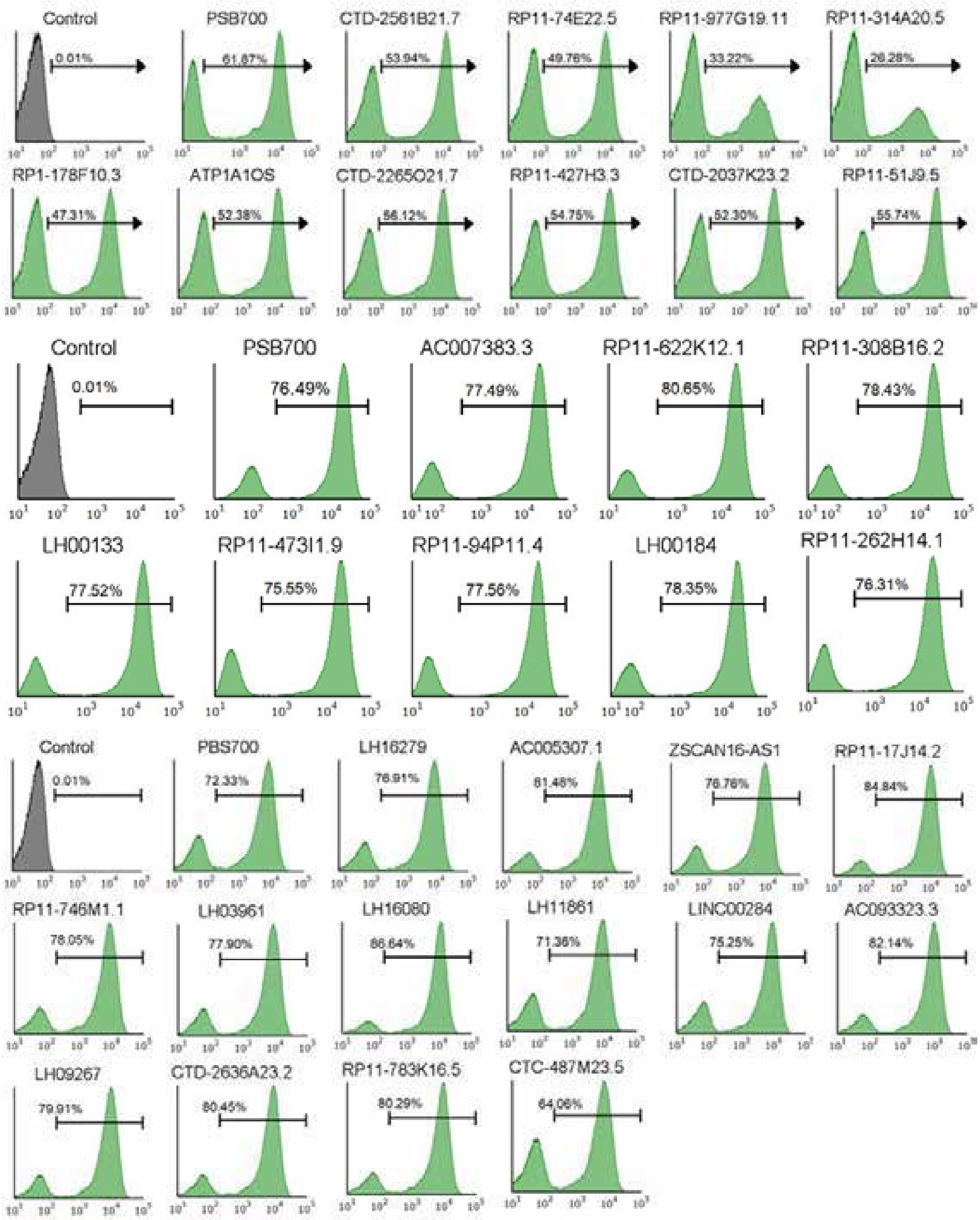

Supplementary Fig. 5

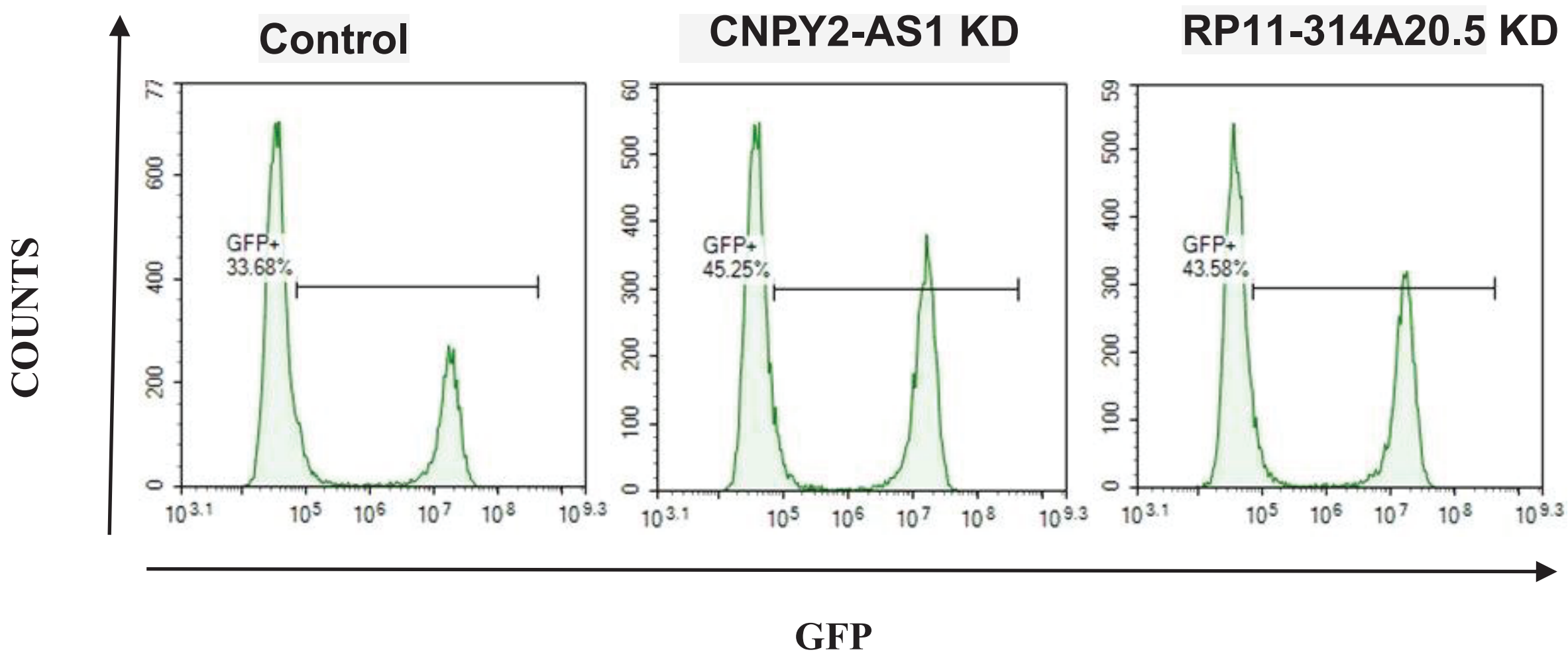

Supplementary Fig. 6

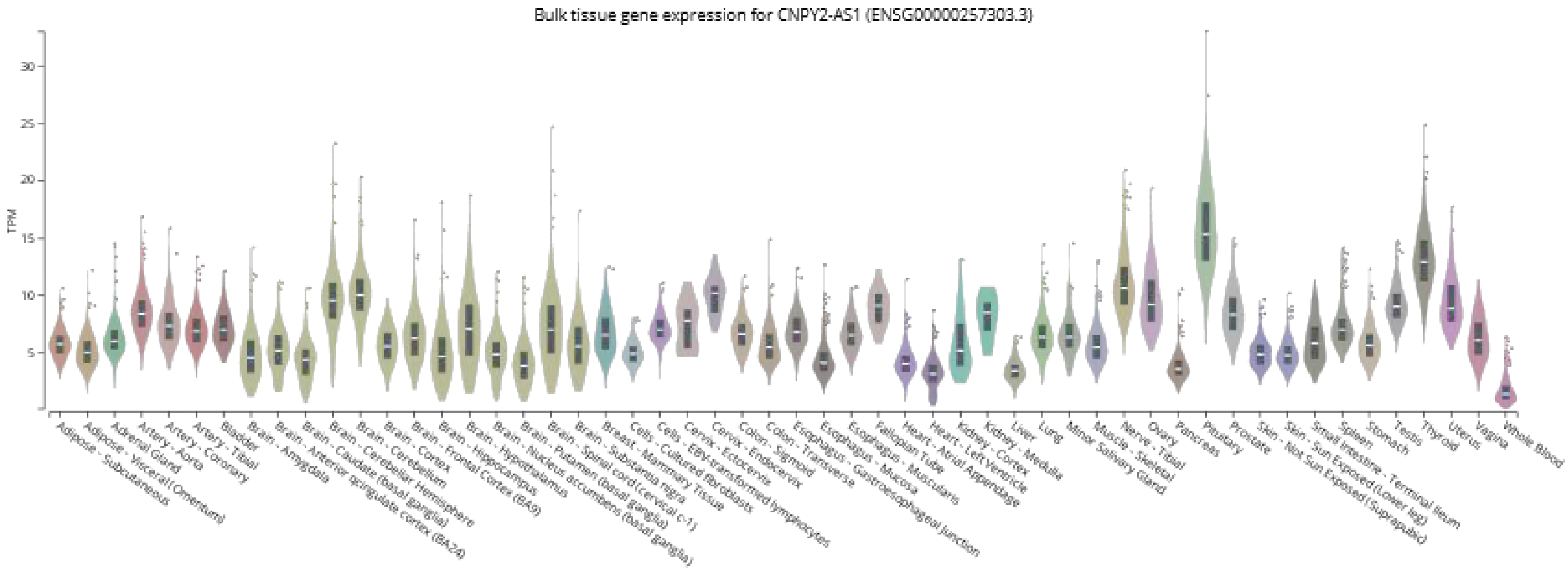

Supplementary Fig. 7

a

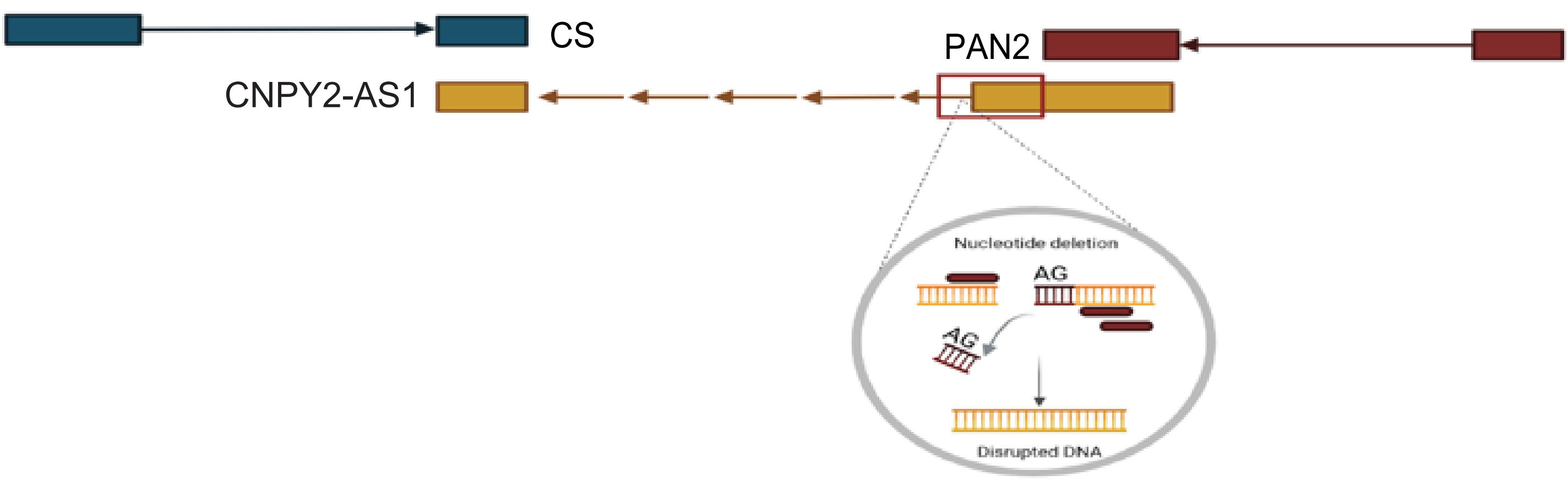

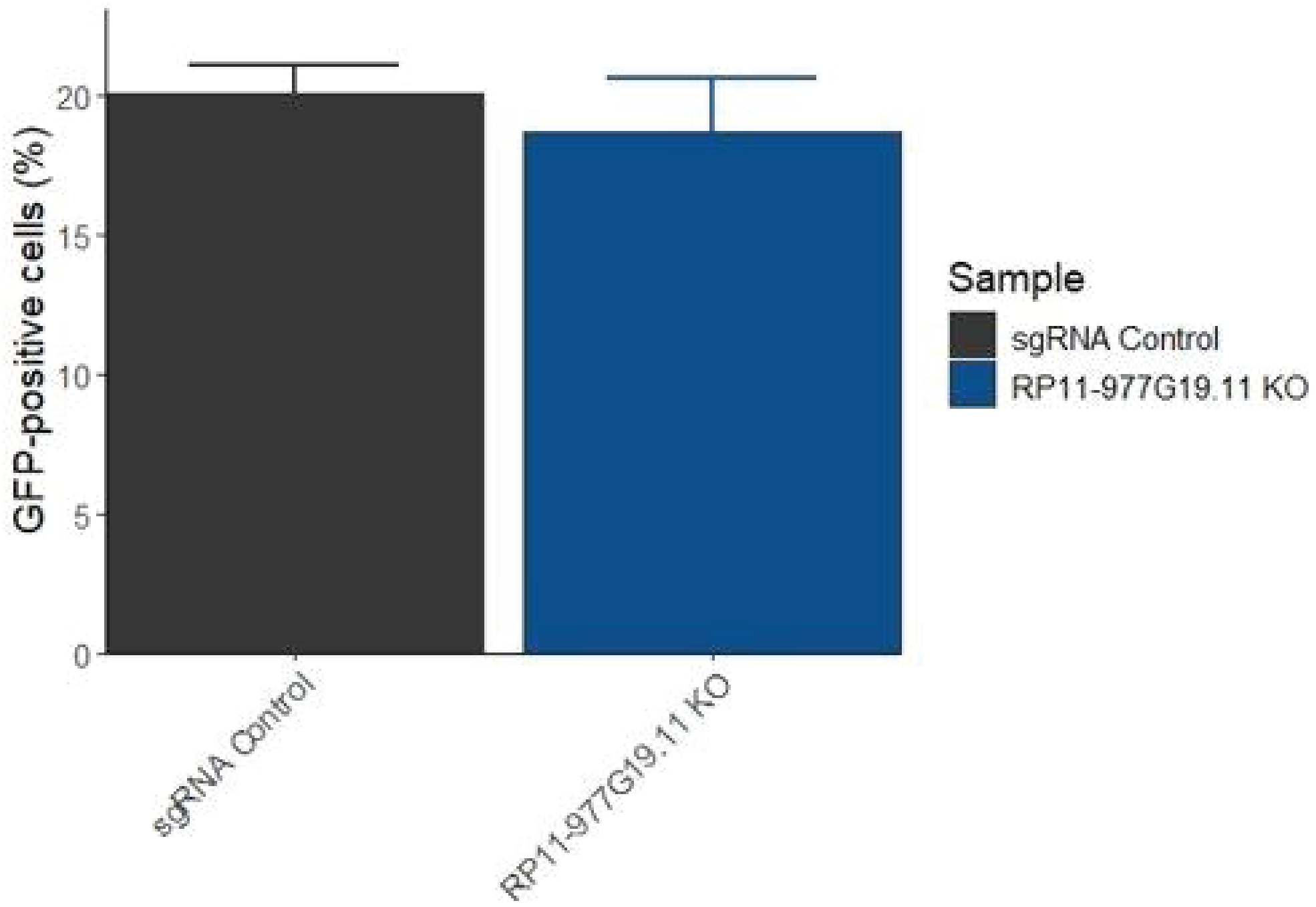

Supplementary Fig. 8

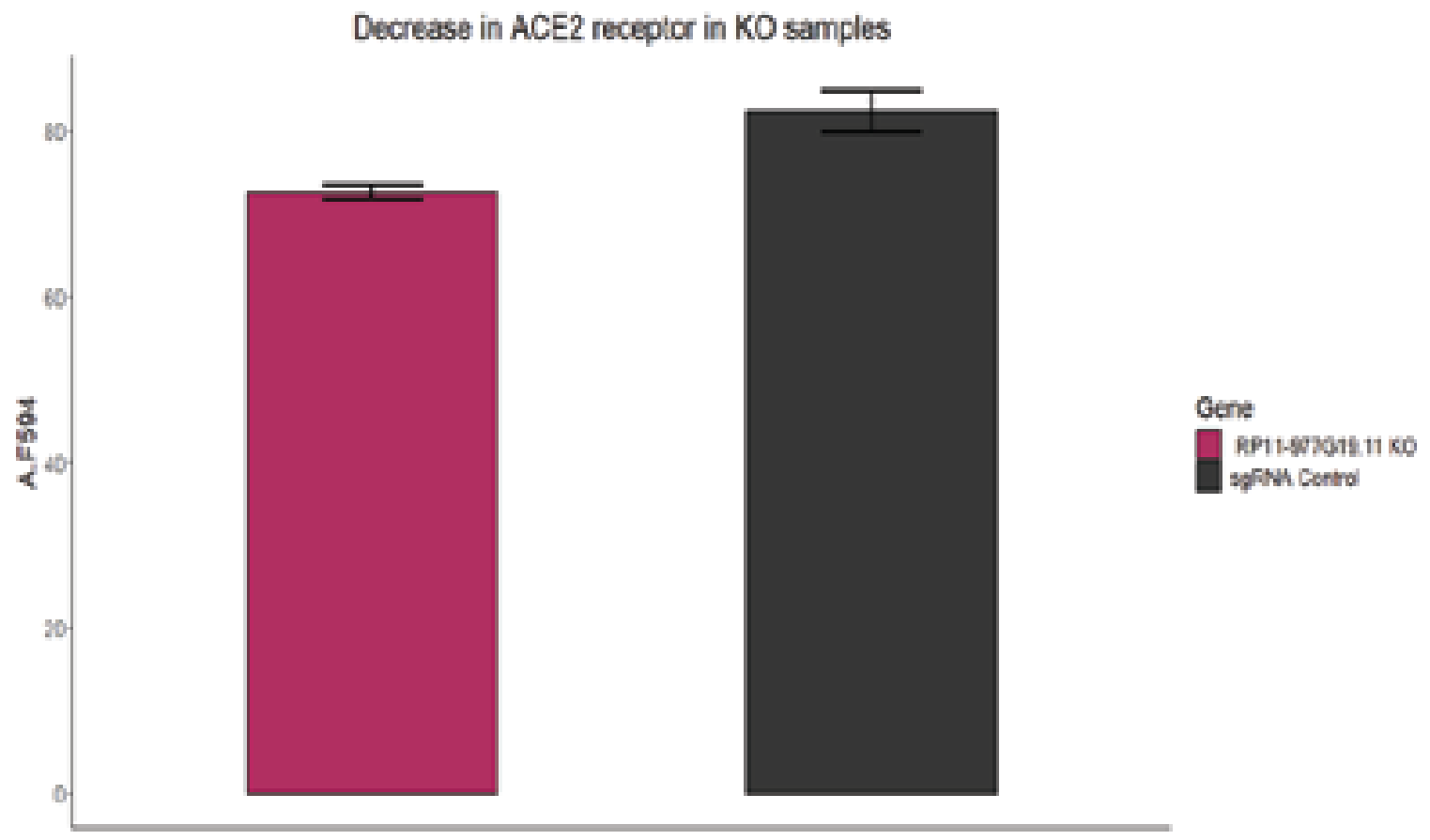

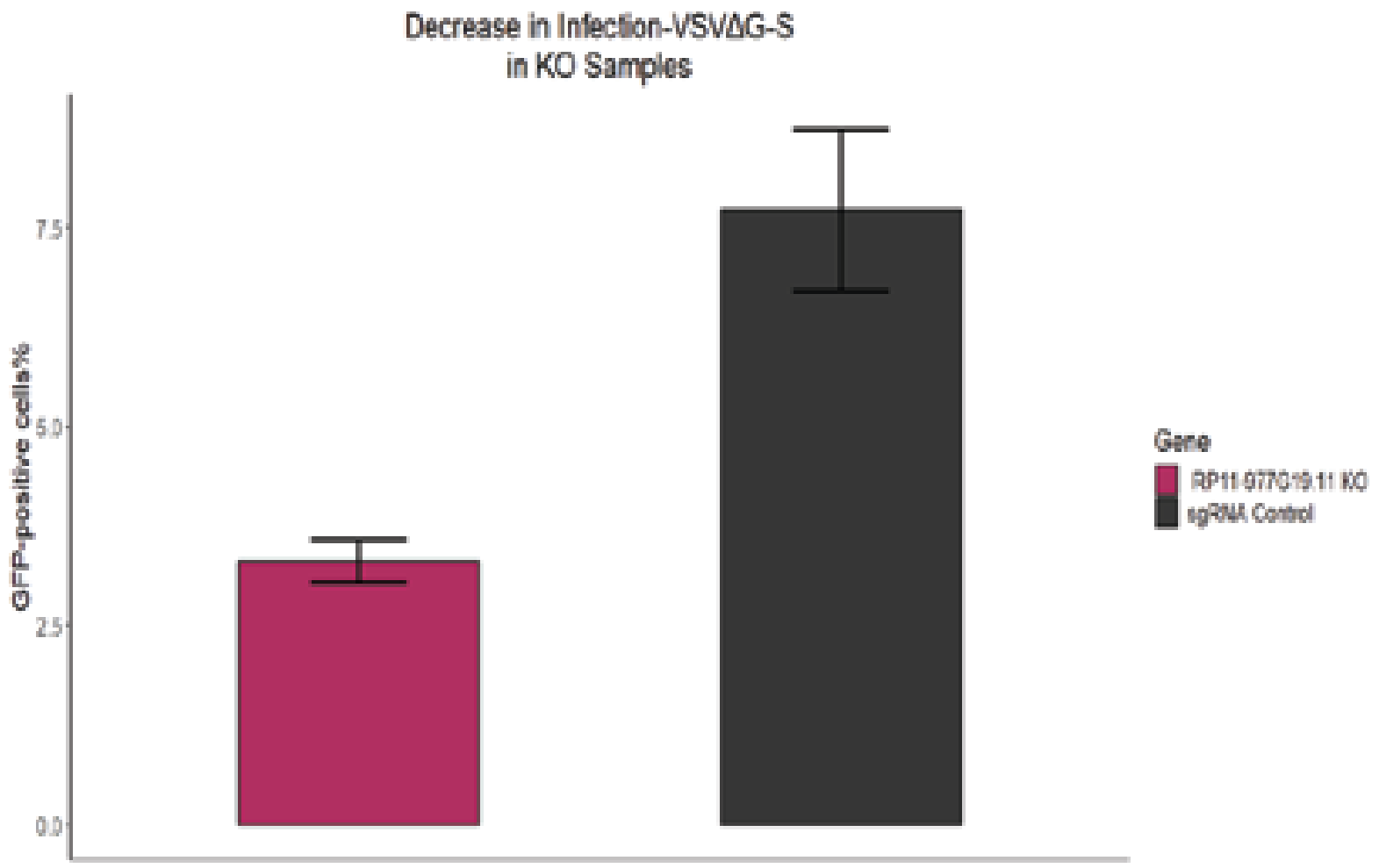

Supplementary Fig. 9

a

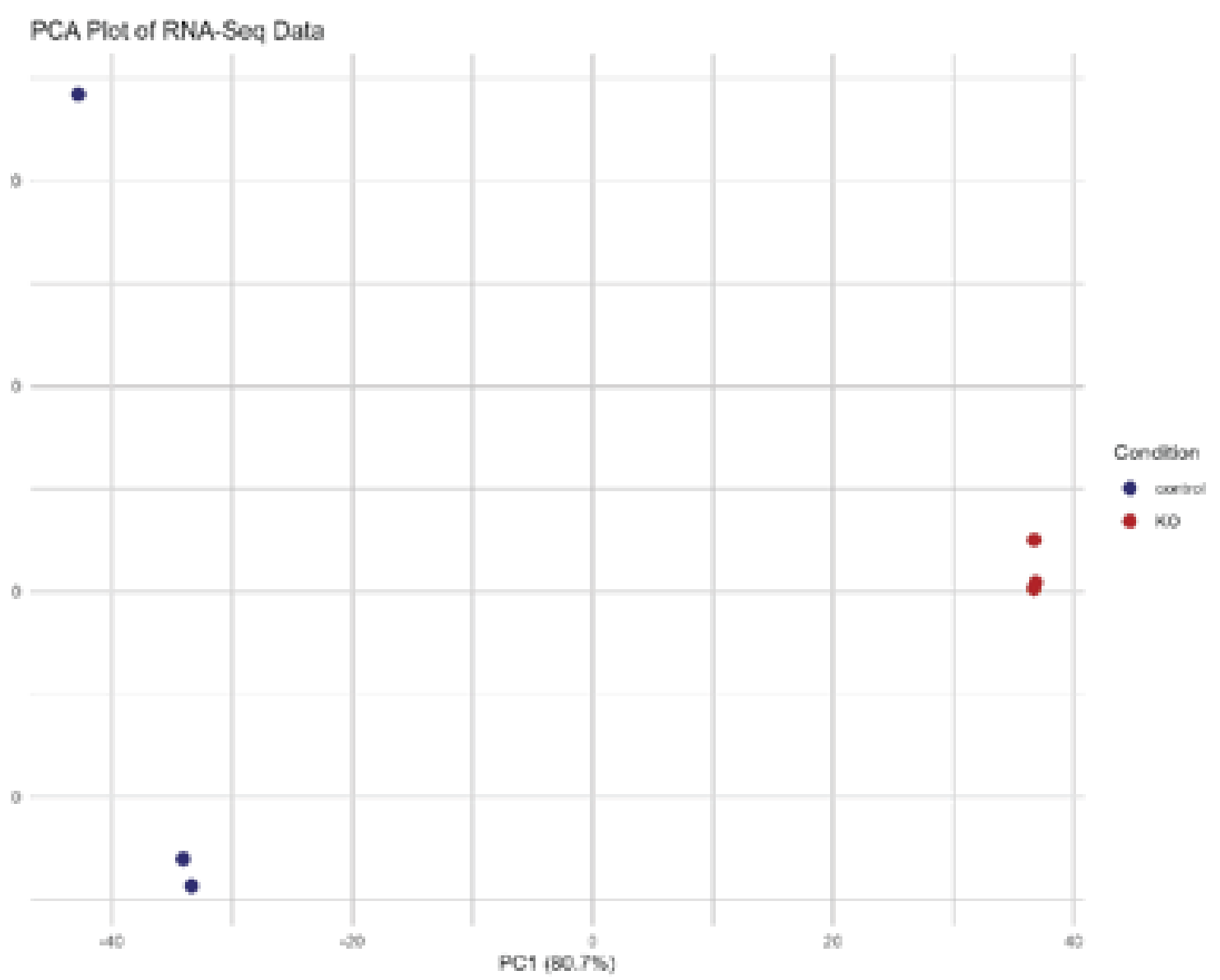

b

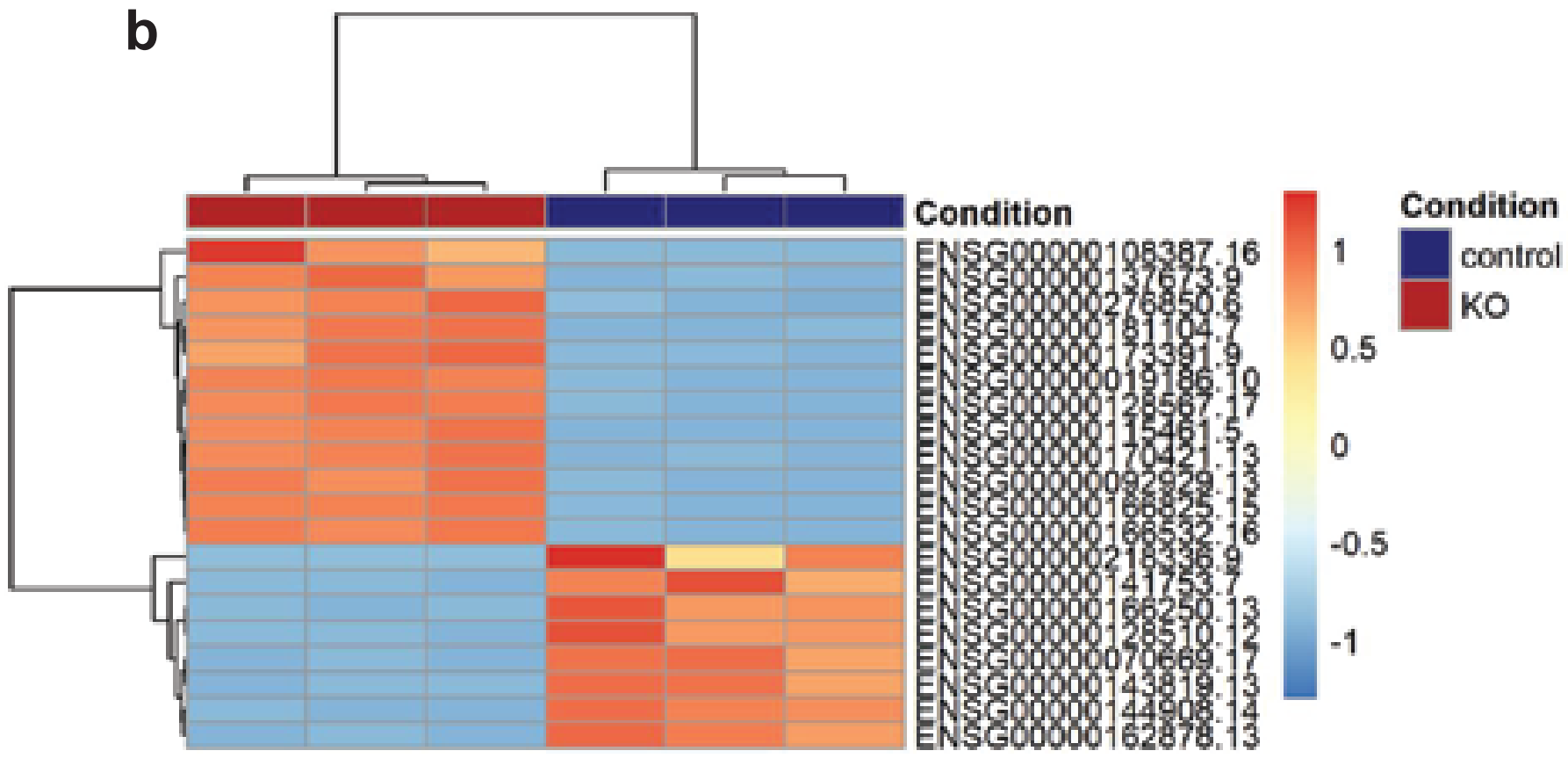

c

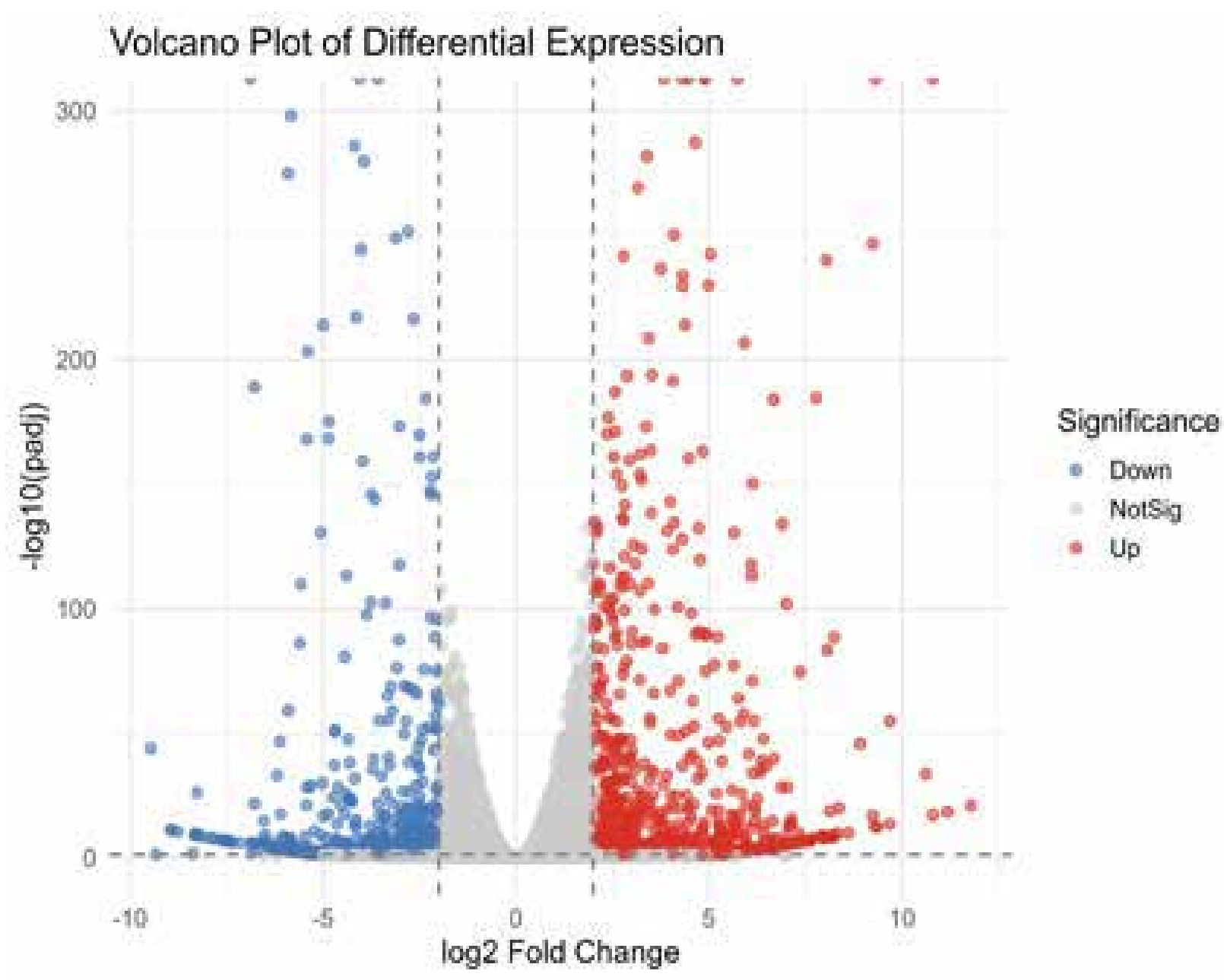

Supplementary Fig. 10

a

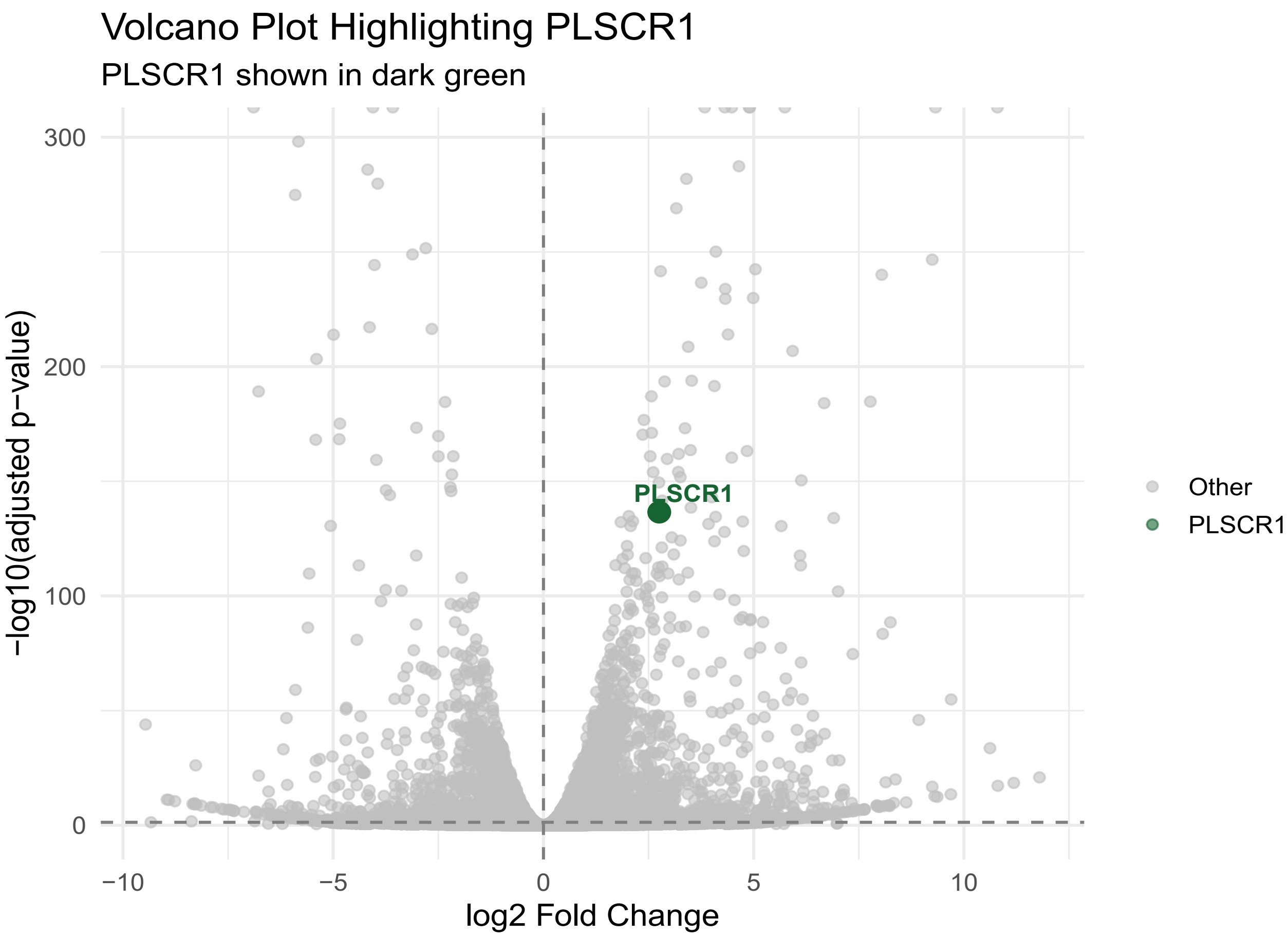

b

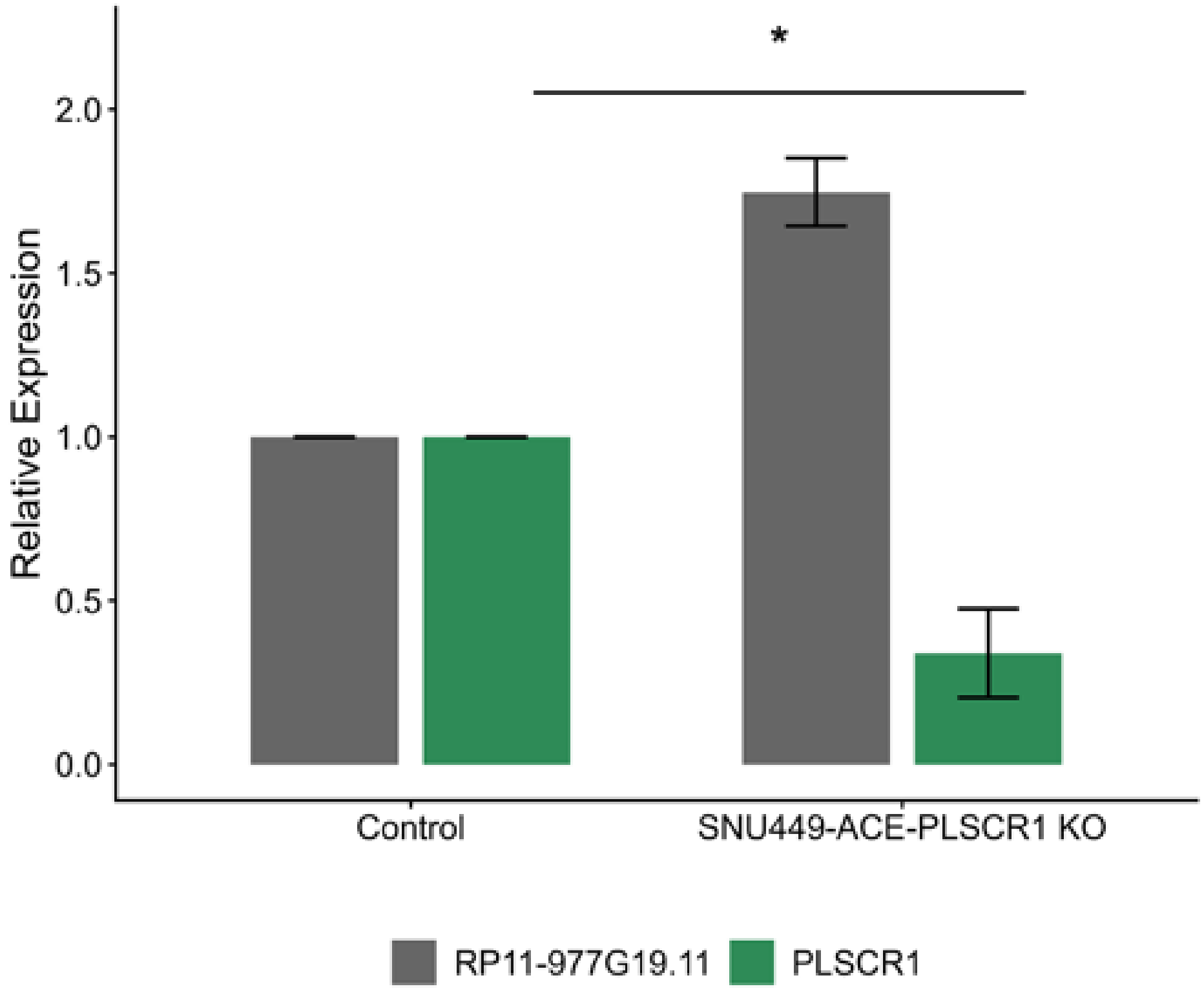

Supplementary Fig.11

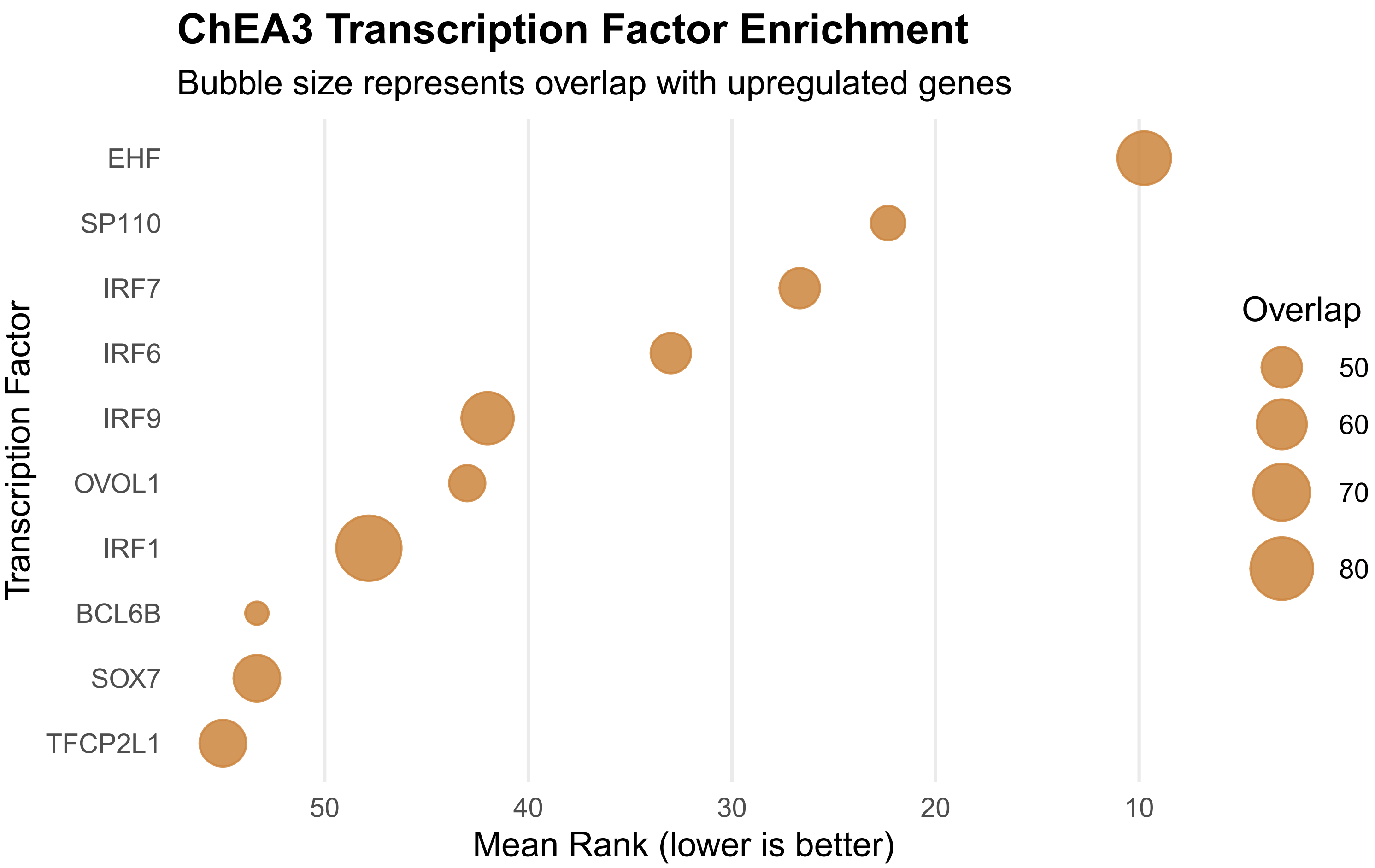

Supplementary Fig.12

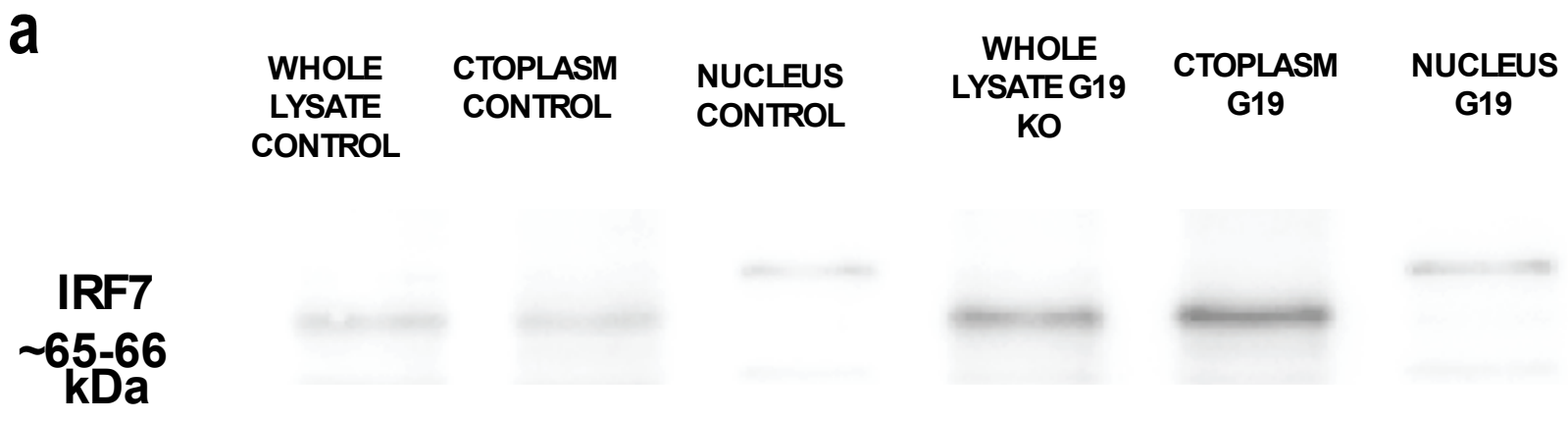

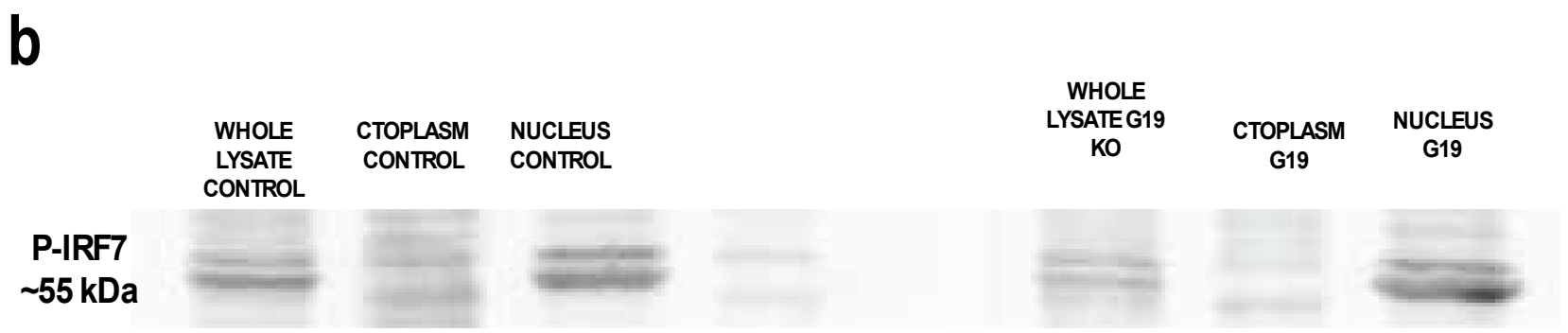

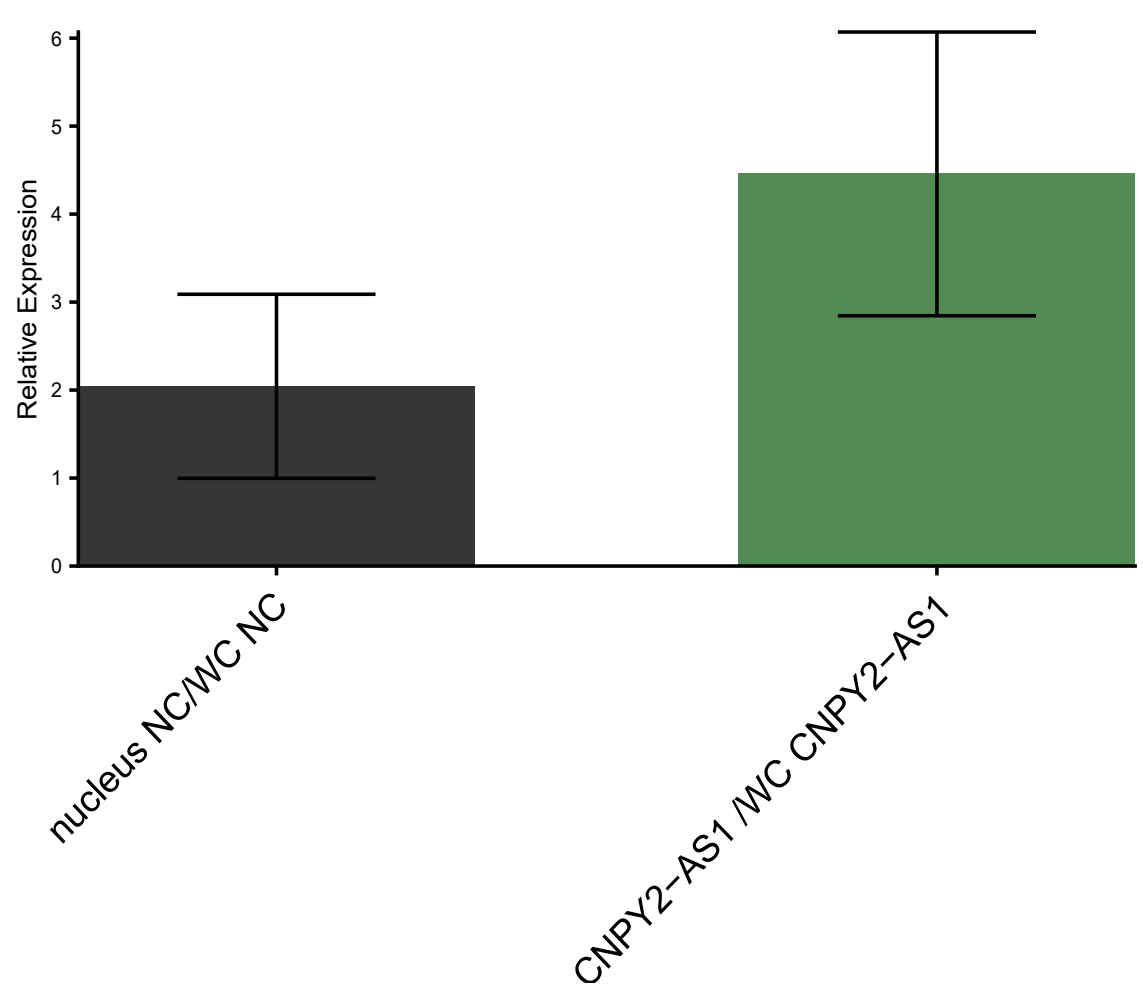

Supplementary Fig.13

a

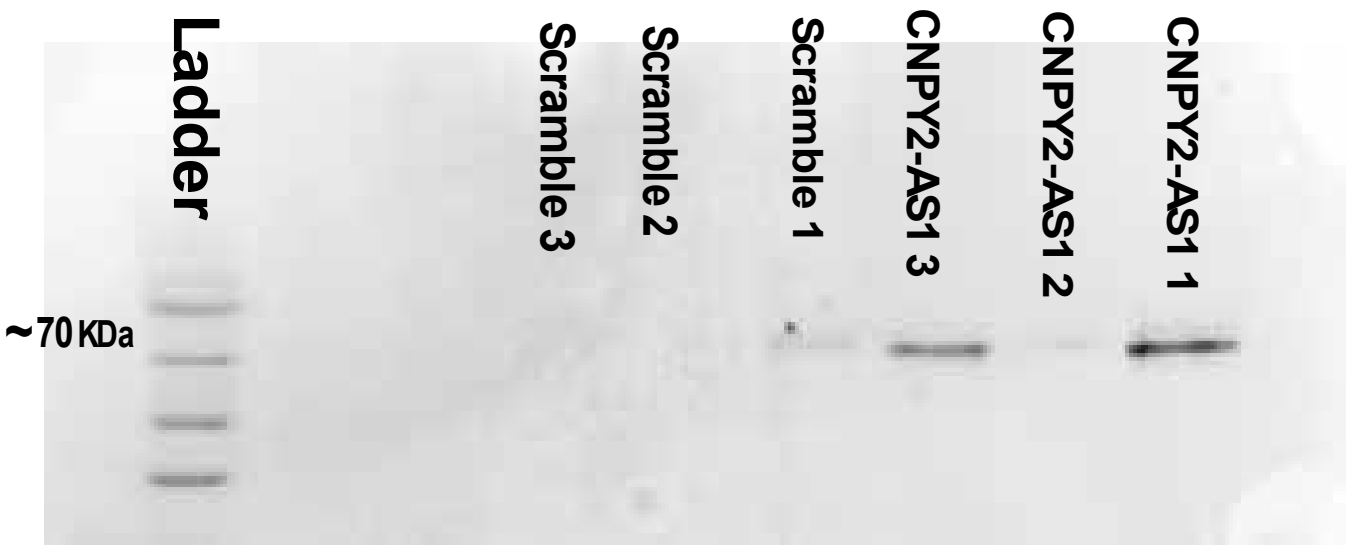

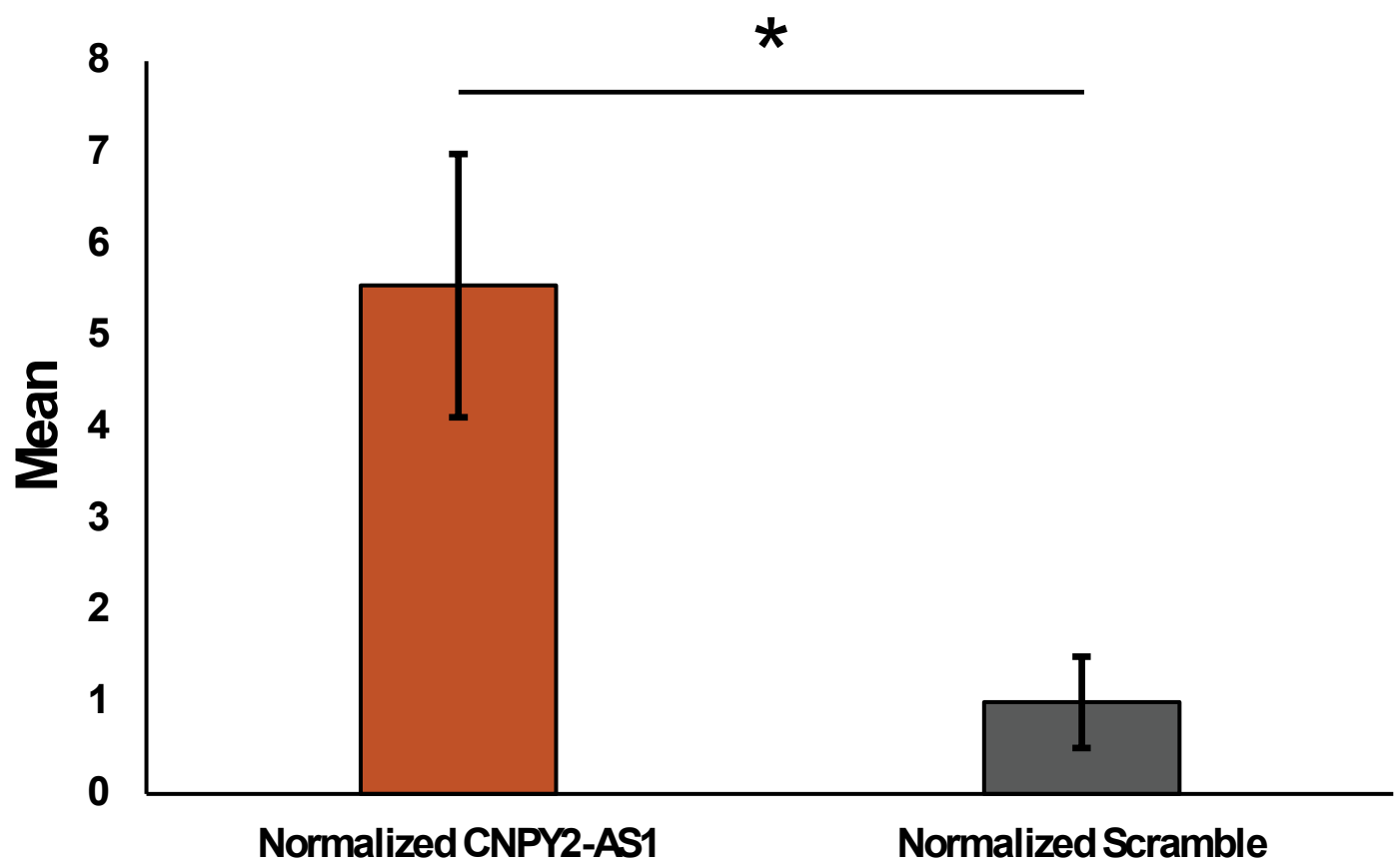

Cell Fractionation: Determining the Subcellular Localization of G19 in different cell lines

b

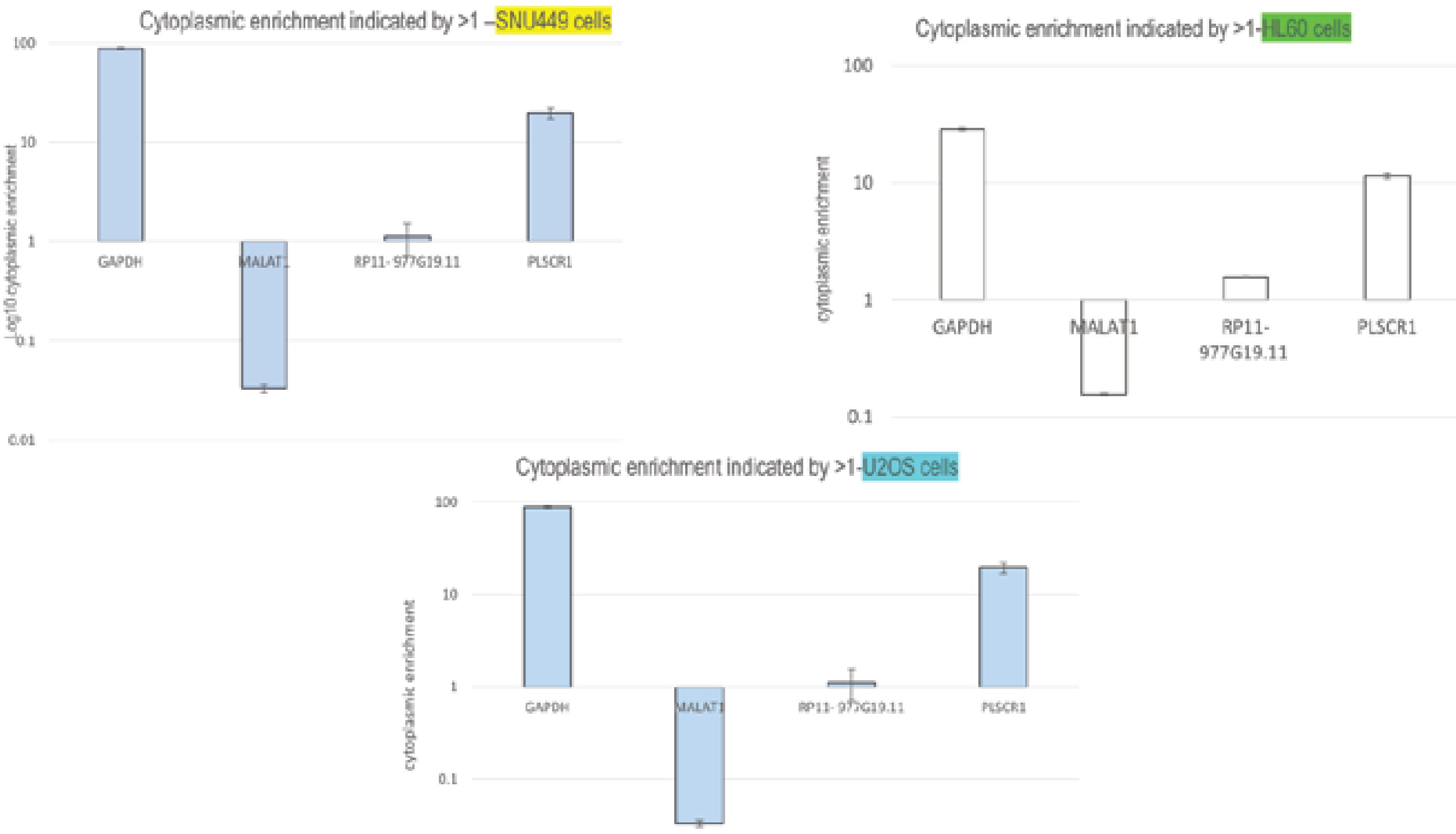

c

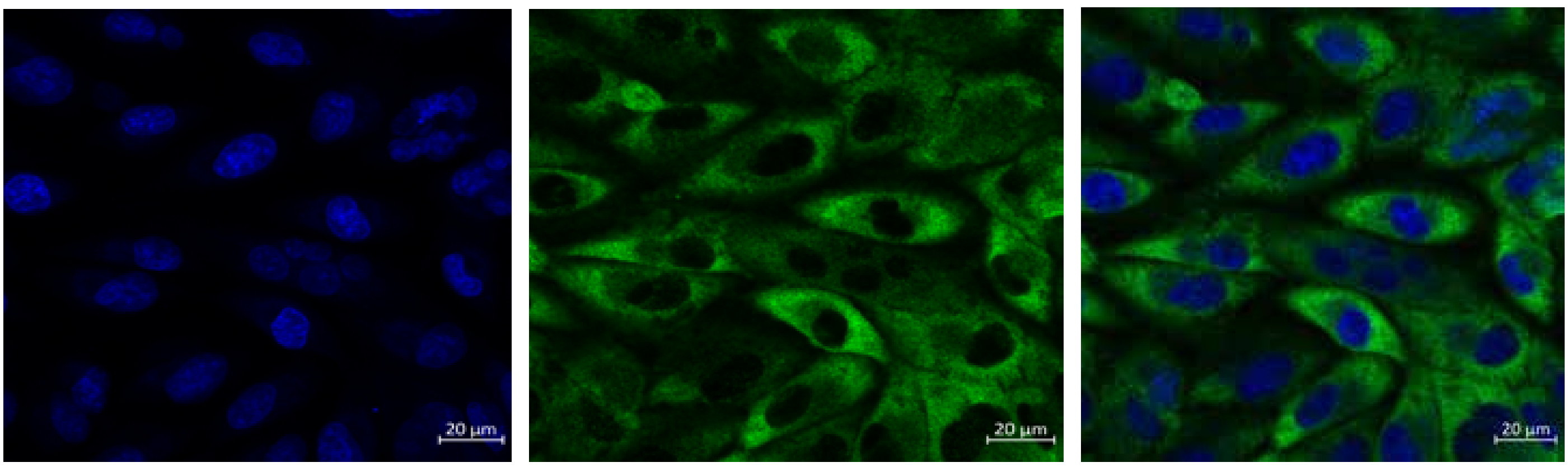

Supplementary Fig.14

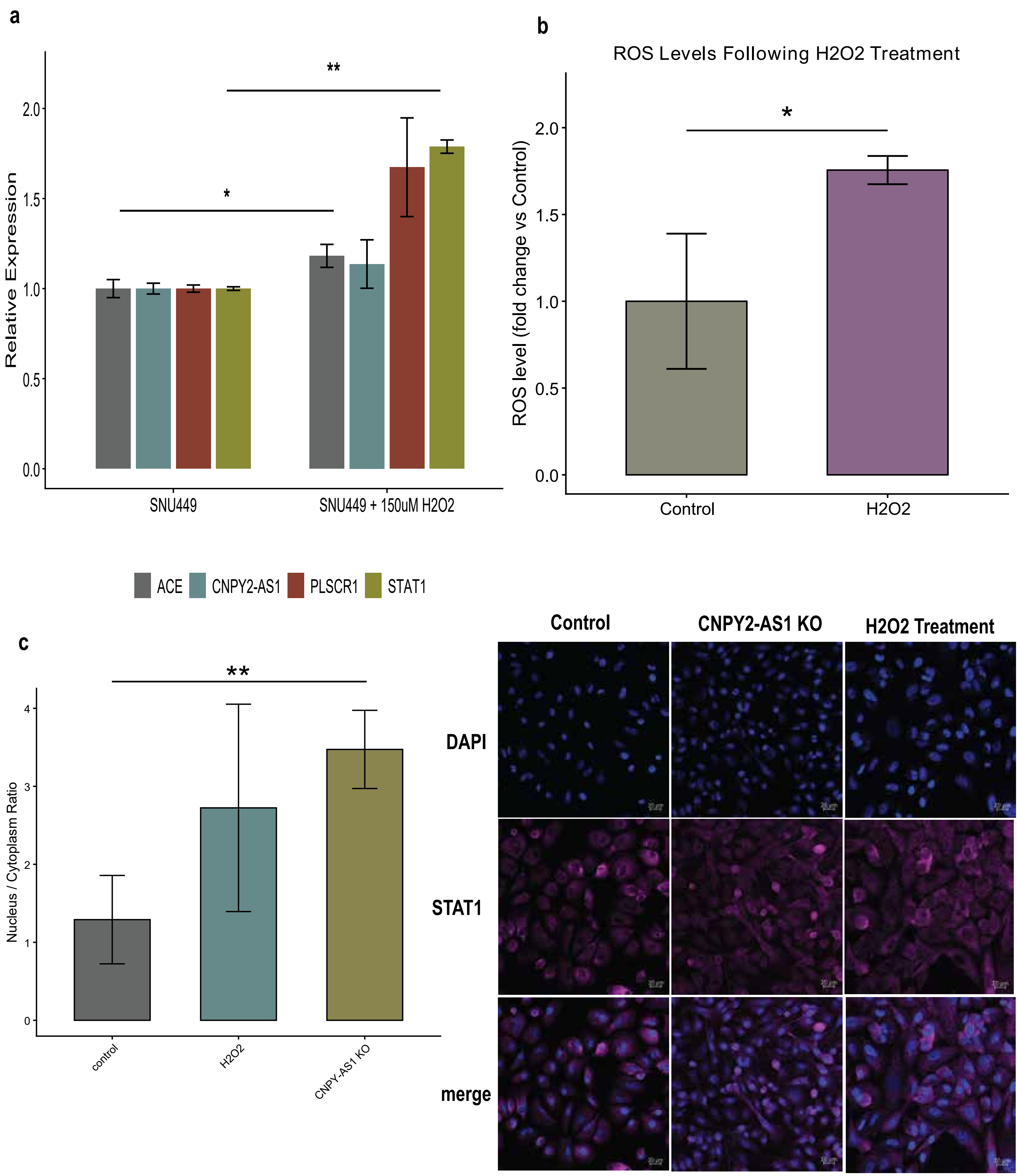

Supplementary Fig.15

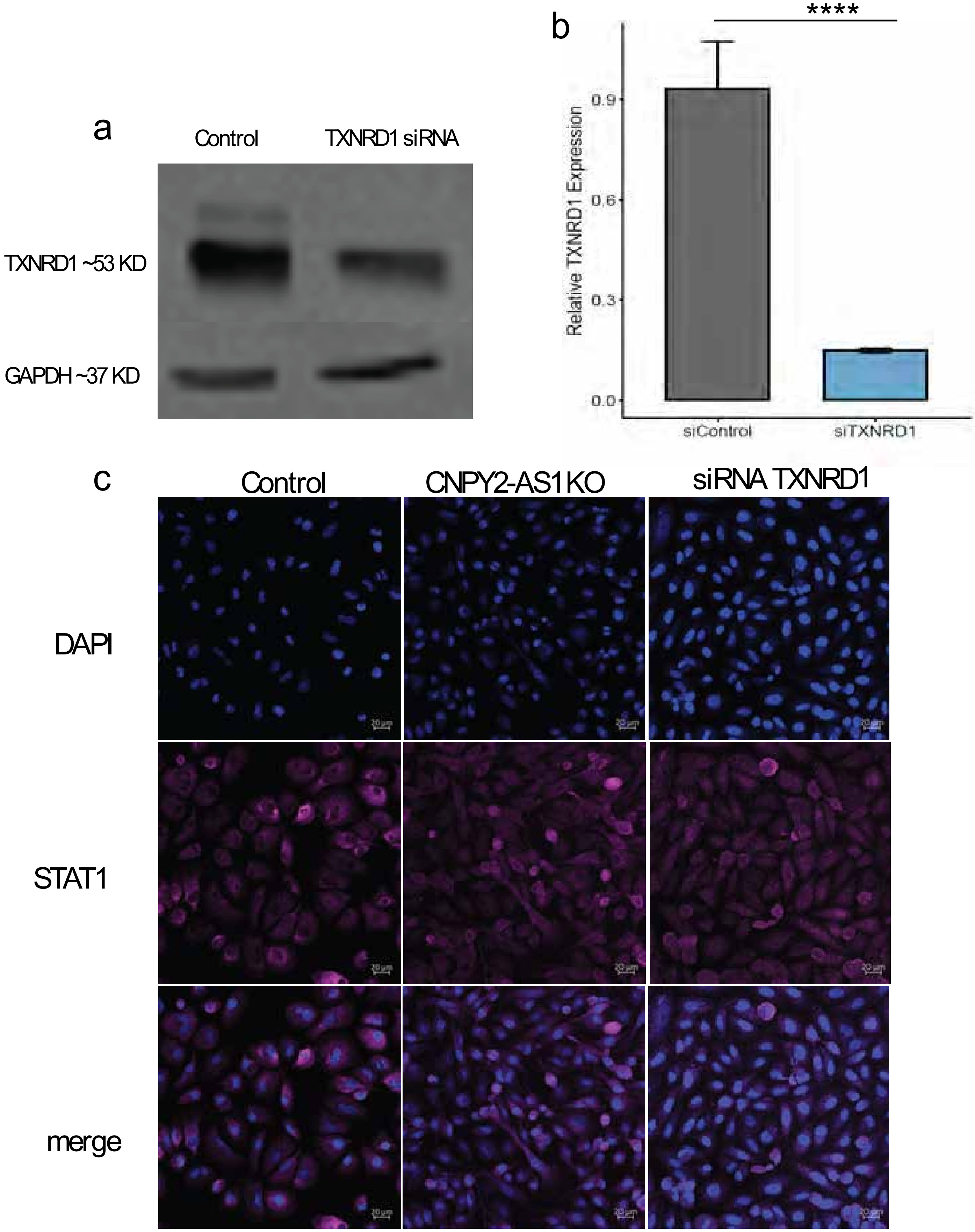

Supplementary Fig.16

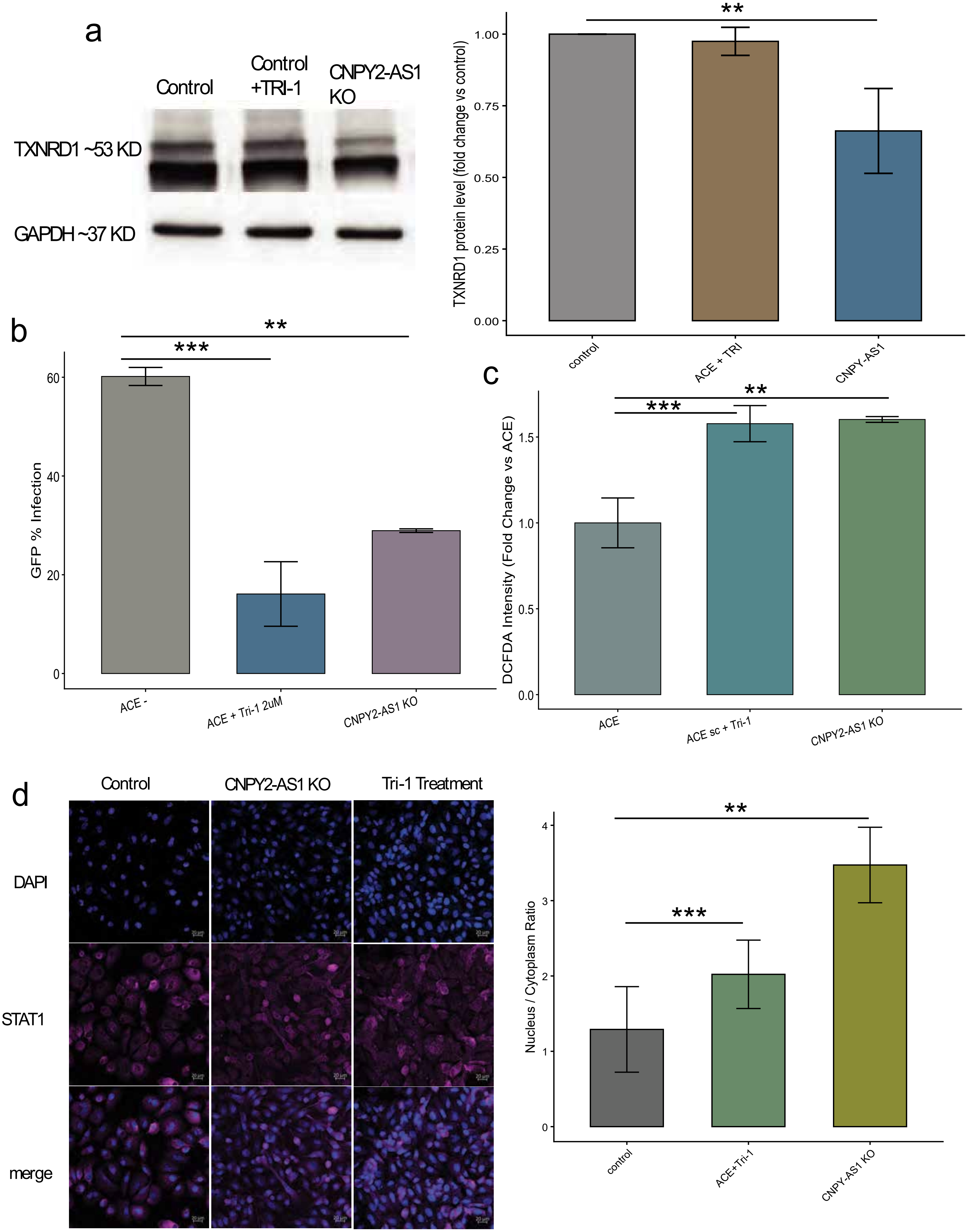

Supplementary Fig.17

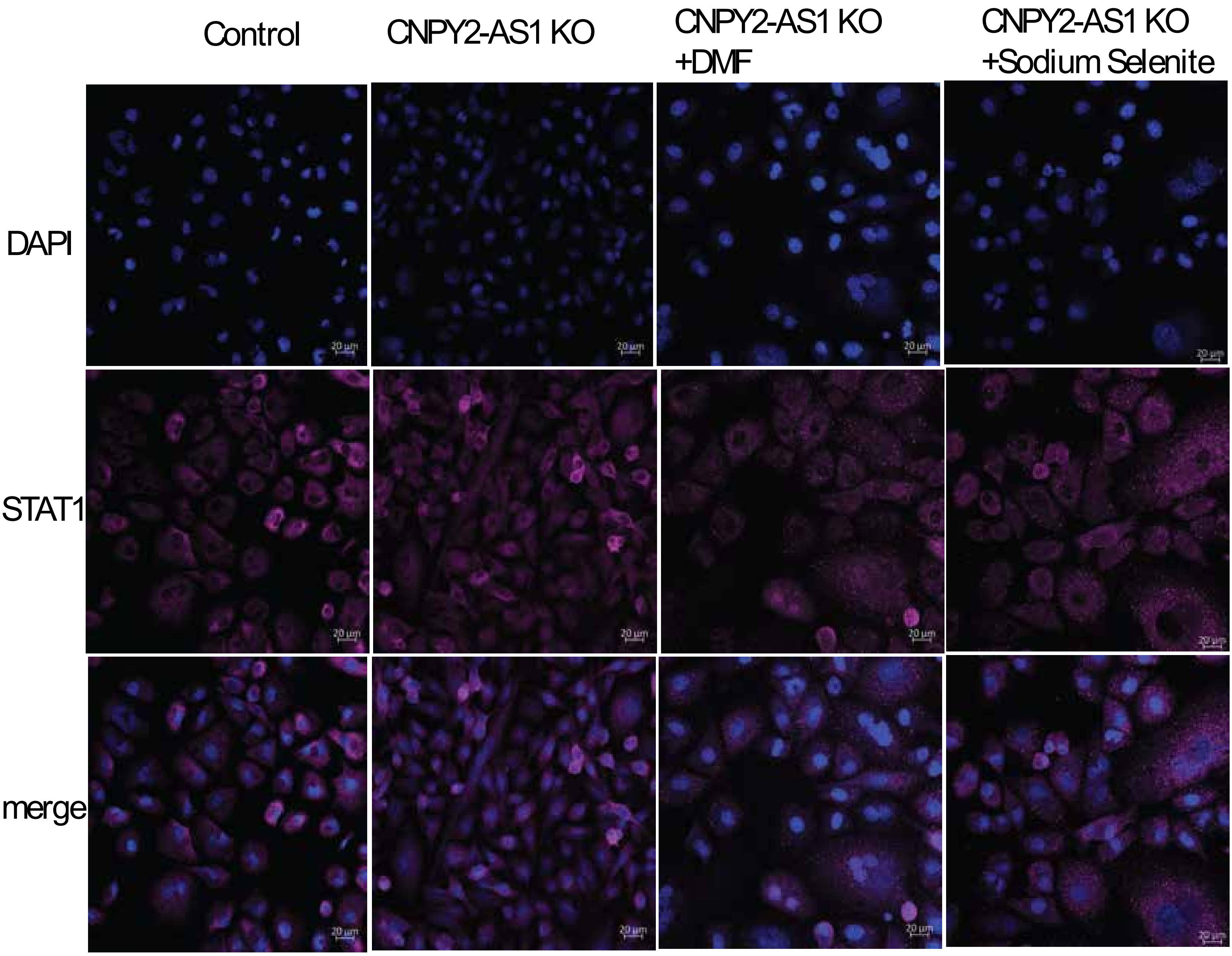

Supplementary Fig.18

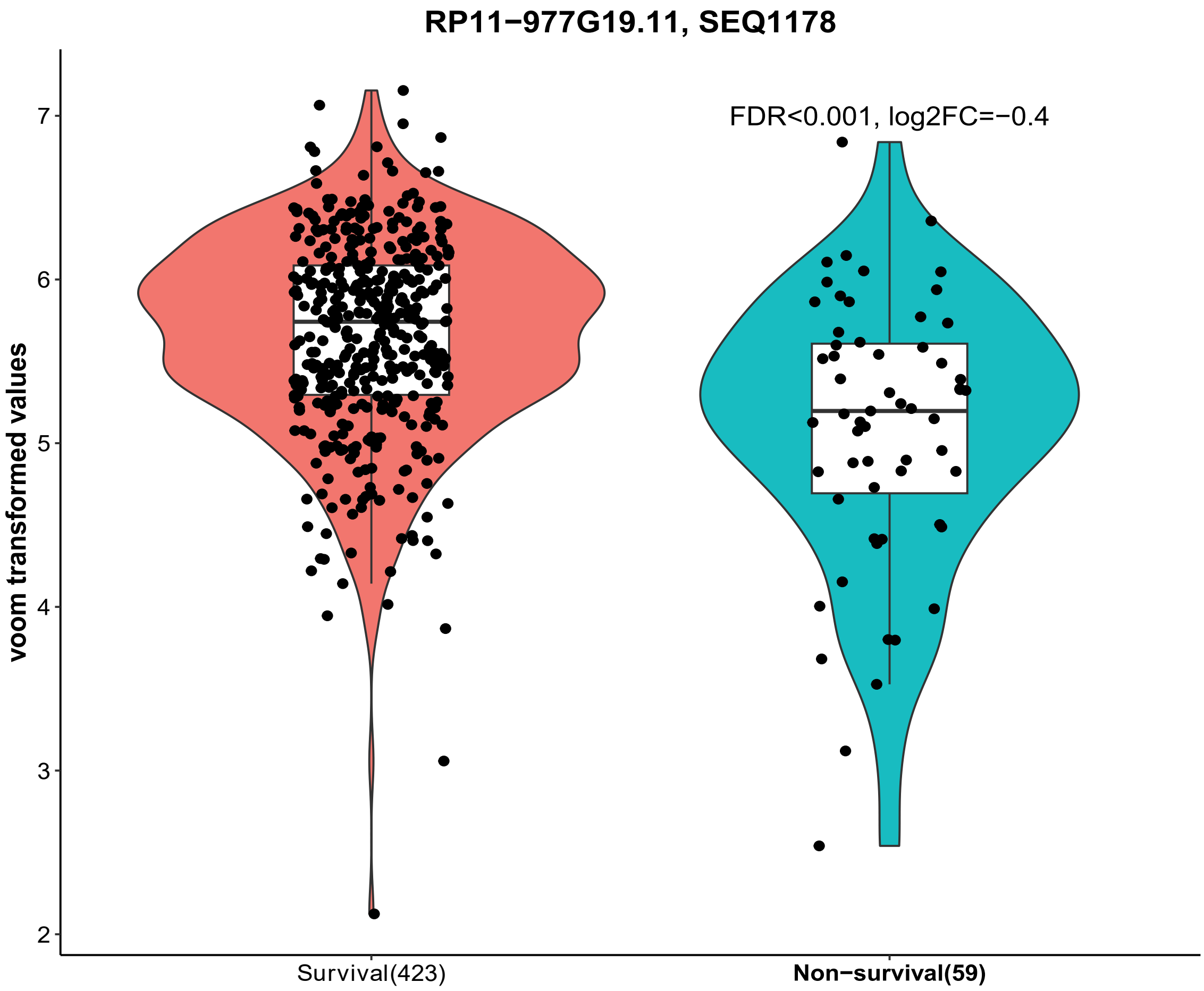

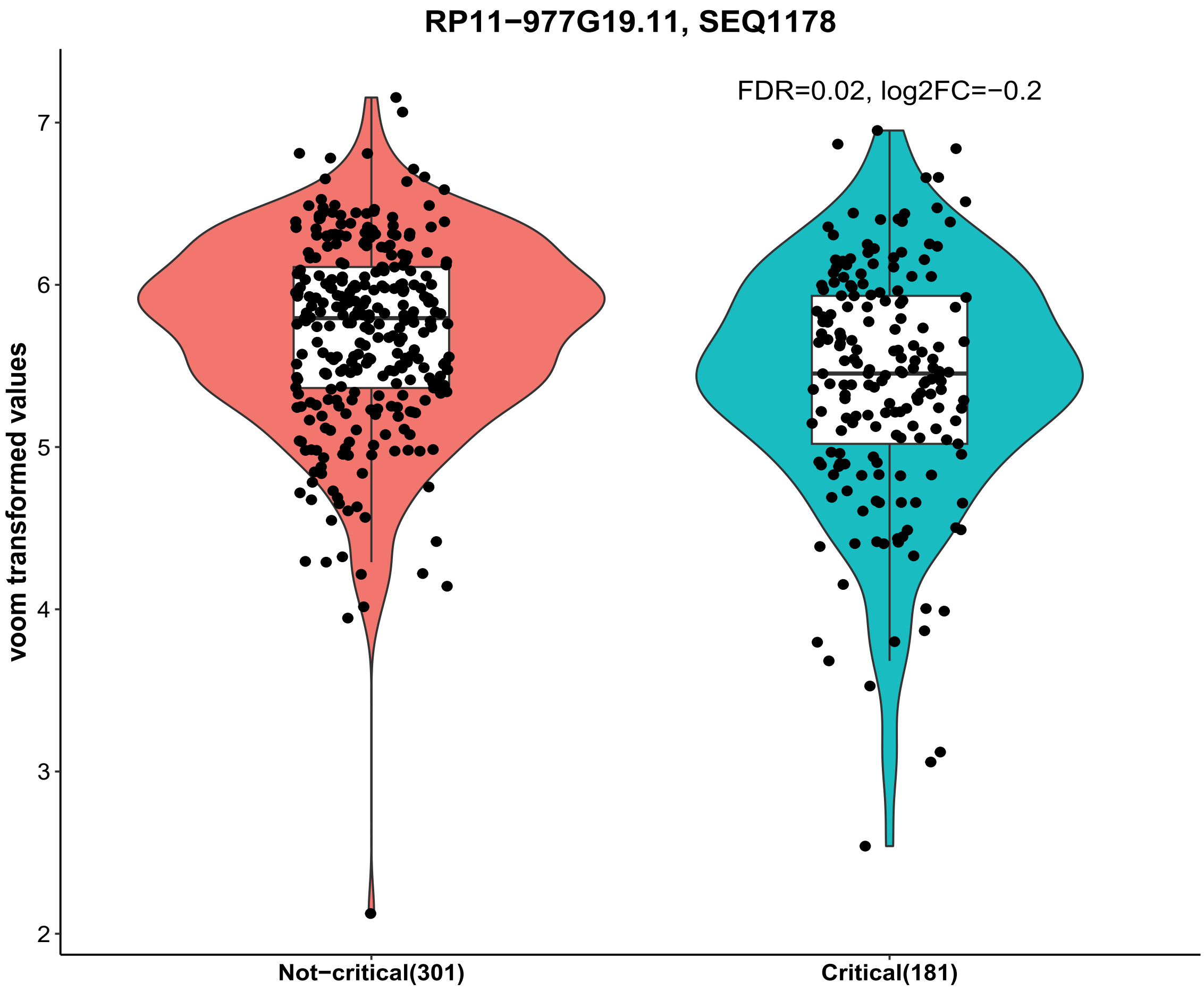

Supplement: Supplementary file 1 [file ijbsv22p7760s1.pdf]
