# Supplementary material for: Functional Genomic Screening Identifies lncRNA CNPY2-AS1 as a Regulator of Redox Metabolism and Antiviral Immunity
Source: Int J Biol Sci. 2026 Sep 3;22(14):7760–81. doi: 10.7150/ijbs.132728 (PMC13618004; doi:10.7150/ijbs.132728)
Supplement: Supplementary file 2 [file ijbsv22p7760s2.pdf]

Supplementary Tables

Table S1 | Antibodies used in this study

Antibody	Source/Catalog Number
Anti-Rabbit FITC	Laboratory of Dr. Ron-Harel
Anti-Rabbit AF488	Jackson #111-545-144
Anti-ACE2 antibody (Human, Cat, African green monkey)	Abcam ab308378
Anti-GAPDH antibody [EPR6256]	Abcam ab128915
Anti-rabbit Alexa Fluor® 594	Cell Signaling 8889
Anti-ACE2 antibody [EPR24705-45] (human)	Abcam ab272500
HRP-conjugated goat anti-rabbit IgG	Abcam ab6721
Stat1 (D1K9Y) Rabbit Monoclonal	Cell signaling #14994
Donkey Anti-Rabbit IgG H&L (Alexa Fluor® 647)	Abcam ab150063
Anti-TXNRD1 antibody [EPNCIR129]	Abcam AB124954
Phospho-IRF-7 (Ser477) (D7E1W) Rabbit Monoclonal Antibody	Cell signaling #12390
IRF-7 (D2A1J) Rabbit Monoclonal Antibody	Cell signaling #13014

Table S2 | Plasmids used in this study

Plasmid	Source
CRISPRi non-coding library (CRiNCL)	Jonathan Weissman, Addgene #86538
SARS2-Spike	Laboratory of Prof. Benjamin Podbilewicz
hACE2-Blast	Laboratory of Dr. Ori Avinoam
SARS2-Spike-Δ18	Laboratory of Prof. Alon Herschhorn
SARS2-Spike-Δ18-D614G	Laboratory of Prof. Alon Herschhorn
pHR-SFFV-dCas9-BFP-KRAB	Addgene #46911
dCas9-KRAB-mCherry-ZIM3	Addgene plasmid #154473
pHR-SFFV-KRAB-dCas9-P2A-mCherry	Addgene PLASMID #60954

psPAX	Addgene plasmid #12260
PSB700-puro	Addgene #64046
VSV-G	Addgene plasmid #8454
Lenti-ACE2-TMPRSS2-Blast	Addgene Plasmid #154983
lenti-CRISPRv2	Addgene plasmid #52961
N174-MCS	Addgene plasmid #81061

Table S3 | qPCR Primers

Target Gene	Forward Primer	Reverse Primer
hACE2 primer pair 1	tccattggtcttctgtcacccg	agaccatccacctccacttctc
hACE2 primer pair 2	acagtccacacttgcccaaat	tgagagcactgaagacccatt
RP11-977G19.11	GGAGAAGGGTGCTGATCAGG	TGCAGGCTTATTCCCCATGC
RP11-314A20.5	GTGAGGTAGCGGATCTGAGC	ACTAACGAGCGGCTCCTAGA
CNPY2	GAGGAGCCAGGATCTCCACT	TCATCATGCGATATGTGCAG
Citrate Synthase	CGTTTCCGAGGTCATAGTATCCC	GCTGAGACATAGGGTGTAGGTTGG
PAN2	ACCCTGATGGTAGCAAAAGTGAT	TGTTGCGGGTCTGAATCGTG
MED11	CTACCTCACCCAGGTGGC	TCTCCCTGGCCTAGTTCTCC
ZMYND15	TGGAGAACGAAACAAGAC	CACAAGTAAGAGACAGGAA
CXCL16	GAGCTCACTCGTCCCAATGAA	TCAGGCCCAACTGCCAGA
PLSCR1	CCTGGCAGAGAGGGGGCAAAAC	ACAACAAAGGCAGGAAACAGTGCT
TXNRD1	ACACAAAGCTTCAGCATGTCA	CAATTCCGAGAGCGTTCC
TXNIP	TGATCAAGATGCCCAACCCT	ATGGCTGAGAGTGACTGACC

Table S4 | PCR Primers

Primer	Forward	Reverse
G19 primers flanking the KO region	GAGTGCAGTGGTGCGATCTT	GTGGAGGTAAAGGCACAACG
G19 primers in the KO region	AGCTCCGACTCAGATTTTGAACG	GCTCCTCACACTTATGTTTTATTCCCAC
MED11	CAGGCGCACACCGAGAG	TCCACCCTGGATCACCCAG

PLSCR1	TGGCAAGGTTAACGTTCTGT<br>GACATTCTGAACATGGCCTTGA<br>TGCATAATTGGGCTTCTTGTAGG<br>TTTGTGGAGGTCAATGTGCC<br>GACCCCAGAGTGTCAGGATC	CATGTTTCAGGGAGCACAGG<br>ACATCCTGCACAGCCCTTAA<br>CAGCACATGTTTCAGGGAGC<br>TTCACCATGTCAGCCAGGAT<br>AATGAGGGTTACATATGGTGCA

Table S5 | gRNA Sequences

gRNA Target	Protospacer
G19- exon	AGTCATGTGGATGTGGAGAA
G19-intron	aacaacacacatgtacacaa
MED11-1	AGGTTCGGGATGCGACAACC
MED11-2	GAACGAGAGACTACGCGCTC
PLSCR1	AATTAAGTCCTTATGCCGTG<br>ATACCCCAAGTGGCATAAGG<br>AATTAAGTCCTTATGCCGTG<br>AATCATCCCTCACTTAAACG<br>AATCATCCCTCACTTAAACG<br>CATCCCTCACTTAAACGTGG<br>TATACTGGCTGATTATACAC<br>GCTCCTCCTGGTGTACCAAT<br>CCAGGATATAGTGGCTACCC

Table S6 | overexpression Sequences

Target Gene	Sequence
RP11-314A20.5	GGCTCACTGCAACCTCCACCTCCTGGGTTCAAGCGATTCTCCTGCCTCACC<br>CTTCCAAGTAGCTGGGATTACAGGTGCCCGCTACCAGCCCAGCTAATTTTT<br>GTATTTTTAGTAGAGACGGAGTTTCACCATGTTGGCCAGGCTGGTCTCGAA<br>CTCTGTCAAGCCACTGCGCCCGGCCCATTCTTTCCTTTTAGAGGAAGAAAC<br>CCGCTCTGGATGGCCGGCTGGGGAGGTCTGGTCCTGCCCAGTTAATGCTCC<br>ACCCTGGATCACCCAAGCCTGAGGCTTTGACCCAGAGCTGGGGTTCGCTC<br>GCAGAACCCCCCAGACACACCGACCTGGGTGAGGTAGCGGATCTGAGCTG<br>ACAGCTCCGCCTCCACGTGTTGCACTGAAGCGGTGAAGGCCGCCGCCTGC<br>CGGTCTAGGAGCCGCTCGTTAGTTTTTCCTTGGACAATTCTAGGATCACA
MED11	ATGGCTACCTACAGCCTGGCGAACGAGAGACTACGCGCTCTGGAAGACAT<br>TGAACGGGAAATCGGCGCCATCCTTCAGAATGCAGGTACTGTGATCCTAG<br>AATTGTCCAAGGAAAAAACTAACGAGCGGCTCCTAGACCGGCAGGCGGC<br>GGCCTTCACCGCTTCAGTGCAACACGTGGAGGCGGAGCTGTCAGCTCAGA<br>TCCGCTACCTCACCCAGGTGGCCACAGGGCAGCCCCATGAGGGCTCCAGC<br>TACTCTTCGAGGAAGGACTGTCAGATGGCTCTGAAGCGAGTGGACTATGC<br>CCGCCTCAAGCTCAGTGATGTGGCTCGAACCTGTGAGCAGATGCTGGAGA

Table S7|mass spectrometry results

Gene names	Log2 Intensity control 1	Log2 Intensity control2	Log2 Intensity control 3	Log2 Intensity control4	Log2 Intensity G19.1	Log2 Intensity G19.2	Log2 Intensity G19.3	Log2 Intensity G19.4	Student's T-test Log2 Fold Change G19_control
HMCN2	18.000	18.000	18.000	18.000	18.000	24.333	18.000	24.409	3.186
HEPHL1	18.000	18.000	21.009	18.000	18.000	18.000	23.271	27.318	2.895
TXNRD1	18.000	18.000	18.000	18.000	18.000	18.000	21.592	25.899	2.873
H1-0	18.000	18.000	18.000	18.000	18.000	18.000	20.198	27.042	2.810
CALHM4	18.000	18.000	21.530	18.000	18.000	18.000	22.651	26.797	2.479
SDR9C7	18.000	18.000	18.000	18.000	18.000	22.294	18.000	23.067	2.340
MRPS14	18.000	18.000	18.000	18.000	18.000	22.252	21.860	18.000	2.028
LCE1C;LCE1B	18.000	18.000	18.000	18.000	18.000	21.267	18.000	20.917	1.546
DDX39B;DDX39A	18.000	18.000	18.000	18.000	18.000	18.000	18.000	24.103	1.526
NOP58	18.000	18.000	18.000	18.000	18.000	18.000	18.000	22.256	1.064
EIF6	18.000	18.000	18.000	18.000	18.000	18.000	18.000	22.200	1.050
SRSF3	22.305	22.625	18.000	18.000	22.416	18.000	22.160	23.910	1.389
EIF5A;EIF5A2;EIF5AL1	18.000	22.281	22.466	22.260	21.951	21.046	21.899	25.048	1.234
CIRBP	21.467	18.000	18.000	18.000	21.972	22.168	18.000	18.000	1.168
RPL31	24.850	23.565	22.837	18.000	24.345	24.016	22.412	23.037	1.139
ACO1	22.157	18.000	18.000	18.000	18.000	26.514	18.000	18.000	1.089
EIF3F	18.000	18.000	21.014	18.000	20.145	18.000	18.000	22.727	0.965
RPL34	24.924	24.734	26.514	24.743	26.489	26.863	26.170	24.794	0.850
RPL39;RPL39P5	24.334	26.691	26.403	23.763	26.903	26.514	26.227	24.399	0.713
LSM14A	18.000	23.802	23.639	18.000	22.776	22.377	22.496	18.000	0.552
EIF3L	20.252	18.000	18.000	18.000	18.000	18.000	18.000	22.433	0.545
HNRNPAB	25.703	25.626	25.518	25.226	26.204	25.644	25.996	26.288	0.515
NOP56	20.690	18.000	18.000	18.000	18.000	18.000	18.000	22.472	0.445
HNRNPDL	25.364	26.245	25.453	24.856	25.974	26.366	25.579	24.871	0.218
RBMX	26.842	26.858	26.497	26.622	27.090	27.026	26.606	26.888	0.198
HNRNPH3	26.800	26.545	26.547	26.763	26.869	27.044	27.088	26.436	0.195
PSMA1	18.000	23.913	24.044	22.118	22.986	24.644	22.998	18.000	0.138
SNRPC	25.867	26.356	26.092	25.858	26.219	25.964	26.077	26.445	0.133
DAZAP1	24.776	25.110	25.044	24.476	24.754	24.956	24.524	25.387	0.053
RBM3	25.744	25.696	25.774	25.651	26.085	25.893	26.184	24.887	0.046
RPS7	27.225	24.021	25.578	25.947	26.354	24.636	25.719	26.221	0.040
RPS13	28.711	28.646	28.544	28.343	28.771	28.524	28.233	28.812	0.024
CPSF6	18.000	18.000	18.000	18.000	18.000	18.000	18.000	18.000	0.000
HNRNPA1;HNRNPA1L3;HNRNPA1L2	29.985	30.272	29.935	29.754	29.959	30.064	29.566	30.332	-0.007
RPL15	26.351	26.433	26.400	26.437	27.078	25.780	26.272	26.392	-0.025
HNRNPD	27.336	27.039	26.970	27.007	27.107	26.739	27.176	27.125	-0.051
HNRNPC;HNRNPCL1;HNRNPCL3;HNRNPCL2;HNRNPCL4	26.223	26.307	26.119	26.134	25.973	25.980	25.940	26.571	-0.079
SRSF1	19.969	21.199	18.000	21.120	21.318	19.940	20.652	18.000	-0.094

RPS25	25.336	24.667	24.383	25.229	24.988	24.144	24.727	25.355	-0.100
HNRNPF	27.108	27.377	27.045	27.295	26.802	27.039	27.031	27.486	-0.117
RPL18A	27.959	27.861	27.866	27.675	27.806	27.839	27.411	27.824	-0.120
RPL30	24.755	24.361	25.290	25.400	24.741	24.519	24.693	25.295	-0.139
HNRNPA2B1	30.557	30.574	30.260	30.454	30.529	30.374	30.133	30.232	-0.144
EEF1A1;EEF1A1P5	28.405	28.150	28.503	28.625	27.907	27.467	27.616	30.101	-0.148
HNRNPH1	28.406	28.681	28.546	28.575	28.552	28.401	28.296	28.334	-0.156
EEF2	25.678	25.006	26.003	25.897	24.201	24.450	24.671	28.592	-0.168
RPL21	27.730	27.869	27.622	27.403	27.271	27.372	27.653	27.583	-0.186
RBM38;RBM24	18.000	21.299	18.000	22.081	21.520	21.095	18.000	18.000	-0.191
U2AF1;U2AF1L4	21.914	23.892	23.408	24.385	24.324	21.960	22.930	23.618	-0.192
SNRPD3	24.613	25.019	24.876	24.936	24.543	24.688	24.402	25.019	-0.198
HNRNPA3	30.148	30.232	30.139	30.101	29.811	29.818	29.996	30.204	-0.198
RPL27A	28.891	27.972	28.276	28.370	28.544	28.154	27.915	28.055	-0.210
FUS	27.518	27.689	27.735	27.512	27.488	27.327	27.492	27.300	-0.212
RBM14	26.256	26.000	26.434	25.972	25.874	25.955	25.543	26.382	-0.227
RPL4	27.346	27.037	27.084	27.136	27.117	26.434	26.732	27.400	-0.230
EEF1G	25.464	25.325	26.294	25.566	25.004	24.639	24.981	27.096	-0.233
RPL14	26.078	25.918	26.138	26.105	25.818	24.586	25.613	27.227	-0.248
RPL24	27.263	27.171	26.987	26.987	26.712	26.707	26.560	27.429	-0.250
RPL35	28.260	28.007	28.072	27.953	28.118	28.061	27.687	27.392	-0.259
RPL38	24.194	25.844	25.313	25.960	24.262	24.476	24.641	26.896	-0.259
RPLP2	26.437	26.719	27.056	26.895	26.274	26.007	26.394	27.385	-0.262
YBX1	28.637	29.165	28.918	28.712	28.927	28.904	28.481	28.051	-0.268
HNRNPU	29.126	29.108	29.110	29.195	29.006	29.064	28.739	28.604	-0.282
RPL6	26.526	25.919	26.120	26.043	25.787	25.883	25.598	26.202	-0.284
RPS11	28.344	27.918	28.214	28.382	27.971	27.896	27.494	28.155	-0.335
RPL13	29.081	28.892	28.978	28.746	29.093	28.567	28.285	28.408	-0.336
SNRPE	23.553	23.944	24.052	24.114	23.568	23.101	23.258	24.237	-0.375
EWSR1	25.134	25.195	25.383	25.164	24.510	24.933	25.381	24.535	-0.380
RALY	24.945	24.068	24.506	24.737	24.515	24.462	24.533	23.208	-0.385
ANXA1	26.682	26.205	26.521	26.055	25.407	24.976	26.856	26.576	-0.412
VIM	33.300	33.373	33.352	33.275	32.906	32.781	32.937	32.976	-0.425
RPL18	26.240	26.129	26.296	25.335	25.183	25.051	25.633	26.429	-0.426
RPL29	24.102	25.909	21.613	18.000	18.000	26.792	25.046	18.000	-0.446
TIAL1	24.339	24.750	24.210	24.939	24.378	24.411	24.354	23.260	-0.459
RPL8	28.530	29.163	28.828	28.953	29.198	28.389	28.409	27.630	-0.462
TUBB4B;TUBB4A	29.072	28.999	29.369	28.936	28.321	27.979	28.030	29.967	-0.520
RPS4X	27.800	27.591	27.653	27.499	27.303	26.866	27.003	27.269	-0.525
PABPC1	27.239	27.072	27.524	27.222	26.408	25.326	26.974	28.232	-0.529
ELAVL1	21.235	21.172	18.000	18.000	18.000	18.000	18.000	22.263	-0.536
TUBA1B	25.150	25.051	25.075	25.040	24.263	23.994	24.220	25.660	-0.545
RPS15A	24.723	25.055	24.845	25.347	24.464	22.588	24.408	26.321	-0.547
RPS2	25.832	25.800	25.813	26.435	26.040	24.819	24.287	26.400	-0.583
RPS26;RPS26P11	28.294	28.182	27.728	27.831	28.399	27.916	28.113	25.205	-0.601
EIF3D	24.854	24.903	24.303	24.837	24.772	23.654	24.512	23.386	-0.643
HSP90AB1	26.571	26.224	26.368	26.916	25.673	25.409	25.278	27.048	-0.668
HNRNPK	27.033	27.467	27.177	27.083	26.912	26.269	26.511	26.371	-0.674
EIF4A1;EIF4A2	26.189	25.597	26.057	25.646	24.647	23.778	23.946	28.400	-0.680
CAVIN1	25.229	25.096	24.823	25.297	24.647	24.861	23.847	24.262	-0.707

RAE1	24.637	25.190	24.660	24.331	24.013	24.125	24.542	23.164	-0.744
RPL28	24.722	24.653	24.409	25.292	24.583	22.709	23.780	25.008	-0.749
EIF3A	18.000	18.000	22.437	21.610	18.000	18.000	18.000	22.962	-0.771
RPS3	24.834	25.315	24.981	24.790	24.028	22.853	23.601	26.326	-0.778
RPS9	23.281	23.156	23.746	20.819	18.000	22.266	23.640	23.960	-0.784
LRPPRC	18.000	18.000	18.000	21.160	18.000	18.000	18.000	18.000	-0.790
RPS16	26.914	26.946	26.930	26.495	26.558	25.824	25.833	25.908	-0.791
HSPD1	26.234	26.429	26.040	27.399	25.706	25.609	25.122	26.501	-0.791
RPS3A	25.872	25.379	25.866	26.280	25.070	24.967	24.937	25.217	-0.802
RBM4;RBM4B	23.150	23.784	24.301	24.250	23.512	23.624	22.735	22.397	-0.804
DDX21	18.000	18.000	18.000	21.289	18.000	18.000	18.000	18.000	-0.822
SNRNP70	22.283	24.646	24.202	23.009	23.254	23.164	22.144	22.274	-0.826
RPL23A	25.034	24.958	24.744	24.852	24.441	23.729	24.646	23.385	-0.847
FBL	25.524	25.629	25.751	25.473	24.840	23.998	24.738	25.350	-0.863
RPL7A	25.946	25.495	26.046	25.687	23.883	25.116	24.732	25.904	-0.885
EEF1D	26.790	27.110	28.444	26.845	26.468	26.452	26.169	26.550	-0.887
RPS5	26.257	25.860	26.244	25.912	24.957	25.180	24.309	26.265	-0.890
RPS18	26.253	25.904	25.599	25.797	25.299	25.146	24.332	25.123	-0.913
YBX3	24.693	21.979	23.736	24.556	24.557	24.720	23.945	18.000	-0.935
TUFM	23.154	18.000	23.131	21.275	20.770	18.000	21.407	21.618	-0.941
RPL5	25.631	25.220	25.483	25.221	23.376	23.124	24.314	26.943	-0.950
HNRNPL	25.918	26.038	25.561	25.980	25.601	25.168	24.955	23.962	-0.953
RPL7	24.940	25.350	25.518	25.029	23.380	23.180	24.481	25.879	-0.979
HNRNPA0	25.808	25.749	25.518	25.546	24.263	24.694	25.431	24.228	-1.001
SNRPD1	22.179	18.000	22.203	18.000	18.000	22.317	18.000	18.000	-1.016
RPL19	24.495	24.894	24.654	24.343	23.498	23.113	24.255	23.230	-1.073
RPS14	25.951	25.524	25.665	25.710	24.488	25.008	23.227	25.799	-1.082
RPL3	26.627	26.651	26.585	26.497	26.341	25.885	26.089	23.700	-1.086
HNRNPM	27.028	27.449	27.230	27.212	26.365	25.847	26.237	25.815	-1.164
RBFOX2;RBFOX1	22.892	23.750	23.031	22.886	23.694	22.839	18.000	23.349	-1.169
HNRNPR	26.571	27.250	26.895	26.250	25.795	25.581	25.660	25.164	-1.192
MRPS17	22.776	18.000	18.000	18.000	18.000	18.000	18.000	18.000	-1.194
RPL9	24.919	24.439	24.457	24.756	24.126	22.041	23.311	24.159	-1.233
ILF3	27.532	27.951	27.775	27.619	25.999	26.373	27.103	26.410	-1.248
PCBP2	21.654	21.631	21.935	18.000	18.000	18.000	18.000	24.182	-1.260
MRPS7	21.533	18.000	21.532	21.827	18.000	20.652	21.159	18.000	-1.270
RPL26	26.053	25.438	25.599	26.182	24.538	25.102	23.121	25.305	-1.302
HSP90AA1	25.618	24.651	25.433	25.193	23.431	23.760	23.343	25.032	-1.332
NOP10	22.681	22.896	22.943	23.737	22.942	23.125	22.820	18.000	-1.342
SRP14	23.111	23.074	23.070	22.940	18.000	22.734	22.767	23.267	-1.357
EIF5	18.000	21.874	22.431	18.000	18.000	20.870	18.000	18.000	-1.359
SF1	23.186	24.424	24.783	24.839	21.547	22.921	23.975	23.217	-1.393
SNU13	21.041	18.000	18.000	20.620	18.000	18.000	18.000	18.000	-1.415
DDX17	24.566	25.518	25.075	25.101	24.247	23.444	24.413	22.455	-1.425
MRPL23	23.786	23.823	23.665	23.616	23.755	23.657	23.765	18.000	-1.428
DDX5	27.002	27.150	27.399	27.121	26.109	24.854	26.169	25.785	-1.439
RPLP0;RPLP0P6	24.986	24.270	25.225	25.115	22.905	22.485	22.250	26.134	-1.455
PABPN1	18.000	23.053	22.727	23.627	22.875	18.000	22.580	18.000	-1.488
SNRPB;SNRPN	22.668	23.404	22.916	21.967	21.436	18.000	22.732	22.515	-1.568
TARDBP	22.967	24.015	24.542	23.825	24.218	22.697	24.023	18.000	-1.602
AGFG1	22.844	23.202	23.136	22.999	22.860	22.169	22.701	18.000	-1.613

EIF2S3;EIF2S3B	18.000	22.266	21.367	21.695	18.000	18.000	18.000	22.852	-1.619
PABPC4	18.000	22.965	23.447	23.715	21.900	18.000	23.574	18.000	-1.663
HNRNPH2	24.698	24.097	24.289	24.951	24.670	24.538	24.145	18.000	-1.671
PSMA6	18.000	22.450	21.372	22.377	18.000	18.000	18.000	23.483	-1.679
EIF3I	22.063	23.281	21.866	22.046	21.229	21.351	21.846	18.000	-1.708
STAU1	22.108	18.000	20.553	20.682	18.000	18.000	20.166	18.000	-1.794
EIF2S1	22.209	20.981	21.132	21.298	20.485	18.000	18.000	21.883	-1.813
DDX3X;DDX3Y	24.588	25.609	25.515	25.190	24.083	23.446	22.288	23.785	-1.825
RPL22	24.994	25.038	24.347	25.132	23.103	22.538	22.424	24.059	-1.847
RPL11	23.667	24.771	23.809	24.722	23.366	18.000	23.019	25.171	-1.853
NCL	20.795	20.817	27.602	21.154	22.104	21.997	20.816	18.000	-1.863
PCBP1	24.572	23.898	23.623	24.998	22.717	18.000	23.141	25.696	-1.884
MYH4	18.000	25.925	18.000	18.000	18.000	18.000	18.000	18.000	-1.981
DHX15	18.000	21.145	21.145	19.835	18.000	18.000	18.000	18.000	-2.031
CAPRIN1	24.272	24.501	24.484	24.278	22.539	22.173	23.236	21.441	-2.037
CLTC	26.824	26.898	27.339	26.090	23.866	23.432	25.233	26.099	-2.130
CRYZ	22.388	21.216	21.878	21.419	21.626	20.754	18.000	18.000	-2.130
TRA2B	24.088	24.025	24.742	23.904	23.300	23.668	23.219	18.000	-2.143
HNRNPUL1	24.807	24.423	24.744	24.526	23.219	23.892	24.196	18.000	-2.298
RPL35A	23.554	24.612	22.316	22.155	18.000	18.000	23.627	23.706	-2.326
KHSRP	24.715	26.014	25.604	25.669	23.550	23.413	21.992	23.707	-2.335
RPLP1	26.347	26.202	26.519	26.399	26.113	25.670	25.898	18.000	-2.447
G3BP1	21.904	21.898	23.239	21.904	19.882	18.000	18.000	22.552	-2.628
ILF2	23.723	24.455	23.571	22.836	20.987	18.000	21.154	23.932	-2.628
TIA1	23.060	22.996	19.702	18.000	18.000	19.194	18.000	18.000	-2.641
MBNL1	22.499	22.759	21.447	22.245	23.331	18.000	18.000	18.000	-2.905
PTBP1	23.967	23.183	23.228	23.246	18.000	18.000	22.211	22.916	-3.125
FAU	23.924	23.449	24.241	24.977	23.829	24.062	18.000	18.000	-3.175
NUDT21	22.703	21.759	21.145	21.709	18.000	18.000	20.269	18.000	-3.262
PARK7	23.401	18.000	22.501	22.638	19.408	18.000	18.000	18.000	-3.283
SYNCRIP	21.960	25.776	25.002	23.959	18.000	21.624	24.819	18.000	-3.563
HSP90B1	18.000	21.830	23.136	24.300	18.000	18.000	18.000	18.000	-3.816
NPM1	24.422	25.079	25.588	25.029	23.963	23.309	18.000	18.000	-4.212
ACTN1	21.693	23.594	23.402	22.533	18.000	18.000	18.000	18.000	-4.805
